# Using PhyloSuite for molecular phylogeny and tree‐based analyses

**DOI:** 10.1002/imt2.87

**Published:** 2023-02-16

**Authors:** Chuan‐Yu Xiang, Fangluan Gao, Ivan Jakovlić, Hong‐Peng Lei, Ye Hu, Hong Zhang, Hong Zou, Gui‐Tang Wang, Dong Zhang

**Affiliations:** ^1^ State Key Laboratory of Grassland Agro‐Ecosystems, and College of Ecology Lanzhou University Lanzhou China; ^2^ Institute of Plant Virology, Fujian Agriculture and Forestry University Fuzhou China; ^3^ Key Laboratory of Aquaculture Disease Control, Ministry of Agriculture, and State Key Laboratory of Freshwater Ecology and Biotechnology, Institute of Hydrobiology, Chinese Academy of Sciences Wuhan China

**Keywords:** annotation, concatenation, iTOL, loci, multiple‐sequence alignment, partitioning, trimming

## Abstract

Phylogenetic analysis has entered the genomics (multilocus) era. For less experienced researchers, conquering the large number of software programs required for a multilocus‐based phylogenetic reconstruction can be somewhat daunting and time‐consuming. PhyloSuite, a software with a user‐friendly GUI, was designed to make this process more accessible by integrating multiple software programs needed for multilocus and single‐gene phylogenies and further streamlining the whole process. In this protocol, we aim to explain how to conduct each step of the phylogenetic pipeline and tree‐based analyses in PhyloSuite. We also present a new version of PhyloSuite (v1.2.3), wherein we fixed some bugs, made some optimizations, and introduced some new functions, including a number of tree‐based analyses, such as signal‐to‐noise calculation, saturation analysis, spurious species identification, and etc. The step‐by‐step protocol includes background information (i.e., what the step does), reasons (i.e., why do the step), and operations (i.e., how to do it). This protocol will help researchers quick‐start their way through the multilocus phylogenetic analysis, especially those interested in conducting organelle‐based analyses.

## Highlights

- A new release of PhyloSuite, capable of conducting tree‐based analyses.
- Detailed guidelines for each step of phylogenetic and tree‐based analyses, following the “What, Why, and How” structure.
- This protocol will help beginners learn how to conduct multilocus phylogenetic analyses and help experienced scientists improve their efficiency.

## Graphical abstract

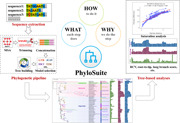

## INTRODUCTION

Molecular phylogenetics aims to reconstruct the evolutionary history of life using genetic markers, such as nucleotide and amino acid sequences [1]. Aside from the traditional objective of inferring evolutionary relationships among different lineages, the rapid generation of genomic data in the last two decades has facilitated the application of molecular phylogenetics to various aspects of biological sciences, such as population changes, migration patterns, the adaptive evolution of species associated with specific environments, and etc. [2]. For example, the application of multilocus phylogeny (inferring phylogenetic trees using multiple loci or genes) has provided new insights into many historically controversial relationships as well as contributed to our understanding of the evolutionary history of life on earth [3]. A large number of molecular phylogenetics‐related algorithms and software programs have been developed to address these developments, but they are often complicated and confusing for beginners, experimental biologists, and in general insufficiently computer‐savvy researchers. Furthermore, conducting multilocus phylogenetic analyses comprises a large number of steps: selecting and downloading sequences, filtrating sequences, preparing gene sequences (extracting genes from a multilocus dataset, such as organelle genomes; or identifying orthologous loci from genomic data), sequence alignment, alignment trimming (optional), alignment concatenation, selecting optimal partitioning schemes, selecting optimal substitution models, phylogenetic tree inference (often using several different algorithms to assess the topological stability), and finally visualization and annotation of the phylogenetic tree. For each of these steps, there are commonly different software programs available to choose from. For example, for sequence alignment, there are MAFFT [4], MUSCLE [5], PRANK [6], and etc.; for phylogenetic tree reconstruction, commonly used programs are IQ‐TREE [7], RAxML [8, 9], MrBayes [10], etc. Finally, these programs often use very different input and output file formats. This maze of steps, algorithms, and file formats can be daunting and time‐consuming for many scientists.

PhyloSuite [11] was designed with the aim to make state‐of‐the‐art multilocus phylogenetic analyses more accessible to scientists who would otherwise find it difficult, but it is also suitable for scientists proficient in conducting multilocus phylogenetic analyses who simply need a more streamlined and less time‐consuming way to conduct their analyses. It is a multifunctional GUI‐based software that incorporates all of the above functions for multilocus phylogenetic reconstruction (except for the selection of orthologous genes). It is also well‐suited for single‐locus phylogenetic inference. The comparative advantages of PhyloSuite comprise a user‐friendly graphical interface, no programming skills requirements, phylogenetic analyses in a workflow manner, batch and multithreading operations, and many others (for details of novelty/functions in comparison to other similar software programs please see Zhang et al. [11]). Because of these features, PhyloSuite is suitable both for beginners who wish to quickly learn the skills needed to conduct high‐quality phylogenetic analyses (it includes example datasets and links to more detailed explanations) and for experienced researchers who merely wish to speed up their analyses and increase productivity. In this protocol, we introduce and release an updated version of PhyloSuite (1.2.3) with a series of optimizations and new functions. In detail, for the new version we have: (1) sped up several functions, such as file extraction, codon alignment in MAFFT, sequence format conversion, concatenation, and summary function in MrBayes; (2) fixed some bugs, such as the “memory error” in MrBayes, problems while setting up PhyloSuite in Linux, error for the automatic update checking, and etc.; (3) added a lot of new functions, such as the plot function (for several different analyses), removing thirrd codon site for phylogeny, 11 tree‐based statistical analyses, and etc. For detailed information about the new version, see https://github.com/dongzhang0725/PhyloSuite/releases/tag/1.2.3. For each step of the phylogenetic pipeline (based on either multiple genes or a single gene) as well as the tree‐based statistics, we will first describe the background information of the step (i.e., what it is), then outline the reasons for doing this step (i.e., why do it), and finally present an elaborate tutorial for the operation and parameter settings of the step (i.e., how to do it). The goal of this protocol is to help users understand the “what, why, and how”, for each step of the molecular phylogenetic analysis procedure. Ultimately, we hope that it will help beginners to quick‐start their way through phylogenetic analysis.

## DATASET INTRODUCTION

To make a comprehensive tutorial for molecular phylogenetic reconstruction using PhyloSuite, herein we will use the *18S* gene of 24 ciliates (phylum Ciliophora) for the demo of a single‐gene phylogeny, and use the complete mitogenomes of nine species belonging to the order Gyrodactylidea (phylum Platyhelminthes: class Monogenea) for the demo of a multiple‐gene phylogeny. Both demos will use Maximum Likelihood (ML) and Bayesian Inference (BI) algorithms to reconstruct phylogenetic trees. For the multiple‐gene phylogeny, we adopted three strategies: (1) combine the nucleotide sequences of all protein‐coding genes (PCGs) and two rRNA genes (PCGsRNA); (2) split PCGs by codon sites, remove the third site (to reduce substitution saturation), combine the remaining (first and second codon site) nucleotide sequences of all PCGs and the complete sequences of two rRNA genes (PCGs12RNA); (3) combine the amino acid sequences of all PCGs (AA).

## INSTALLATION

The tutorial for the installation of PhyloSuite can be found here: http://phylosuite.jushengwu.com/dongzhang0725.github.io/installation/ or https://dongzhang0725.github.io/installation/.

## PHYLOSUITE INTERFACE

**Figure 1:**
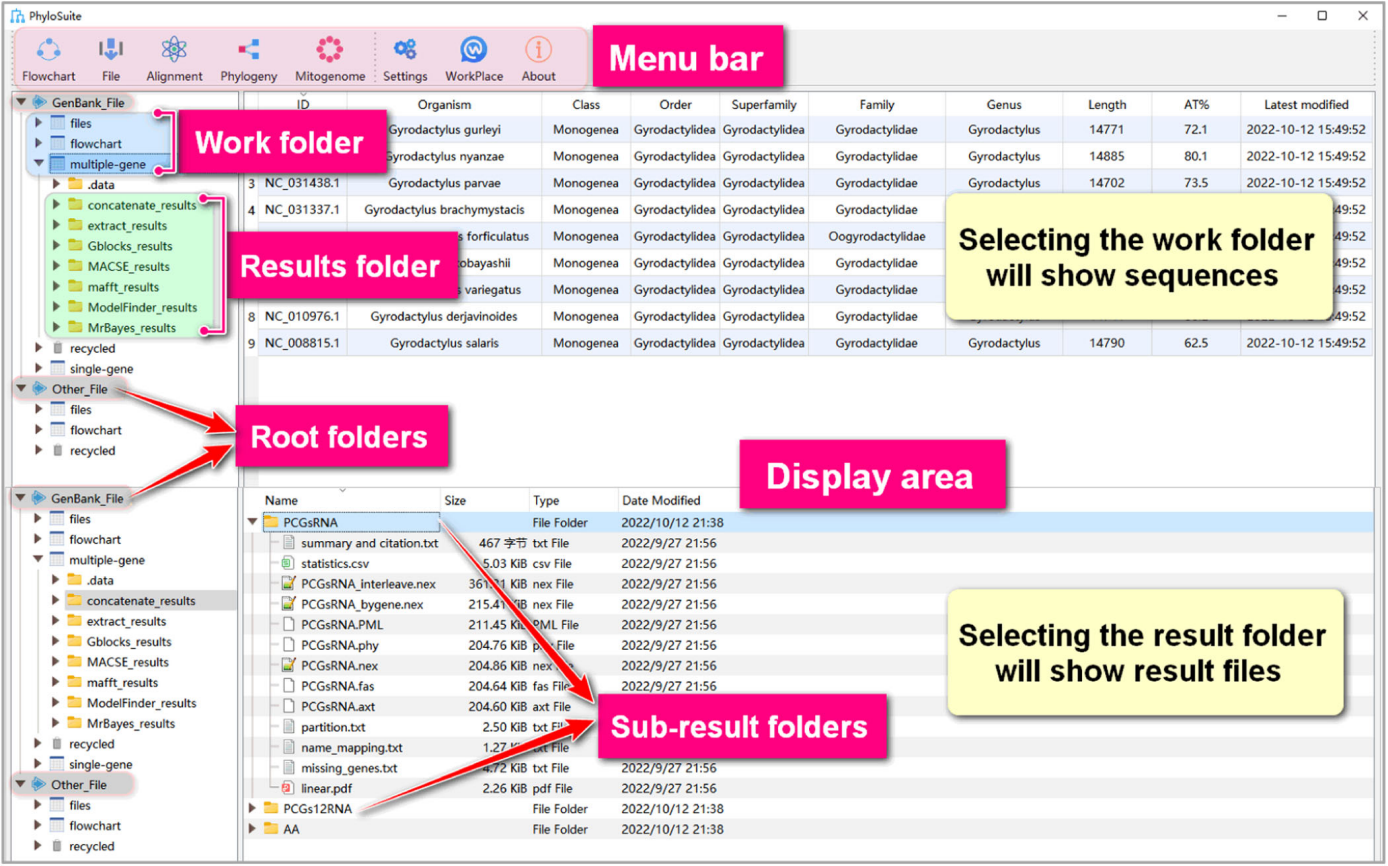
Introduction to the PhyloSuite interface.

In Figure 1, the red box highlights the menu bar, where you can select various functions or plugins, and set/change the workspace. The grey boxes highlight root folders: “GenBank_File” stores input files in the GenBank format and related results, and “Other_File” stores input files in other formats (e.g., Fasta) and their results. The blue box highlights working folders. The green box highlights the results folder. Each function in the workspace corresponds to a top‐level results folder; for example, “mafft_results.” The area to the right is used to display sequences and results. The top‐level results can contain many subfolders, the names of which can be set in the start button drop‐down menu before running the program. The default name is the current time. For further details, see the PhyloSuite manual: http://phylosuite.jushengwu.com/dongzhang0725.github.io/documentation/.

## MULTI‐GENE PHYLOGENY

There is a tutorial video for the multi‐gene phylogeny pipeline and tree‐based analyses described below, see http://phylosuite.jushengwu.com/dongzhang0725.github.io/PhyloSuite-demo/videoes/.

### Sequence download and preparation

**Figure 2:**
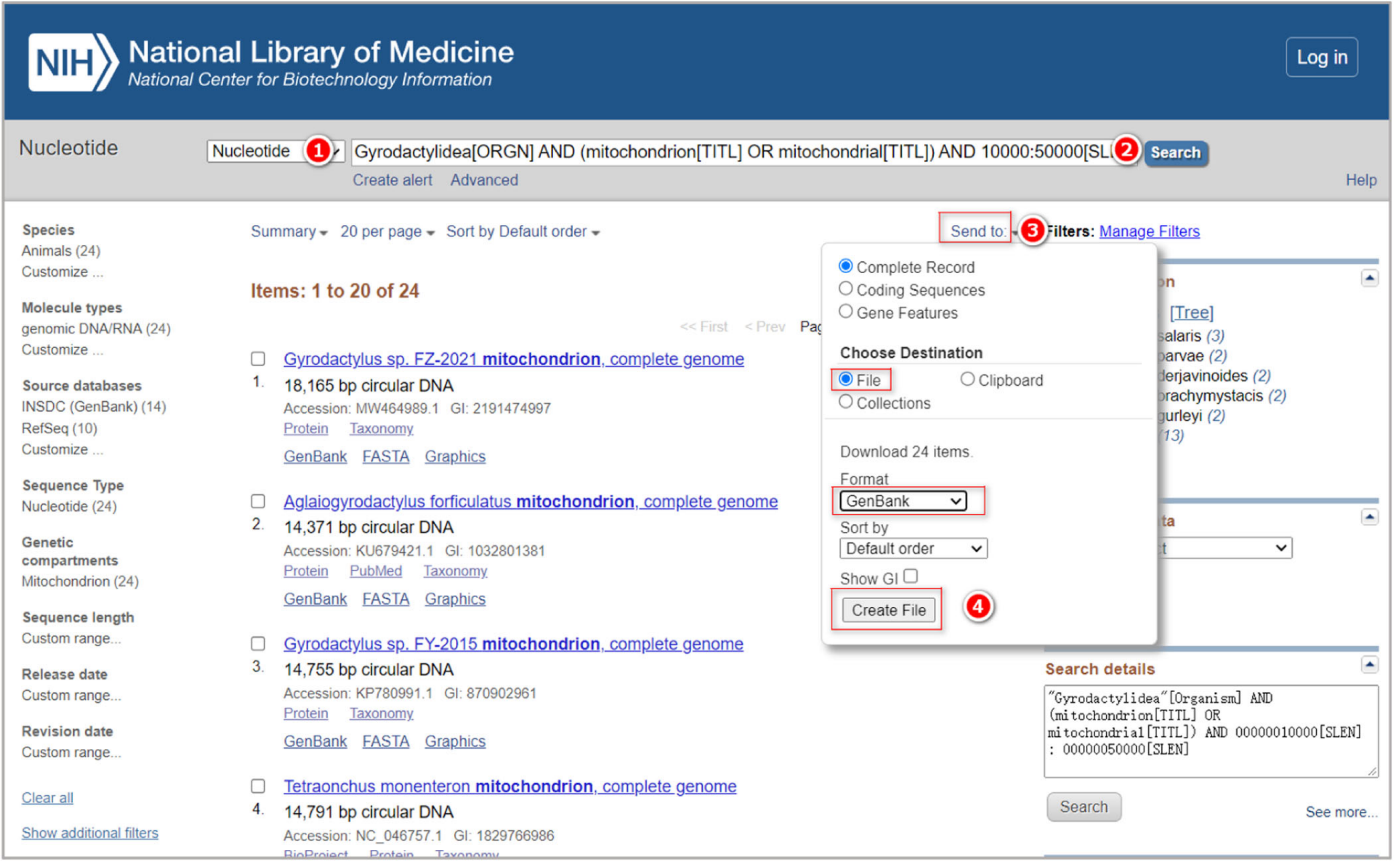
Downloading mitogenome data from the NCBI's Nucleotide database.

Generally, mitogenome data are deposited in the NCBI's nucleotide database (GenBank). We will download the data following the steps below (Figure 2).

1.1.1 Enter the NCBI's official website (https://www.ncbi.nlm.nih.gov/), and choose the “Nucleotide” database. Note that the numbers of the last level of the section title refer to the red numbers in the figure (e.g., section number 1.1.1 refers to the red colored number ① in the figure).

1.1.2 Enter “Gyrodactylidea[ORGN] AND (mitochondrion[TITL] OR mitochondrial[TITL]) AND 10000:50000[SLEN]” into the search box to search for the target sequences. These keywords can be divided into three parts. The first part is “Gyrodactylidea[ORGN]”, which limits the taxonomic group to Gyrodactylidea. The second part is “mitochondrion[TITL] OR mitochondrial[TITL]”, which limits the sequence type to mitochondrial DNA. The last part (10000:50000[SLEN]) limits the sequence length range to 10,000 – 50,000 bases. These parameters can be modified according to your own needs.

1.1.3 Choose “send to” to save all sequences.

1.1.4 Select “Complete Record” → “File” → “GenBank” → “Create File” to save all the sequences to a file.

### Import the sequences into PhyloSuite

Before importing sequences into PhyloSuite, we will create a new work folder where we will store our data and results.

**Figure 3:**
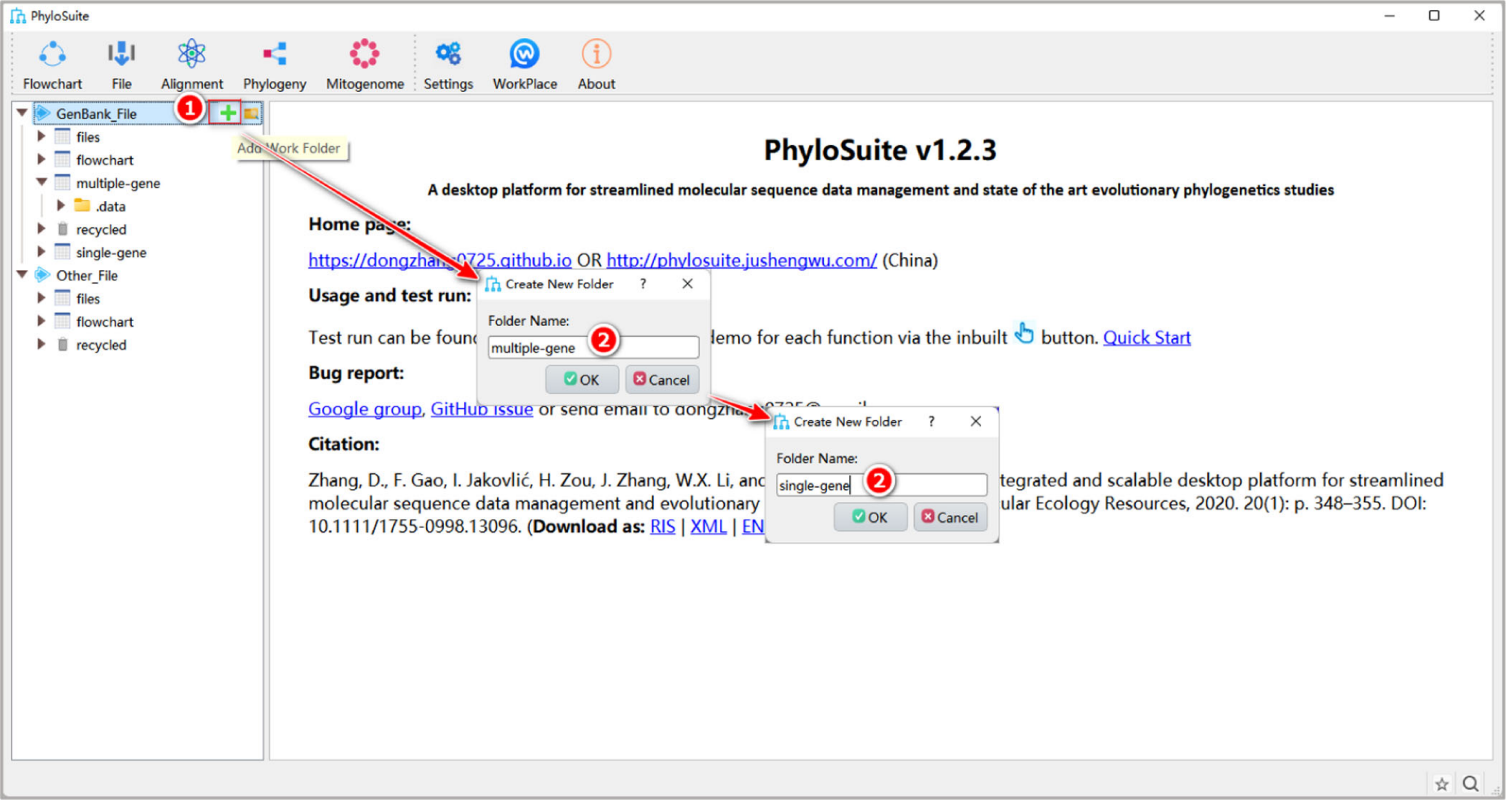
Creating a new work folder in PhyloSuite.

1.2.1 Hover the mouse over the “GenBank_File” root folder in the left panel, then click the “+” button to the right of the GenBank_File folder to create a new folder and name it (Figure 3). Alternatively, you can select any existing working folder under the “GenBank_File” root folder as your work folder (e.g., “files” folder).

1.2.2 In this protocol, we will create a work folder named “multiple‐gene” for the multilocus phylogenetic analysis, and create a work folder named “single‐gene” for the analysis of the *18S* dataset (Figure 3).

**Figure 4:**
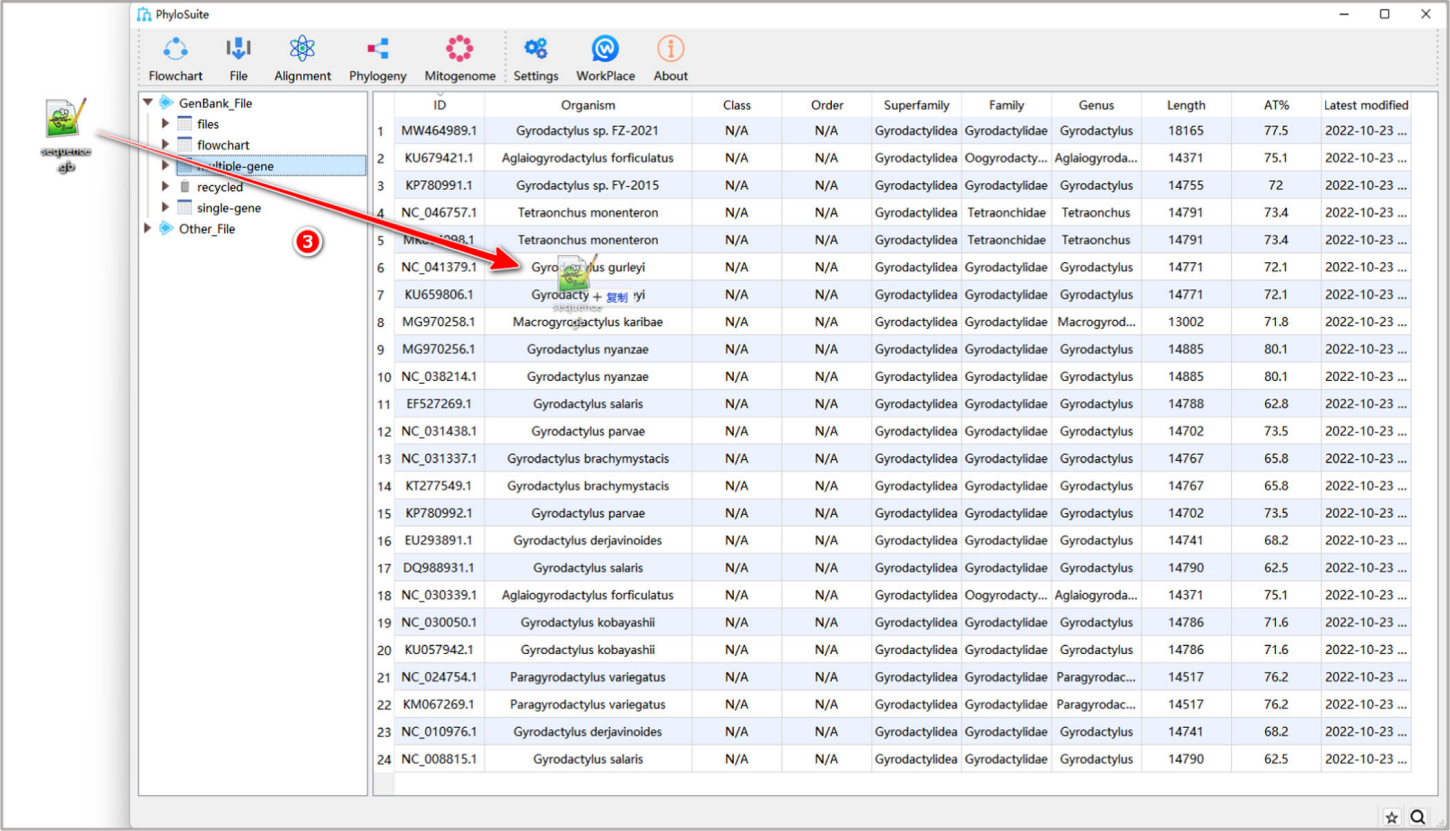
Importing the sequences into the PhyloSuite work folder.

1.2.3 Drag the sequence file (*.gb) downloaded in Step 1.1.4 into the PhyloSuite display area (the area shown in Figure 4) and drop it there to import the sequences.

### Remove redundant sequences

**Figure 5:**
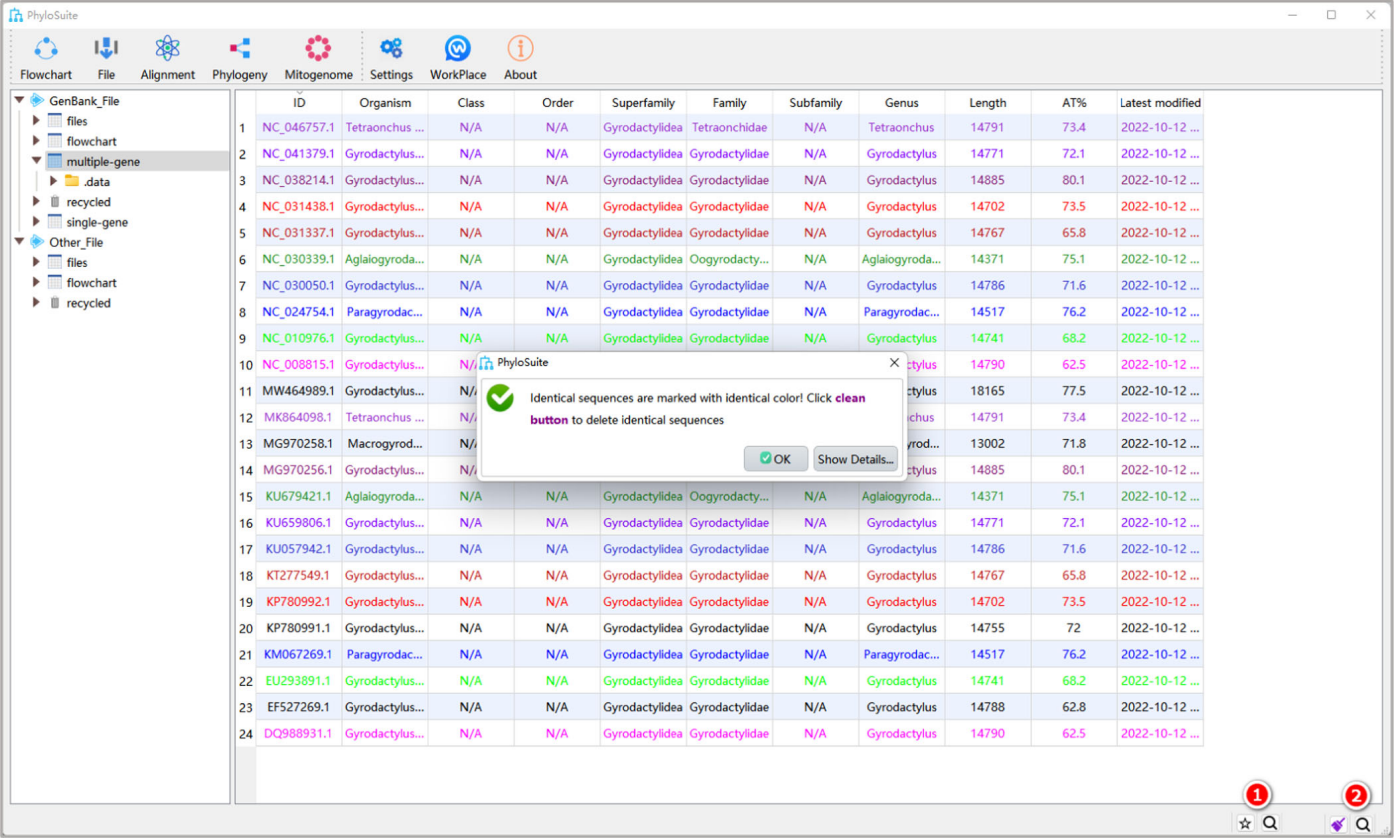
Filtering redundant sequences.

If the mitogenome passed the RefSeq database [12] screening, it will normally have two accession numbers, so we need to remove redundant sequences before starting downstream analyses. We designed such a function for PhyloSuite (Figure 5).

1.3.1 After importing sequences, click the star button in the lower right corner of the interface. A message box will appear, prompting that identical sequences are marked with the same color.

1.3.2 The five‐pointed star has now turned into a broom icon. When you click it, the identical sequences will be cleaned. Accession numbers starting with NC (RefSeq) will be retained preferentially.

In addition, you can also manually prune sequences according to your needs (such as sequences with wrong or duplicated organism names, sequences with weird composition, sequences with no annotations, etc.). Just select the sequence, right‐click, and select “delete.” Note, remember to confirm that the outgroup sequences and your own sequences (sequenced by you) are on the list.

**Figure 6:**
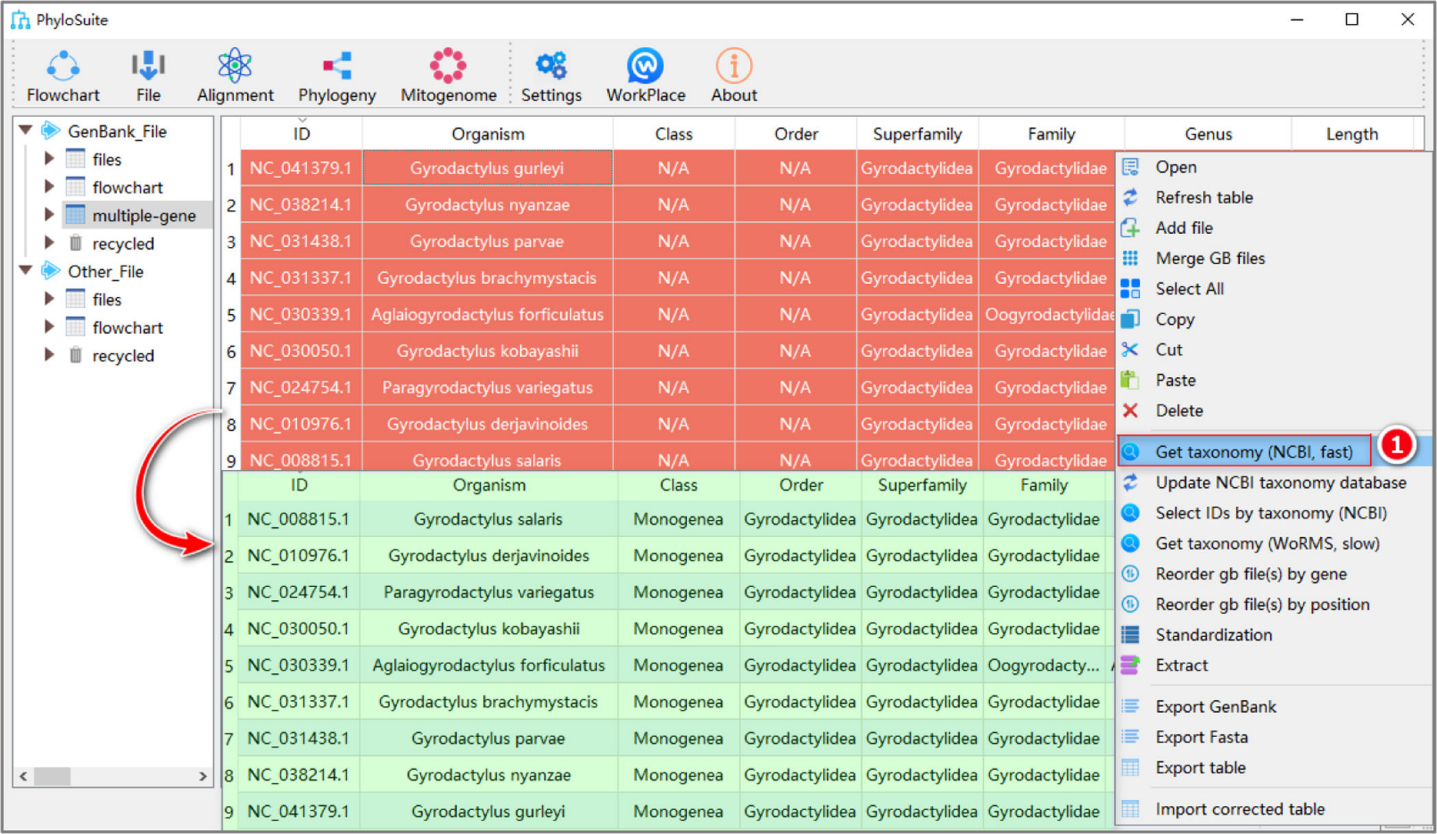
Get taxonomy.

In some cases, taxonomic information for your sequences, which is automatically retrieved from the downloaded GenBank files, may be incomplete or even wrong. PhyloSuite allows users to retrieve updated taxonomic information from the NCBI's Taxonomy database [13] or from the WORMS database [14]. Select the sequences, then right‐click to pop‐up the context menu, and select “Get taxonomy (NCBI, fast)” or “Get taxonomy (WoRMS, slow)” to get taxonomic information from the NCBI or WORMS databases respectively (Figure 6). In addition, you can also directly double‐click table cells to edit the taxonomic information manually.

### Sequence extraction

**Figure 7:**
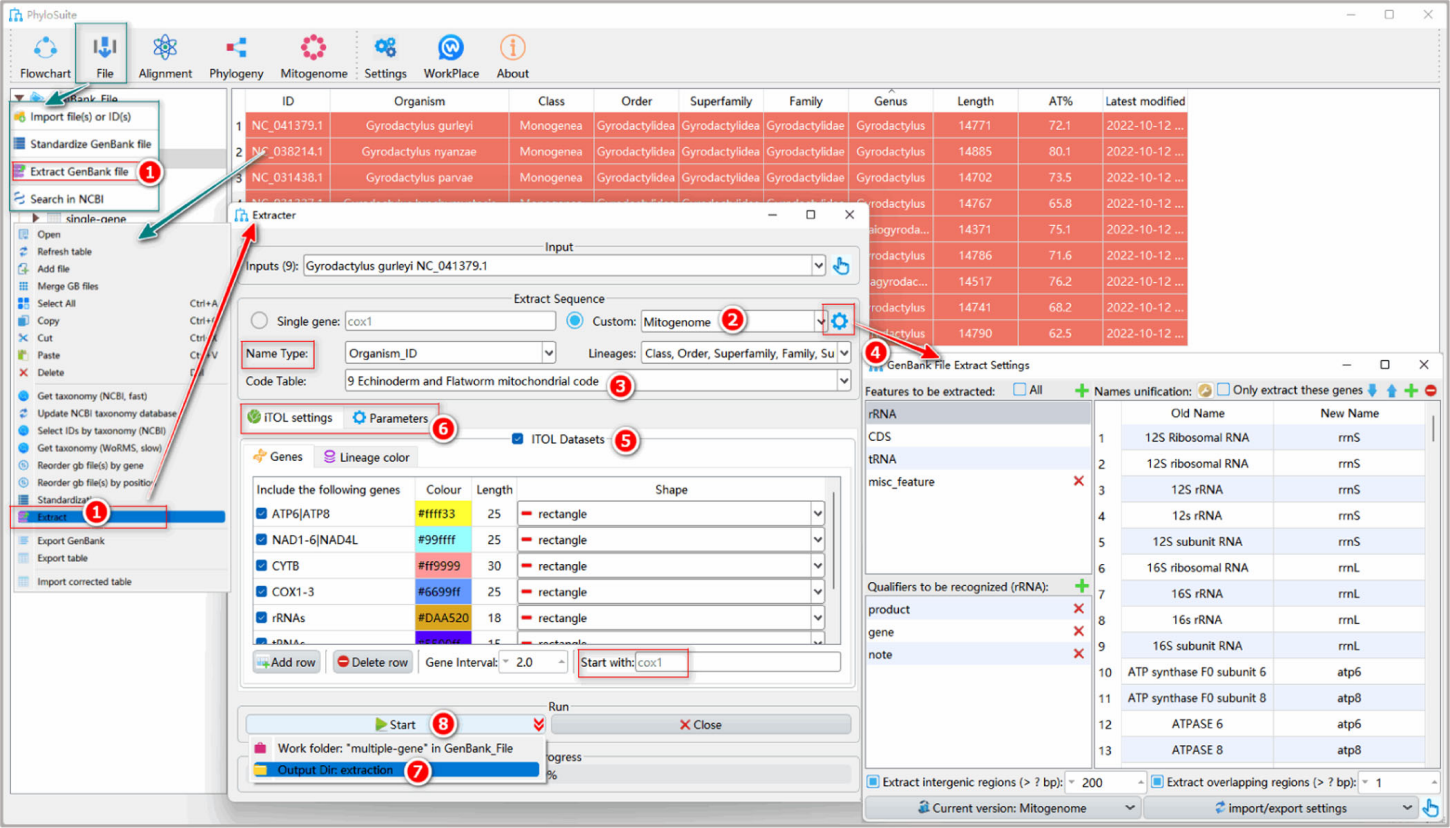
Extracting genes from mitochondrial genomes.

The standard animal mitogenome commonly contains 12–13 PCGs, 22 tRNA genes, and 2 rRNA genes. To use them for downstream analyses, we need to extract them first. The extraction function can also be applied to other types of molecular data, such as single genes, chloroplast genomes, plasmid genomes, bacterial genomes, viral genomes, and etc. However, users should make sure that their data are downloaded in the “GenBank” format. The extraction procedure is shown in Figure 7.

1.4.1 Press Ctrl+A to select all sequences, right‐click and then select the “Extract” function (or access it via the “File” –”Extract GenBank file“ drop‐down menu). An “Extracter” window will pop up.

1.4.2 Select the correct extraction mode from the “Custom” drop‐down menu. Here we selected “Mitogenome.”

1.4.3 Code Table: select the correct codon table that matches your data in the drop‐down menu of “Code Table.” In this tutorial, the 9th codon table (The Echinoderm and Flatworm Mitochondrial Code) was selected for Gyrodactylidea to correctly identify stop codons and translate nucleotide sequences.

1.4.4 The extraction function uses gene names to identify homologous genes. However, often, the same gene will be annotated under different names in your dataset. For example, *COX1*, *COI*, and *COXI* are commonly used names for the same mitochondrial gene. Therefore, we need to uniformise gene names. Click the gear‐shaped button to the right of the “Custom” option in the main “Extracter” window. A setting window will pop up. All names in the “Old Name” column will be replaced with the corresponding name in the “New Name” column during the extraction. By default, PhyloSuite includes some common gene aliases. If you want to add more synonyms, you can either add a new row by clicking the “+” button above or importing a curated table by clicking the “Import” button. A good way to start if you have a dataset with many gene names not included in the default settings is to first use the “general” mode to extract data, then go to the “StatFiles” folder and open the “name_for_unification.tsv” file. After you correct all gene aliases in the “New Name” column, import this file to the setting window using the “Import” button. For a detailed tutorial on how to customize the extraction, please access http://phylosuite.jushengwu.com/dongzhang0725.github.io/PhyloSuite-demo/customize_extraction/.

Other settings in the “Extract Sequence” group box:

a. Name Type: customize the sequence name here.
b. Lineages: use the drop‐down menu to select the desired taxonomic group that you wish to display in the results (statistics tables, iTOL files, etc.).

All of the above steps are shown in Figure 7.

**Figure 8:**
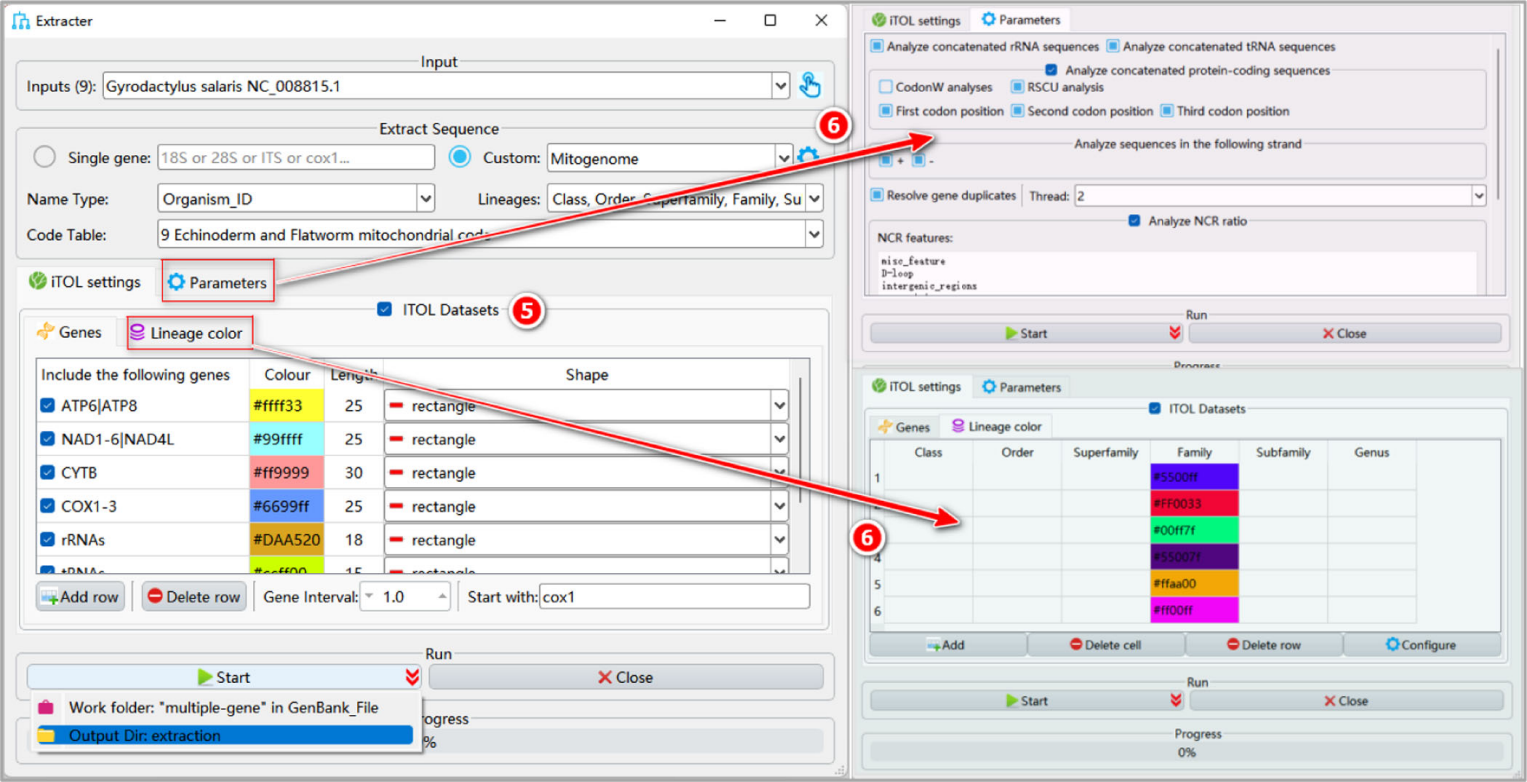
General settings and parameters for extraction.

1.4.5 iTOL annotation parameter settings used for annotation of phylogenetic trees.

A detailed introduction of various parameters for iTOL and other options is shown in Figure 8.

a. Genes tab: we can set the color, length, and shape of genes that we wish to visualize in iTOL. We can specify the starting gene for the gene order display via the “Start with” input box.
b. Lineage color tab: double‐click on the table cell to select a color for the taxonomic group. If the number of colors that you set is less than the number of extracted taxonomic groups, random colors will be assigned to the remaining taxa. For example, if there are 10 classes, and we only set 5 colors, the remaining 5 classes will use random colors.

1.4.6 Parameters tab: select the analyses you wish to conduct.

1.4.7 Users may specify the name of the output folder (here we named it “extraction”) by clicking the arrow on the right of the “Start” button and clicking the row of the “Output Dir” to set a new name (we suggest to do this for every analysis, so will not mention it henceforth).

1.4.8 Finally, click the “Start” button to start extracting.

### Multiple sequence alignment

#### What is sequence alignment?

**Figure 9:**
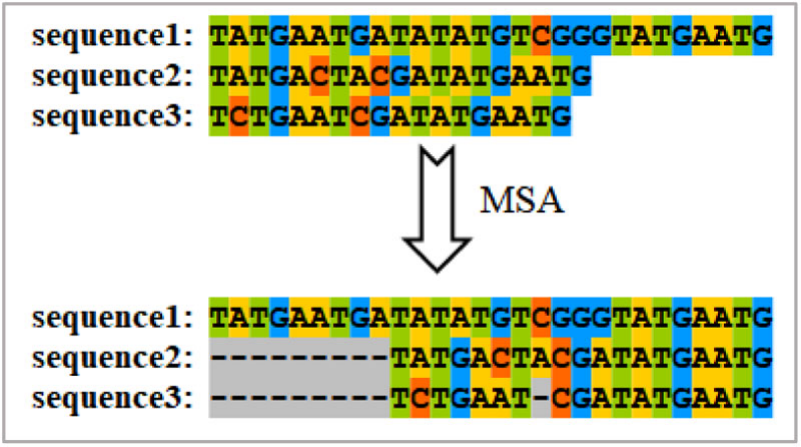
Illustration of a multiple sequence alignment. Gaps are represented by “‐”.

Multiple sequence alignment (MSA) is a procedure that aims to infer homology among characters. Most commonly, this process introduces gaps into sequences to produce a character matrix where all sequences are of equal length and all homologous characters are aligned [15, 16]. These gaps are commonly indicated by “‐” (Figure 9). As a result, the procedure can infer homologous regions of biological sequences that harbor evolutionary events between molecules by determining the positions of matching bases, substitutions, and insertions (or deletions) between sequence sites [17].

**Box 1: Why conduct an MSA?**

Establishing site homology relationships is a prerequisite for all phylogenetic inference methods as improperly aligned sequences will negatively affect the accuracy of downstream evolutionary analyses, such as phylogenetics, homology modeling, database searches, motif finding, genome annotations, etc. Therefore, MSA is a key step that should be conducted with maximum precision [18].

**Why use MAFFT to make MSA?**

There are two important criteria to consider when choosing software to conduct MSA: accuracy and speed. MAFFT employs a simplified scoring system, which allows reduced CPU time, increased alignment accuracy of sequences with large insertions, extensions and deletions, and alignment of distantly related sequences of similar length [19]. A comparison of nine popular MSA programs (Clustalw, Clustal Omega, DIAIGN‐TX, MAFFT, MUSCLE, Poa, Probalign, Probcons and T‐coffee) showed that MAFFT performs well in terms of accuracy and speed, especially for relatively large datasets [20].

#### How to use MAFFT in PhyloSuite?

1.5.1 Right‐click the results folder of “Extraction”, and then select “Import to MAFFT.”

**Figure 10:**
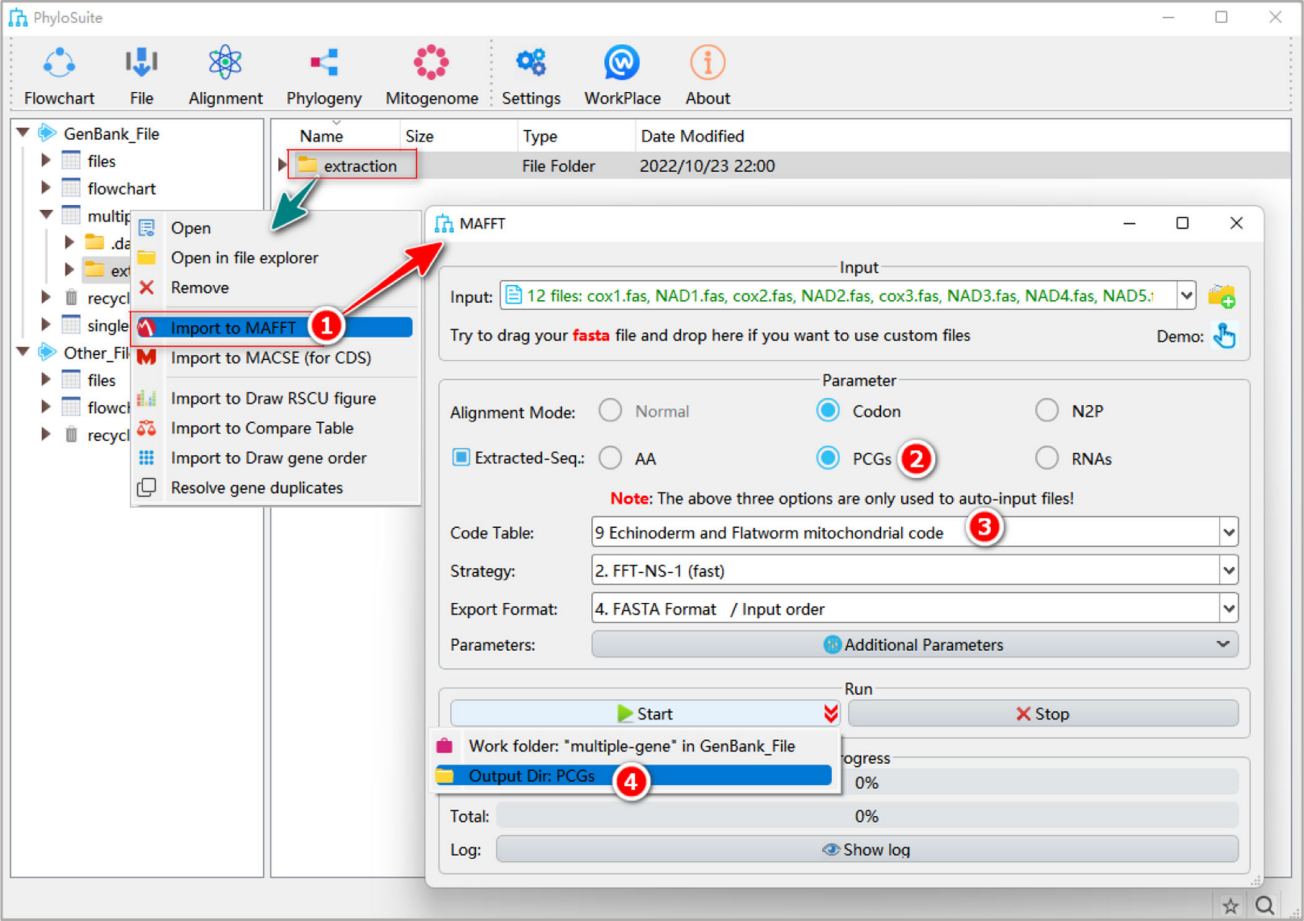
Multiple sequence alignment using MAFFT.

1.5.2 The “Extracted‐Seq” check box will be automatically checked. Three functions can be used: AA, PCGs, and RNAs. Checking “PCGs” will import extracted nucleotide sequences of PCGs (in combination with the “Codon” alignment mode), checking “AA” will import extracted amino acid sequences of PCGs (in combination with the “Normal” mode), and checking “RNAs” will import the extracted sequence of all tRNA and rRNA genes (in combination with the “Normal” mode). Note that these options are available only when importing the files extracted by PhyloSuite into MAFFT. The “Codon” mode was designed by us, especially for PhyloSuite, so this mode is not available in the default MAFFT software. The procedure is: the nucleotide sequences are translated into amino acid sequences, aligned by MAFFT, and then translated back into nucleotides, thus making sure that the codon frame is maintained.

1.5.3 Select the 9th code table for Gyrodactylidea. Note that the code table option only works for “PCGs.”

1.5.4 Set the results folder name (here we named it “PCGs”, “AA,” and “RNAs”) and click the “Start” button to start the multiple sequence alignment (Figure 10).

#### How to align sequences that were not extracted by PhyloSuite (i.e., sequences that are not in the PhyloSuite workplace)?

**Figure 11:**
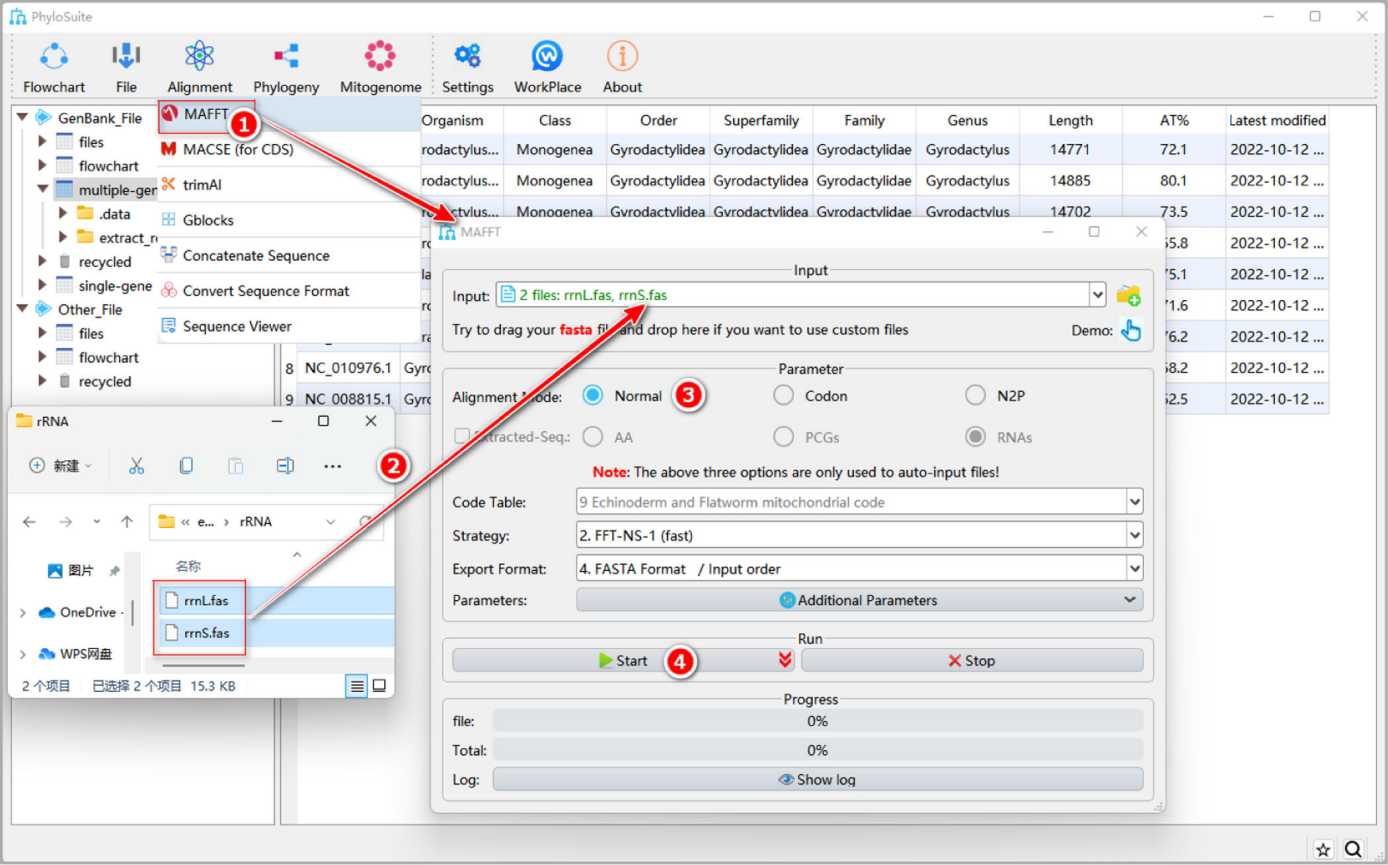
Aligning customized sequences in MAFFT.

Drag the prepared files in the “Fasta” format and drop them into the “Input” combo‐box. Select the option in the “Alignment Mode” that suits your data. Note that in this case, the options in the “Extracted‐Seq” will be disabled. Other steps are the same as in 1.5.2–1.5.4 (Figure 11).

Other parameters of MAFFT can be set according to your own needs. For a comprehensive manual of MAFFT, please visit https://mafft.cbrc.jp/alignment/software/manual/manual.html.

### Codon alignment optimization using MACSE (optional)

**Box 2: Why optimize the codon alignment using MACSE?**

The algorithm of codon alignment implemented in PhyloSuite is similar to the one in MEGA [21] and TranslatorX [22]. These algorithms may produce errors when there are premature stop codons and frameshift mutations present in the alignment [5]. MACSE implements an improved classical “Needleman‐Wunsch” algorithm [23, 24], designed to tackle this problem. It estimates where (along the gene) and when (along the phylogeny) pseudogenization events have occurred [25], thus allowing alignment of PCGs and pseudogenes while maintaining the (putative) ancestral codon structure [26].

**Figure 12:**
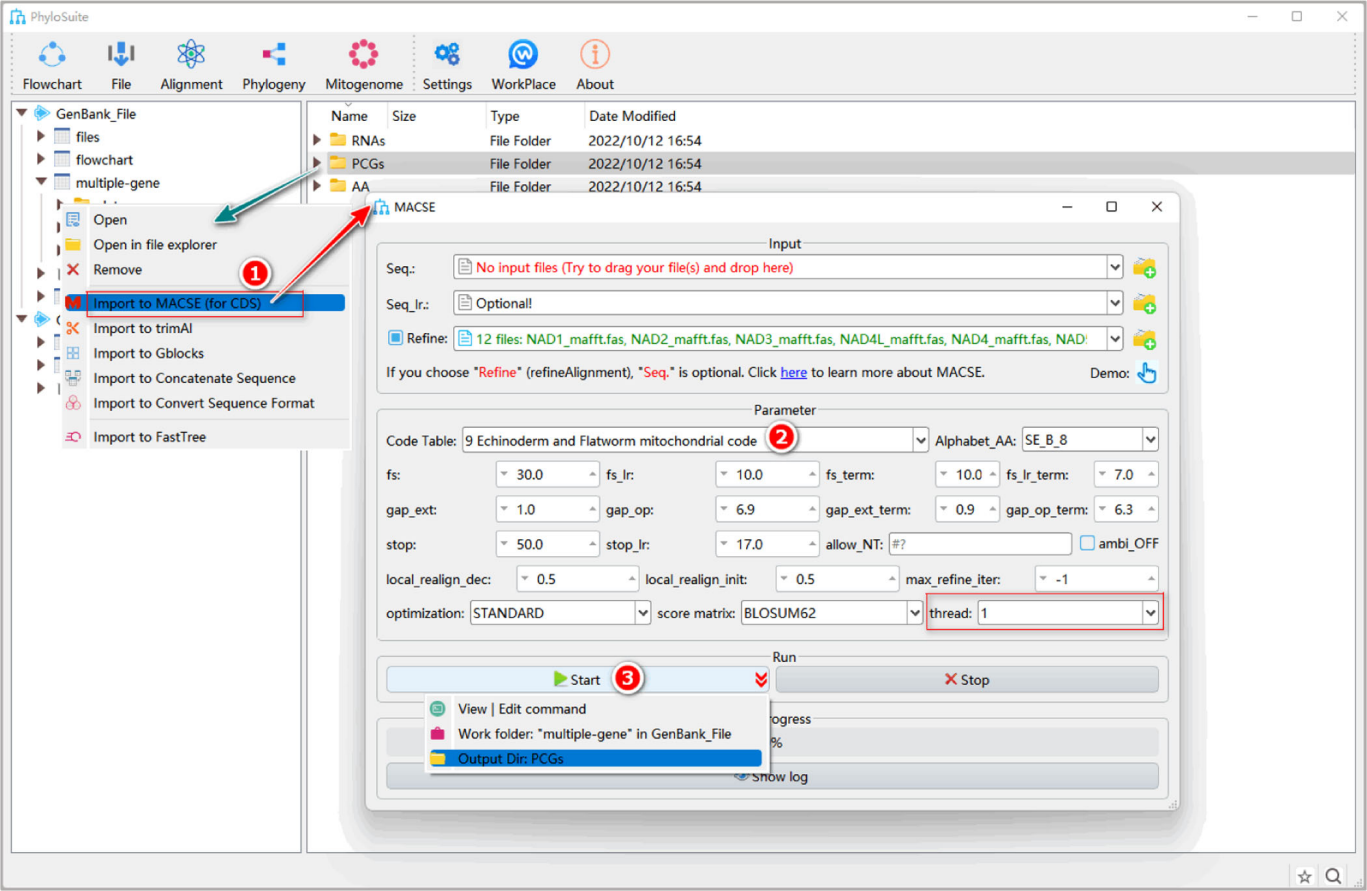
Optimization of multiple codon sequence alignments using MACSE.

As suggested in the MACSE documentation, it is best to first align codon sequences using other programs (such as MAFFT), and then refine the alignment in MACSE. This is a better strategy for big data because directly aligning codon sequences using MACSE is rather slow, so we will use this strategy in the following steps (Figure 12).

#### How to use MACSE in PhyloSuite?

1.6.1 Right‐click the results folder of “MAFFT”, then select “Import to MACSE (for CDS)”. The “Refine” box will be automatically checked, and the MAFFT alignment results will be automatically imported.

1.6.2 Select the 9th code table for Gyrodactylidea.

1.6.3 Set the results folder name (here we named it “PCGs”) and click the “Start” button to start the refinement (Figure 12).

Note that MACSE will mark detected frameshift mutations with “!” or “*” symbols in the result file. As these symbols are likely to affect downstream analyses, in addition to the original output file of MACSE (with “_NT” and “_AA” in the file name), PhyloSuite additionally generates files (with “removed_chars” in the file name). In these files, the above‐mentioned special characters are replaced with “?”. These files will be used for downstream analyses.

#### How to use customized sequences (i.e., the input files prepared by the user)?

**Figure 13:**
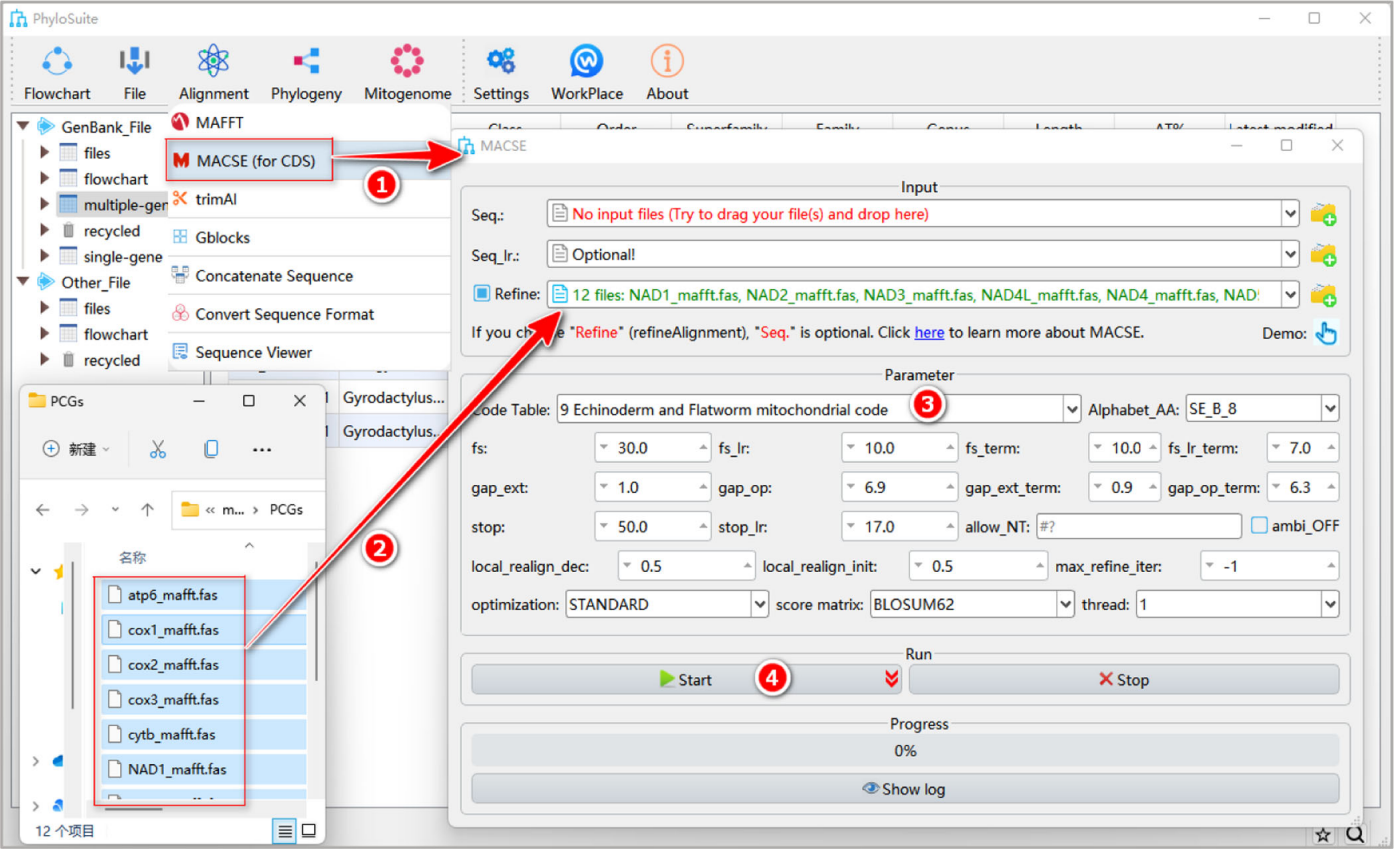
Aligning customized sequences in MACSE.

Select the prepared files in the “Fasta” format and drag them into the “Seq” combo‐box (for unaligned sequences) or the “Refine” combo‐box (for aligned sequences; remember to check the “Refine” checkbox) (Figure 13). The other steps are the same as in 1.6.2–1.6.3.

Note that only PCGs can be aligned using MACSE. Other parameters of MACSE can be set according to your own needs. For a comprehensive manual for MACSE, please visit https://bioweb.supagro.inra.fr/macse/index.php?menu=intro.

### Alignment trimming (optional)

#### What is alignment trimming?

**Figure 14:**
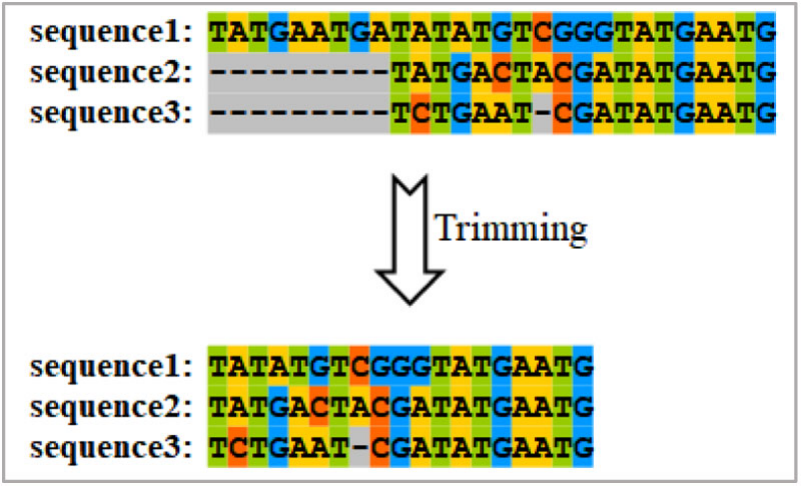
Illustration of multiple sequence alignment trimming.

Alignment trimming is the process that removes poorly aligned sites, which may be caused by falsely inferred site homology, multiple substitutions, or large deletions and insertions in the MSA (Figure 14) [27].

**Box 3: Why trim MSA?**

The MSA quality can strongly influence the accuracy of subsequent phylogenetic analyses [18, 25]. Often, nucleotide and amino acid sequences cannot be perfectly aligned by currently available algorithms. This may produce misaligned sites, which in turn may negatively affect the downstream phylogenetic and other evolutionary analyses. MSA trimming can remove such sites, as well as substitutionally saturated sites, from the alignments. In this way, MSA trimming aims to remove noise and retain signal [28]. In addition, another reason for trimming is that gaps have to be either ignored or treated as a special base or amino acid, depending on the evolutionary model applied [29].

**Why use Gblocks for PCGs alignment trimming?**

HmmCleaner (designed for AA dataset and only available in the Linux version of PhyloSuite) utilizes segment‐filtering methods, which is better than block‐filtering methods (Gblocks and trimAl) in terms of improvement of the quality of evolutionary inference [30, 31, 32]. Regarding the two software programs that use block‐filtering methods, trimAl performs better than Gblocks with large‐scale phylogenomic analyses with thousands of alignments [18, 32]. For PCGs, it is best to use the “Codons” mode in Gblocks, because it can trim sequences while maintaining the codon frame. For RNAs, we can use trimAl; for AA, we can use trimAl or HmmCleaner.

#### How to trim MSA in PhyloSuite?

1.7.1.1 Right‐click the results folder generated by MACSE (for PCGs) or MAFFT (for RNAs and AA), and then select “Import to Gblocks”.

**Figure 15:**
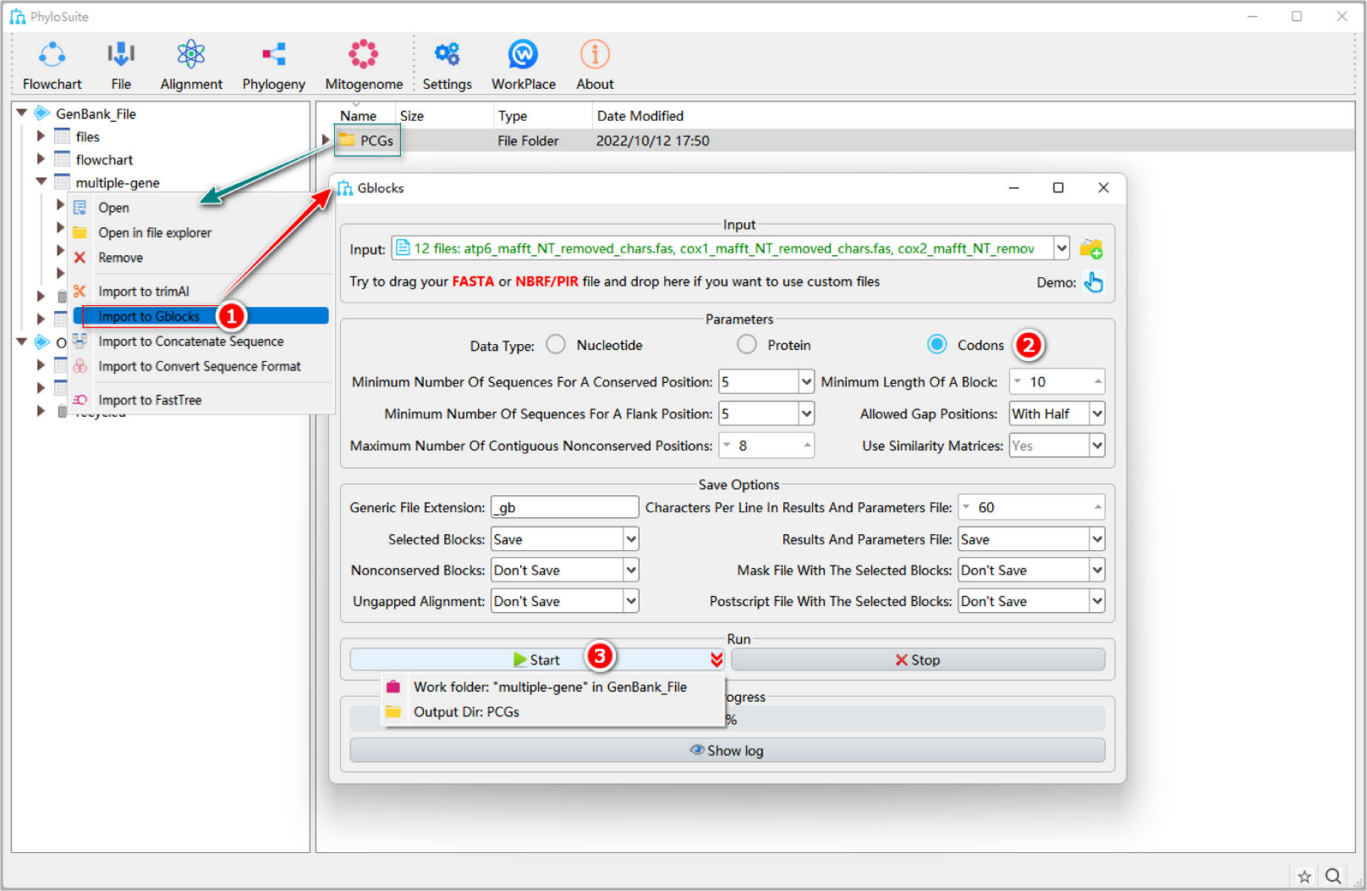
PCGs alignment trimming using Gblocks.

1.7.1.2 Select the suitable “Data Type”: for PCGs select “Codons”, for RNAs select “Nucleotide”, for AA select “Protein.”

1.7.1.3 Set the results folder name (here it is named “PCGs”) and click the “Start” button to start trimming (Figure 15).

#### How to trim RNA sequences?

1.7.2.1 Right‐click the results folder of RNAs generated by MAFFT and then select “Import to trimAl.”

**Figure 16:**
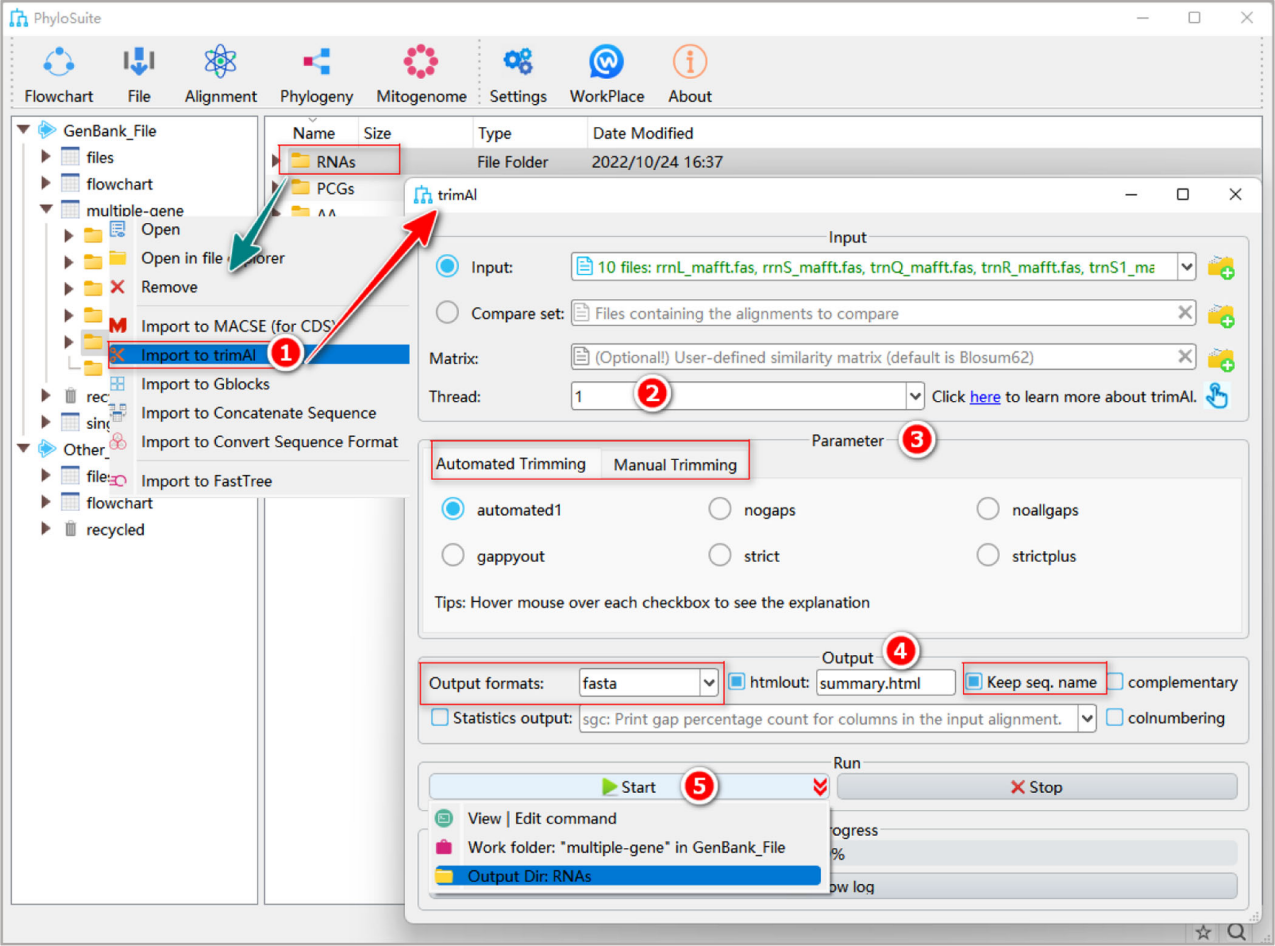
RNA alignment trimming using trimAl.

1.7.2.2 “Threads” parameter can be used to run trimAl analyses for multiple files in parallel.

1.7.2.3 Select a suitable trimming “Parameter.”

a. Manual Trimming: customize trimming parameters, such as gap threshold.
b. Automated Trimming: trimming according to the user‐defined (“nogaps” and “noallgaps”) or MSA features‐based (“gappyout”, “strict,” and “strictplus” methods) thresholds. “automated1” is a heuristic method to select the best automatic methods to trim MSA, which can be used to optimize the Maximum Likelihood tree reconstruction.

Tips: the details for parameters can be seen in the trimAl manual: http://trimAl.cgenomics.org/_media/manual.b.pdf.

1.7.2.4 Set the parameters in “Output.”

a. “Output formats”: set the output format.
b. “Keep seq. name”: avoid trimAl trimming the sequence name.

1.7.2.5 Set the results folder name (here we named it “RNAs”) and click the “Start” button to start trimming (Figure 16).

#### How to use customized sequences (i.e., files prepared by yourself)?

**Figure 17:**
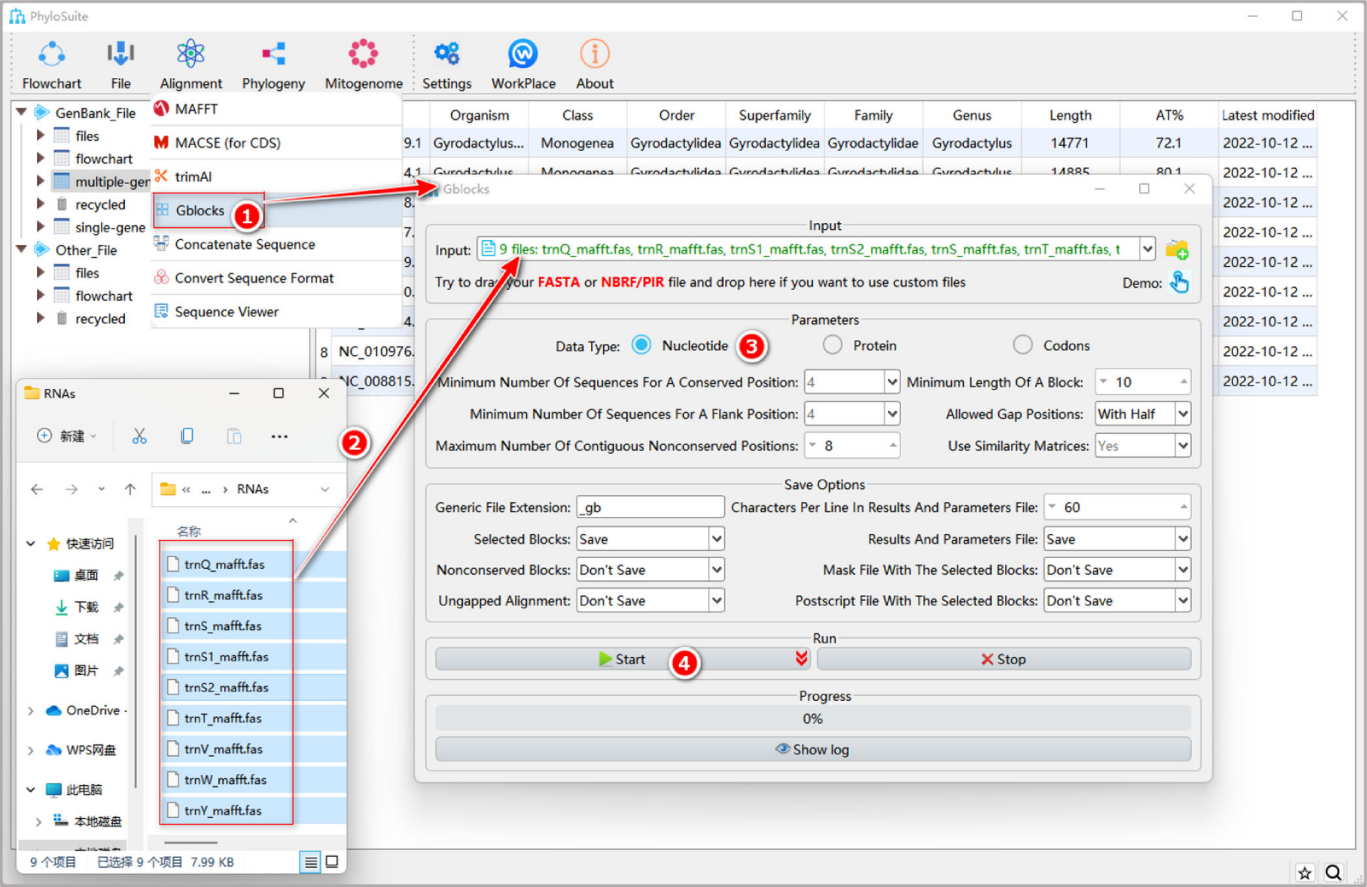
Trimming customized sequences using Gblocks.

Select the prepared alignment of sequences files in “Fasta” format, drag them into the “Input” combo‐box of the Gblocks interface, and then select the proper “Data Type” (Figure 17). The next steps are the same as in 1.7.1.2–1.7.1.3.

Note that not all alignments require trimming and that the benefit/cost balance of trimming remains debated, as MSA trimming software programs may not be perfect, and that trimming “problematic” sites may cause a loss of information that may outweigh the benefits of trimming [33]. Other parameters of Gblocks can be set according to your own needs; for a comprehensive manual of Gblocks, please visit https://molevol.cmima.csic.es/castresana/Gblocks/Gblocks_documentation.html.

### Alignment concatenation

#### What is the concatenation of alignments?

**Figure 18:**
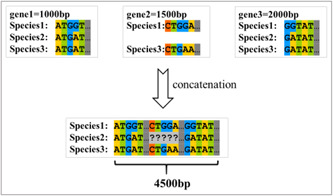
Illustration of sequence concatenation.

For multilocus phylogenetic analyses, we first have to extract and align single loci (genes); following this, they have to be concatenated into a “supermatrix” or “super‐alignment [17]” In this process, individual sequences are matched according to their unique label (e.g., “Species1” in three gene files in Figure 18) and merged into a single sequence. The missing genes are marked as “?”.

**Box 4: Why do we concatenate alignments?**

Concatenated alignments of multiple loci usually carry a larger amount of information than single loci, so they commonly produce more stable topologies with higher bootstrap values [17, 34].

#### How to concatenate alignments in PhyloSuite?

As mentioned in the “DATASET INTRODUCTION” section, we will concatenate the alignments into three datasets: PCGsRNA, PCGs12RNA, and AA.

**Figure 19:**
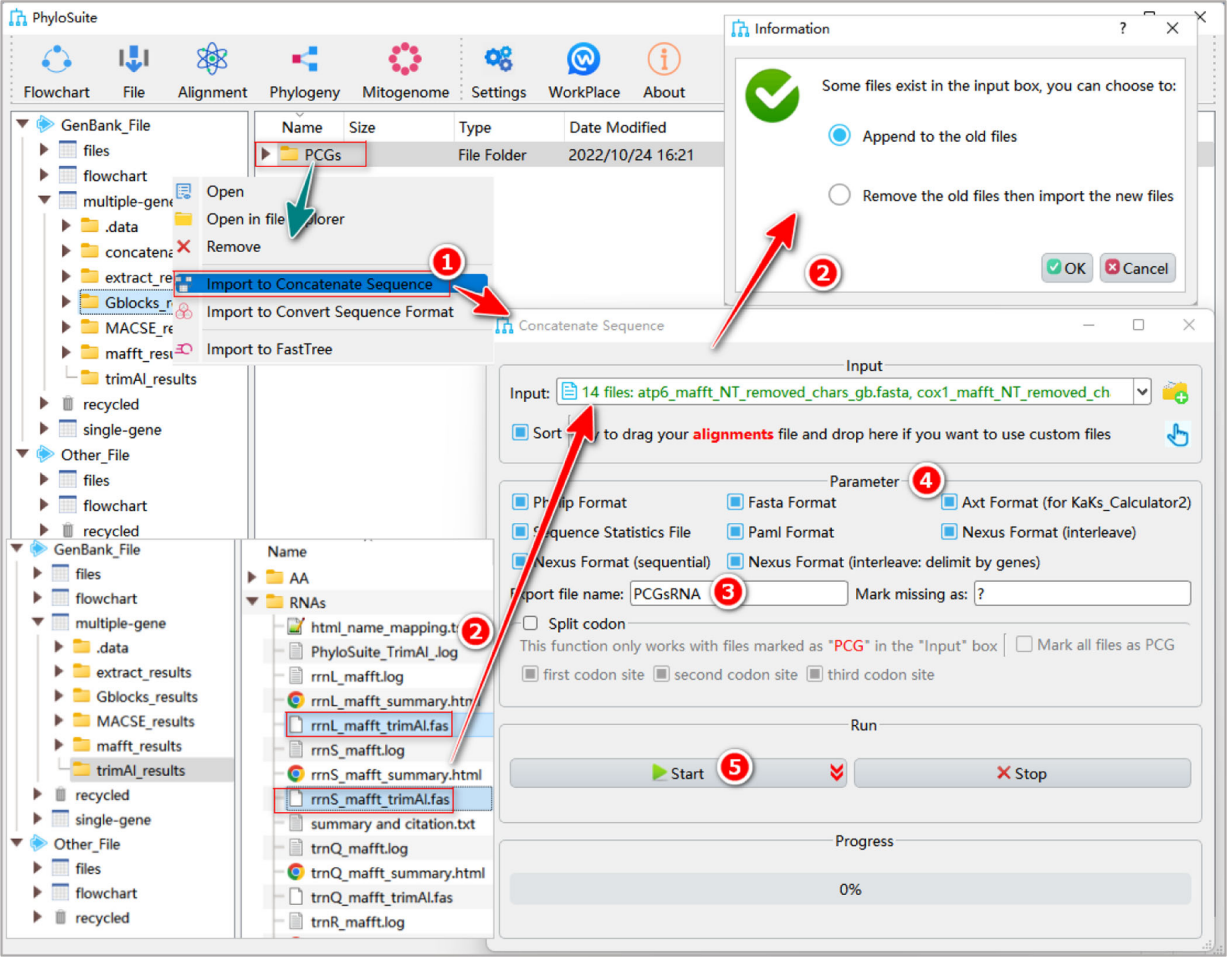
Multiple sequence alignment concatenation of the PCGsRNA dataset.

First, we will show the procedure for the PCGsRNA dataset (Figure 19).

1.8.1 Right‐click the PCGs results folder of “Gblocks,” and select “Import to Concatenate Sequences”. The trimmed PCGs alignments will be automatically imported into the “Input” combo‐box.

1.8.2 Open the RNA results folder of “trimAl” in the PhyloSuite display area, select the trimmed sequence files (with “*_trimAl.fas” in the name) and drag them to the “Input” box of the “Concatenation” panel. Choose “Append to old files” to append the RNA files to previously imported PCGs files (otherwise the previous batch of sequences will be replaced by the new ones).

1.8.3 In the “Export file name” option, you can set the name of the output files.

1.8.4 Select the output formats available in the “Parameter” group‐box.

1.8.5 Set the results folder name (here we named it “PCGsRNA”) and click the “Start” button.

#### How to select a subset of codon sites?

**Figure 20:**
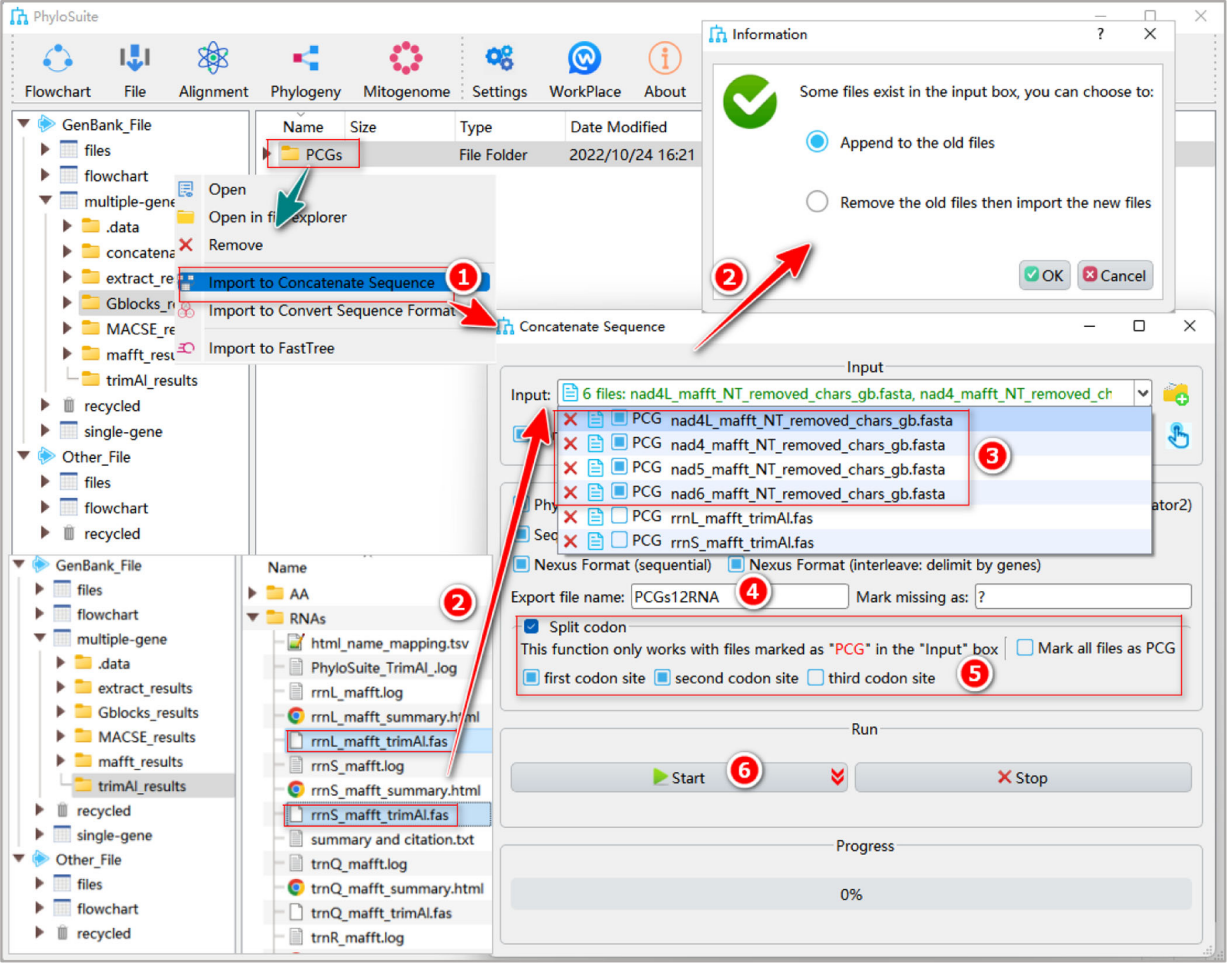
Multiple sequence alignment concatenation of the PCGs12RNA dataset.

In datasets comprising distantly‐related lineages, the comparatively rapidly evolving third codon sites of PCGs often display substitution saturation, which may hinder phylogenetic reconstruction [35]. In such cases, it is better to remove the third codon site from the alignment used for phylogenetic analyses. We added such a function to PhyloSuite (Figure 20).

The input step is the same as described in sections 1.8.1 and 1.8.2. After that, we need to open the drop‐down list by clicking the “Input” combo‐box, and check the “PCG” checkbox if the file contains protein‐coding genes. If you have many PCG files, to save time you can also use the “Mark all files as PCG” checkbox to check all files. Note that in Figure 20 we only displayed six files for a better presentation. Then, check the “Split codon” checkbox, and check both the “first codon site” and “second codon site” checkboxes (but uncheck the “third codon site” box). Other operations are similar to the steps described in 1.8.2–1.8.5 (note that here we named the output files and folder “PCGs12RNA”).

#### How to concatenate the AA dataset?

**Figure 21:**
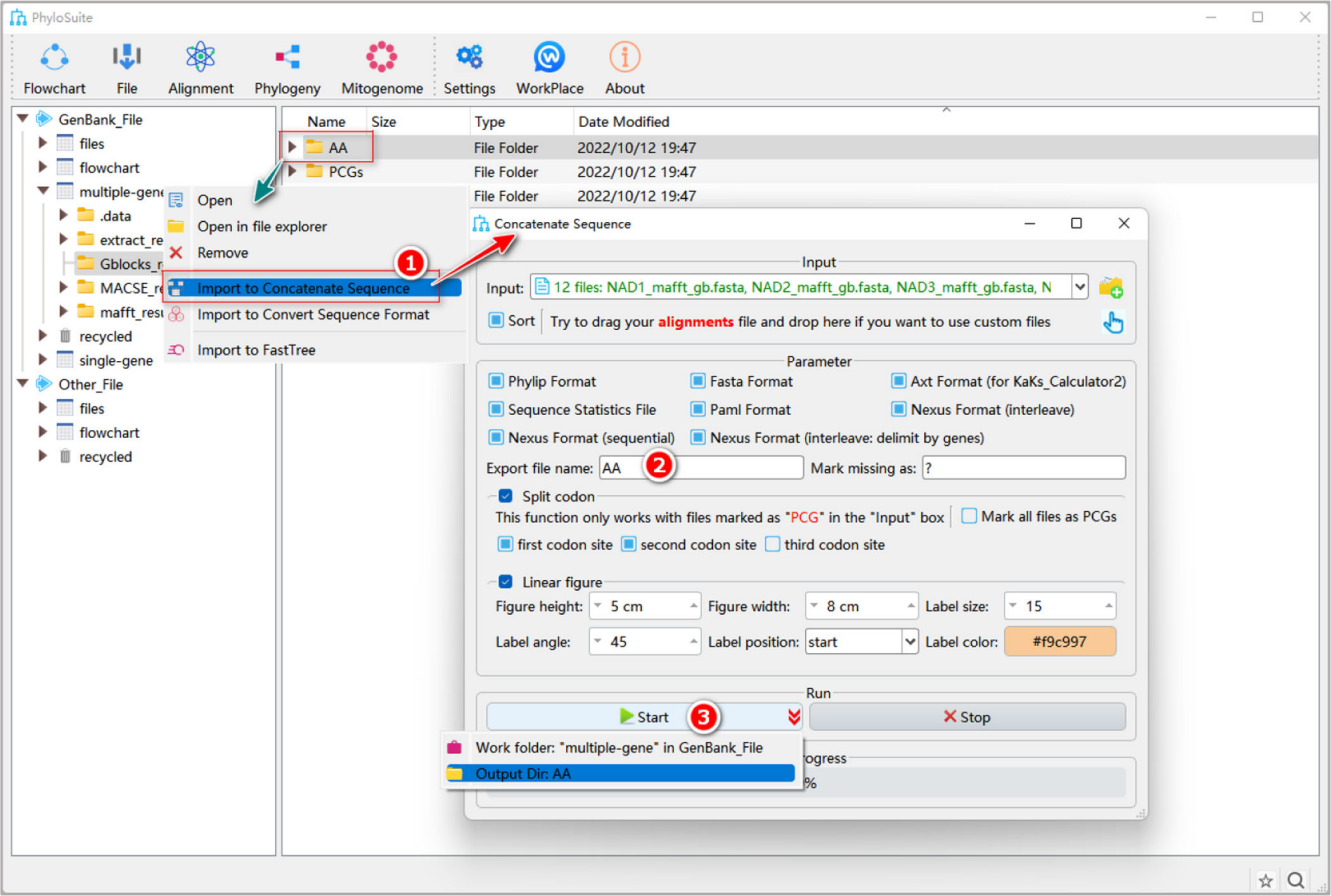
Multiple sequence alignment concatenation of AA.

Right‐click the AA results folder of “Gblocks” or “trimAl”, and select “Import to Concatenate Sequences” (Figure 21). The other steps are the same as in sections 1.8.3–1.8.5.

#### What about using customized sequences (i.e., files prepared by yourself)?

**Figure 22:**
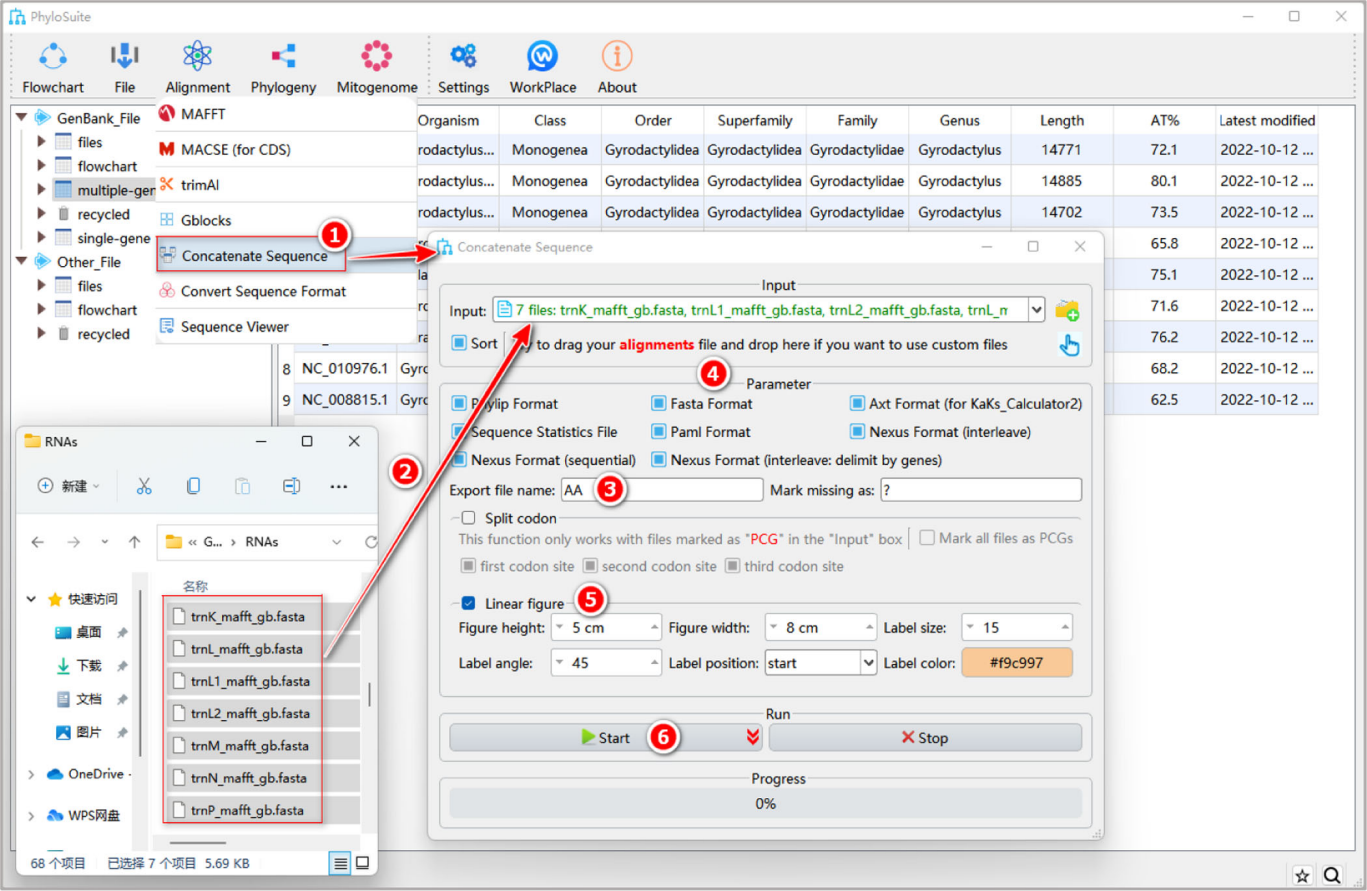
Multiple sequence alignment concatenation of customized sequences.

Select the prepared alignment files (in “Fasta”, “Phylip,” or “Nexus” formats) and drag them into the “Input” combo‐box (Figure 22). Subsequent steps are the same as in 1.8.3–1.8.5.

### Optimal partitioning strategy and model selection

For beginners who may wish to select the model for the entire sequence matrix (non‐partition mode), please refer to section 1.4 “The optimal model selection” in supplementary file and then go directly to section “Maximum likelihood (ML) phylogenetic tree reconstruction.”

#### What is partitioning?

Partitioning allows us to independently estimate evolutionary models for different sets of sites (partitions) in an MSA [36]. For example, for PCGsRNA, we can treat 12 PCGs and 2 rRNA genes as separate partitions, then select the best‐fit model for each of them, and finally determine the optimal partitioning strategy (see below).

**Box 5: Why partitioning?**

In multilocus datasets, different loci (gene or codon sites) may evolve at different rates and patterns, as they often have different biological functions and evolve under different selection pressures [37, 38]. Applying a single model (i.e. assuming that all loci evolved in the same way) for such datasets may seriously mislead the phylogenetic reconstruction [39]. One of the strategies to address this problem is selecting the best‐fit partitioning strategy (i.e., combining genes or sites evolving in a similar way into a subset) and inferring the best‐fit evolutionary model for each subset separately [36]. Note: the details for model selection are elaborated in the single‐gene phylogeny section in the supplementary file.

#### How to partition data in PhyloSuite?

**Figure 23:**
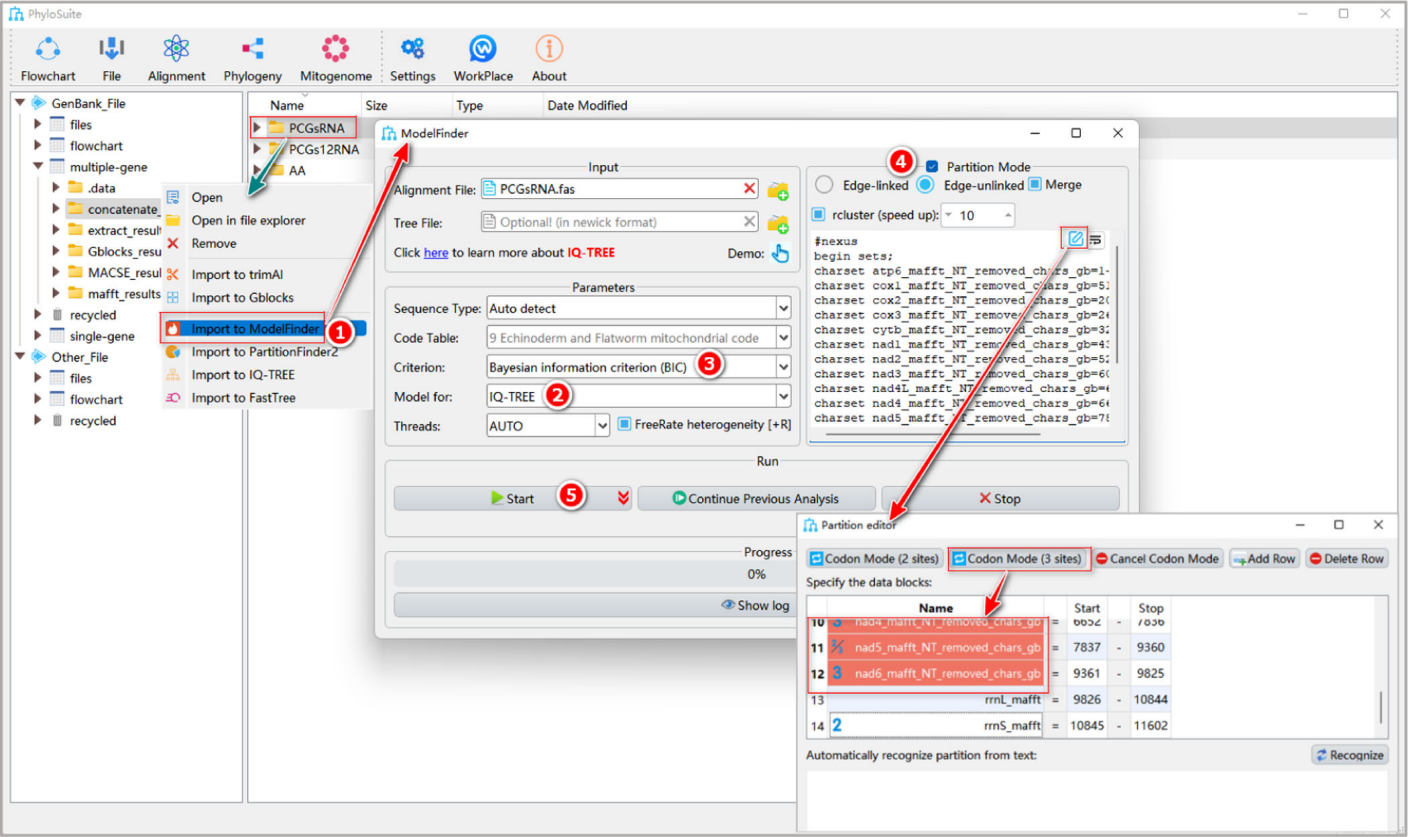
Partitioning analysis using ModelFinder.

First, we will introduce how to use ModelFinder to select the optimal partitioning strategy and best‐fit models for the PCGsRNA dataset (Figure 23).

1.9.1.1 Select the PCGsRNA results folder of “Concatenation,” right‐click and select “Import to ModelFinder.” The alignment and the partition details will be automatically imported into the “Input” box and “Partition Mode” group‐box, respectively.

1.9.1.2 “Model for”: select the downstream software that will be used for phylogenetic tree reconstruction.

1.9.1.3 “Criterion”: it is used to judge the fitness of the model. The default is BIC (Bayesian Information Criterion), where smaller values correspond to better‐fit models [40].

1.9.1.4 “Partition Mode”:

a. Edge‐linked/unlinked: when “Edge‐linked” is selected, partitions will be allowed to have their own evolutionary rates, but an identical set of branch lengths, so MrBayes analysis will produce a single tree for all partitions. This is the option that most users will use most of the time. However, if users wish to test for heterotachy, they may select the (slower and parameter‐rich) “Edge‐unlinked” option, which allows each partition to have its own set of branch lengths [41]. This will result in multiple final trees (one tree per partition) in MrBayes analysis.
b. Merge: this option allows users to find the best‐fit partitioning scheme, that is, merge partitions that evolved under similar evolutionary circumstances (similar rates and types of substitution). This can reduce over‐parameterization and increase the model fit [36, 42, 43, 44].
c. rcluster: specify the percentage of partition schemes analyzed by the relaxed clustering algorithm to reduce computational burden and speed up the analysis [45]. For example, the value of 10 means that only the top 10% of partition schemes are considered (from the IQ‐TREE manual).

**Box 6: How to edit partition data block**:

Click the pencil button on the top right corner of the box to edit the partitions. Select all PCGs partitions and click the “Codon Mode (3 sites)” button on the top. The partition(s) will be changed to the codon mode, in which icons 1, 2, and 3 correspond to partitions comprising the first, second and third codon positions of PCGs, respectively. Note that you should not use the “Codon Mode” function for non‐PCGs data.

1.9.1.5 Set the results folder name (here we named it “PCGsRNA”) and click the “Start” button to start data partitioning and optimal model selection.

Tip: in the results folder, the optimal model partitioning scheme and best‐fit models will be available in a file named “*.best_scheme.nex” as well as a table file named “best_scheme_and_models.csv.”

#### The PCGs12RNA dataset

Right‐click the PCGs12RNA results folder of “Concatenation” and select “Import to ModelFinder.” The other steps are mostly the same as described in 1.9.1.2–1.9.1.6. The only difference is that, in the partition editing step (Box 6), you should use the “Codon Mode (2 sites)” button instead of the “Codon Mode (3 sites)” button for PCGs. This will also be demonstrated in the “PartionFinder2” section (1.9.2.7).

#### The AA dataset

Right‐click the AA results folder of “Concatenation,” and select “Import to ModelFinder.” The other steps are mostly the same as in 1.9.1.2–1.9.1.6 (here we named it “AA”), but no partition editing is needed.

#### How to use customized sequences (i.e., files prepared by yourself)?

Drag the prepared alignment file (in “Fasta”, “Phylip”, or “Nexus” formats) into the “Alignment File” combo‐box, and other steps are the same as in 1.9.1.2–1.9.1.5. The only difference is that you have to customize the partition index in the “Partition Mode” data block edit box (also see section 3 of supplementary file “INPUT/OUTPUT FILES INTRODUCTION”).

Other ModelFinder parameters can be set according to your own needs. For a comprehensive manual for ModelFinder, please visit http://www.iqtree.org/doc/.

#### How to use PartitionFinder?

**Figure 24:**
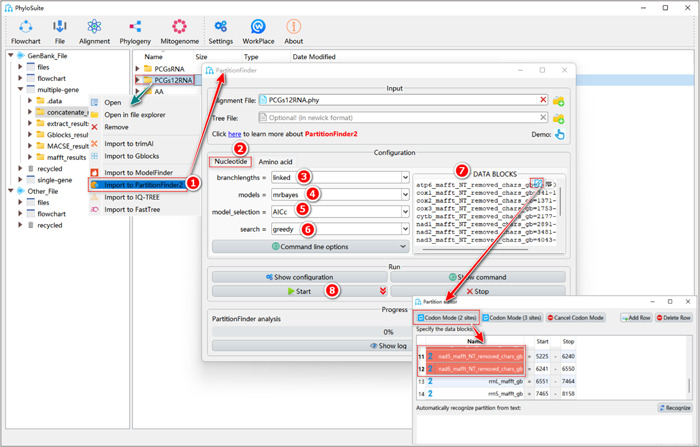
Partitioning and optimal model selection using PartitionFinder2.

Here, we take the PCGs12RNA dataset as an example (Figure 24).

1.9.2.1 File input operation is similar to ModelFinder (see 1.9.1.1), but select “Import to PartitionFinder2.”

1.9.2.2 Select the “Nucleotide” or “Amino Acid” tab according to the sequence type.

1.9.2.3 For the “branchlengths” parameter, please refer to the explanation of “edge‐linked” and “edge‐unlinked” in ModelFinder (1.9.1.4a).

1.9.2.4 “Models” can be selected according to your analysis requirements.

1.9.2.5 The “model_selection” option corresponds to the “Criterion” parameter in ModelFinder, but here the authors of PartitionFinder2 recommend the “AICc” criterion.

1.9.2.6 The “search” option selects the greedy algorithm by default. The greedy algorithm is more efficient than the exhaustive search [36].

1.9.2.7 The operation of “DATA BLOCKS” is the same as the “data block edit box” of “Partition Mode” in ModelFinder (Box 6).

1.9.2.8 Set the results folder name (here we named it “PCGs12RNA”) and click the “Start” button to start data partitioning and optimal model selection.

Tips: the optimal model partitioning scheme can be viewed in the “best_scheme_and_models.csv” file of the results folder. Other parameters of PartitionFinder can be set according to your own needs. For a comprehensive manual of PartitionFinder, please visit https://www.robertlanfear.com/partitionfinder/assets/Manual_v2.1.x.pdf.

#### How to select partition models for different downstream tree inference software programs?

a. IQ‐TREE: select “IQ‐TREE” in the “Model for” combo‐box in ModelFinder. Select “all” in the “models” combo‐box in PartitionFinder2.
b. MrBayes: select “MrBayes” in the “Model for” combo‐box in ModelFinder. Select “mrbayes” in the “models” combo‐box in PartitionFinder2.
c. RAxML: select “RAxML” in the “Model for” combo‐box in ModelFinder. Select “‐‐raxml” in the “Command line options” in PartitionFinder2.
d. BEAST: select “BEAST*” in the “Model for” combo‐box in ModelFinder. Select “beast” in the “models” combo‐box in PartitionFinder2.

### Maximum likelihood (ML) phylogenetic tree reconstruction

#### What is the ML‐based phylogenetic method?

The ML method of phylogenetic reconstruction usually operates on homologous aligned sequences, an evolutionary model, as well as a set of topologies [1, 2]. The likelihood is the probability of observing the dataset based on the given parameters. For each topology, parameters (such as branch lengths, transition/transversion rate ratio, etc.) in the model will be estimated by maximizing the log‐likelihood (*lnL*) [2], and the maximum *lnL* of the topology will be calculated using the estimated parameters. The best maximum likelihood tree is the one with the largest *lnL* among all possible topologies. However, the ML method is very time consuming, especially on large datasets. For example, datasets often produce a large number of possible topologies. Two schemes were developed to save the computation time: (1) a pruning algorithm, which can reduce the repeated calculation for *lnL* [46, 47], and (2) heuristic tree search, such as branch‐swapping algorithms. The latter method first infers a start tree, either randomly or using a faster tree reconstruction method (such as Neighbor‐Joining or Maximum parsimony), then it produces a set of neighboring trees around the start tree, and finally it relies on the optimality criterion to evaluate which tree to retain [2]. In this way, this method avoids the time‐consuming calculation of *lnL* for all topologies [2].

**Box 7: Why use the Maximum Likelihood method for phylogenetic tree reconstruction?**

In comparison with parsimony, distance and ML method with simplistic models, ML reconstruction under optimal and more realistic models (e.g., a model that accommodates variable rates among sites) is less affected by the long‐branch attraction [2, 48, 49], and it can produce consistent results and exhibit better efficiency in recovering the “true” tree [2, 48, 49]. In addition, computer simulation studies showed that ML was more robust (reliable) than distance methods when using highly divergent sequences that violated the assumption of a stationary substitution process [2, 48, 50].

**Why use IQ‐TREE for phylogenetic tree reconstruction?**

In a comparative study of several popular maximum likelihood‐based software programs for phylogenetic reconstruction, including RAxML/ExaML, PhyML, IQ‐TREE, and FastTree, IQ‐TREE showed the highest observed likelihoods for concatenation‐based species tree inference [51].

#### How to use IQ‐TREE in PhyloSuite?

1.10.1 Right‐click the results folder of the PCGsRNA dataset generated in the last step (PartitionFinder2 or ModelFinder), and select “Import to IQ‐TREE”. The alignment will be imported into the “Alignment File” input box. The partitioning results (best‐fit scheme and models) will be imported into the “Partition Mode” input box.

**Figure 25:**
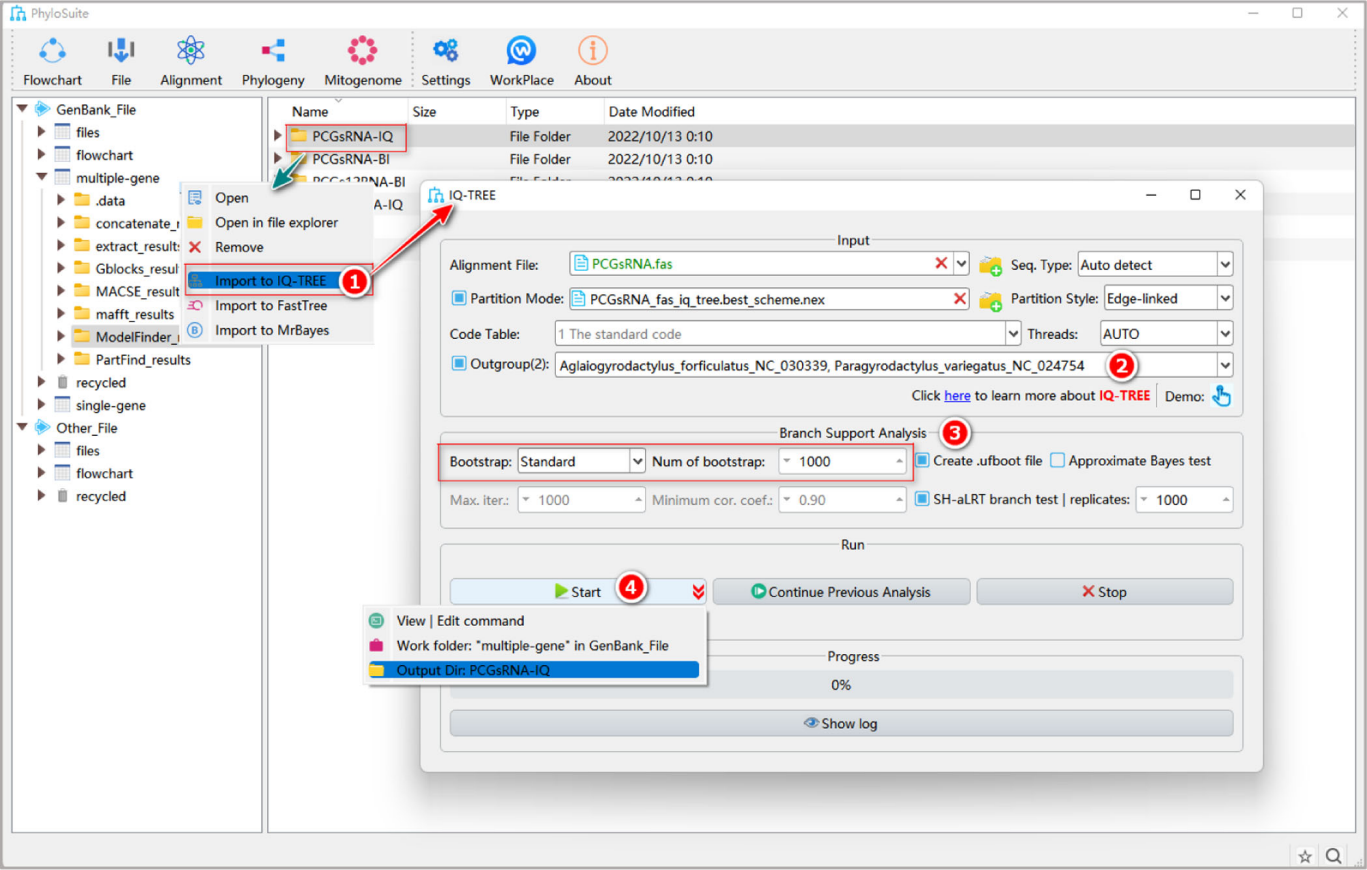
Phylogenetic tree reconstruction using IQ‐TREE software.

Tips: once the “Partition Mode” checkbox is checked, the “Substitution Model Options” group box will be hidden, as the parameters in this box are not used in this mode. If no partitioning results are imported, the best‐fit model in the “Substitution Model Options” will be automatically set according to the results of the last step (see the section “1.4. The optimal model selection”  in supplementary file).

1.10.2 Select outgroups

Outgroup: in phylogenetic analysis, the target group of the study is called the ingroup, and one or more selected groups closest to the ingroup are called the outgroup [17]. It is advisable to select a relatively closely related outgroup, as distantly related outgroups may destabilize the topology of the phylogenetic tree [52].

1.10.3 Set “Branch Support Analysis” parameters. Bootstrap is an algorithm that can evaluate the stability of the phylogenetic tree topology. The higher the value, the more reliable the topology [53].

Note that if you select “Ultrafast” in the “Bootstrap” combo‐box, the “Number of Bootstrap” will automatically change to 5000 (IQ‐TREE manual recommends the number to be > = 1000). If you select “Standard”, the “Number of Bootstrap” will automatically change to 1000 (but you may select a smaller or larger value according to the computational time constraints). The “Ultrafast” model runs faster than the “Standard” model, but recommended thresholds for deeming a branch reliable also differ between the two methods: 95 in the former, and 70 in the latter [54].

1.10.4 Set the results folder name (here we named them “PCGsRNA‐IQ”, “PCGsRNA‐IQ,” and “AA‐IQ”) and click the “Start” button (Figure 25).

Tips: the tree produced by the IQ‐TREE can be found in the results folder as a “*.treefile.”

#### What about using customized sequences (i.e., files prepared by yourself)?

**Figure 26:**
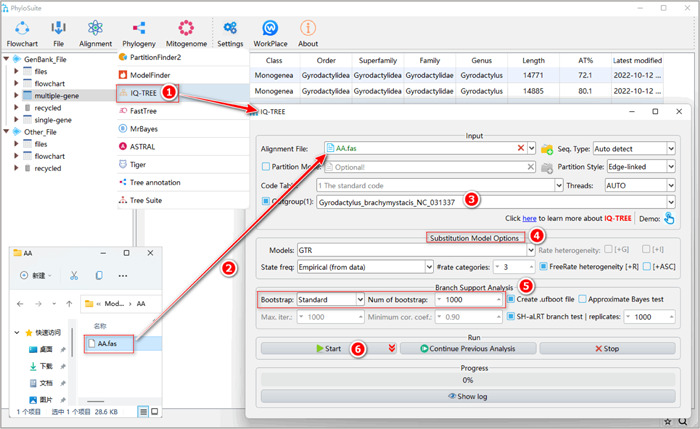
Phylogenetic tree reconstruction using IQ‐TREE and customized input files.

Drag the prepared alignment file (in “Fasta”, “Phylip,” or “Nexus” formats) into the “Alignment File” input box. If you have prepared a partition file, drag it into the “Partition Mode” input box and check it. If there are no partitions, set the best‐fit model for your data in the “Substitution Model Options” (Figure 26). Other parameters and operation steps are similar to those described in 1.10.2–1.10.4.

Other parameters for IQ‐TREE analysis can be set according to your own needs; for a comprehensive IQ‐TREE manual, please visit http://www.iqtree.org/doc/.

### Phylogenetic tree reconstruction based on the Bayesian inference (BI)

#### What is BI in phylogeny?

The Bayesian phylogenetic reconstruction identifies the tree with the highest posterior probability as the best tree [2]. The posterior probability is calculated on the basis of the prior, the data, and the substitution model [2, 55]. Historically, the computation of BI was resource‐heavy [2, 55], but the development of Markov chain Monte Carlo (MCMC) algorithms made it computationally feasible. MCMC is a highly efficient method for simulating posterior distributions [56].

**Box 8: Why use the Bayesian method for phylogenetic tree reconstruction?**

BI is a powerful algorithm for dealing with complex questions in evolutionary biology [56]. It is well‐suited for inferring large trees tractably, detecting natural selection, and selecting the optimal evolutionary models for MSA [56]. Since the first release of MrBayes by Huelsenbeck et al. [56], BI has become a very popular phylogenetic algorithm. Subsequent development of powerful evolutionary models also promoted the popularity of the BI method, so researchers subsequently developed more than 10 BI‐based software programs (see Table 1 in Nascimento et al. [57]).

#### How to reconstruct the BI tree in PhyloSuite?

1.11.1 Right‐click the results folder generated by the last step (PartitionFinder2 or ModelFinder), and select “Import to MrBayes”.

**Figure 27:**
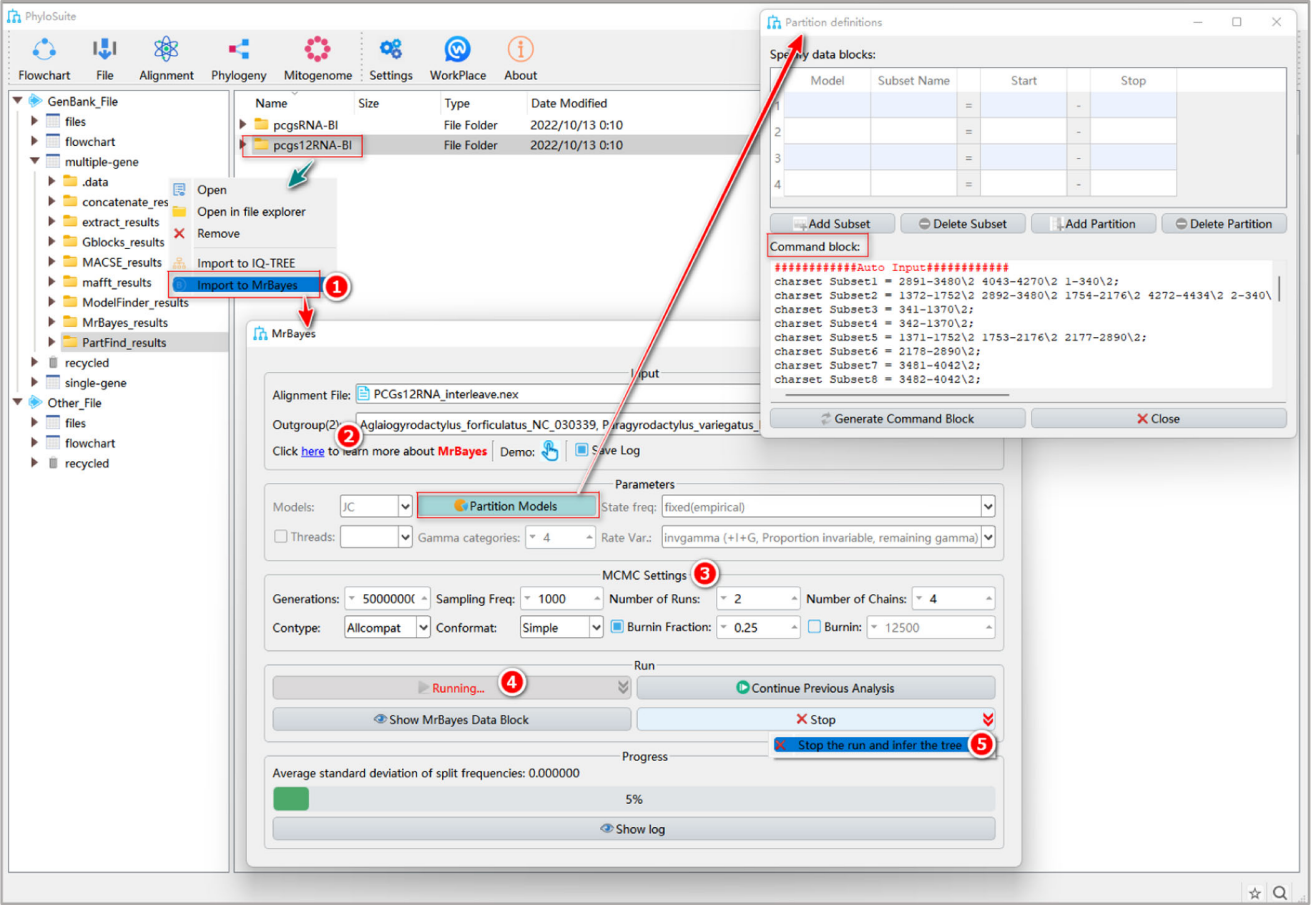
Phylogenetic tree reconstruction using MrBayes in PhyloSuite.

The MSA file will be automatically imported in the “Alignment File” input box along with the optimal partition scheme and the best‐fit models. This can be viewed via double‐clicking the “Partition Models” button (note that if this button is checked, the imported partitioning results will be used for BI tree inference, and settings for some other model parameters, such as “Models”, will be disabled and ignored).

1.11.2 Select outgroups (see 1.10.2 for a detailed explanation).

1.11.3 “MCMC Settings” group box.

a. Generations: this specifies the number of MCMC generations for a single run. Tip: it is better to set a larger value because PhyloSuite can stop the run anytime, whenever the user decides that the run has converged.
b. Sampling Freq: this defines how often (i.e., once in how many generations) the Markov chain is sampled. The optimal value depends on the total number of MCMC generations in the analysis. If you set a small value for this parameter, in long BI runs comprising millions of generations you will get excessively large output files. PhyloSuite v1.2.3 uses 1000 as the default value; in a BI run of 1,000,000 generations, this value will produce 1000 sampling statistics results (each comprising a tree).
c. Nruns: this parameter defines the number of independent analyses that are started simultaneously. The default value is 2.
d. Nchains: the number of MCMC chains running simultaneously. The default value is 4; among these, three are “heated chains” and one is a “cold chain” (for definitions, see MrBayes manual).
e. Contype: the type of consensus tree. “Halfcompat” generates a 50% majority‐rule tree, where clades with Bayesian posterior probability (BPP) values < 0.5 will be treated as polytomies. “Allcompat” adds all compatible groups to the tree, so clades with BPP < 0.5 will be dichotomous. It is similar to the 50% majority rule with “Show frequencies of all observed bipartitions” checked in PAUP. PhyloSuite v1.2.3 uses “Allcompat” by default.
f. Conformat: the format of the consensus tree. “Simple” will generate a simple consensus tree, which can be recognized by a wide range of programs, such as iTOL. “Figtree” will generate richer summary statistics in the result tree file, which can be recognized by the Figtree [58] software. PhyloSuite v1.2.3 uses “Simple” as default.
g. Burnin Fraction: in a BI run, the first subset of trees is usually not as accurate as the trees sampled in the latter part of the analysis. For this reason, a proportion of trees sampled in the early stages of Bayesian analysis is commonly discarded to obtain more stable parameter estimations and reduce simulation errors. This step is called “burnin” [59, 60]. A standard burnin value is 0.25, which corresponds to the removal of the first 25% of samples (also default in PhyloSuite).

Tips: alternatively, you can also directly specify the number of generations that you want to burnin according to the summary statistics results that you get via the “Burnin” option.

1.11.4 Set the results folder name (here we named them “PCGsRNA‐BI”, “PCGs12RNA‐BI,” and “AA‐BI”) and click the “Start” button.

1.11.5 After the BI run converged, pull down the “Stop” menu and select “Stop the run and infer the Tree” (Figure 27).

#### How to evaluate the convergence of a BI run?

As mentioned above, MrBayes uses MCMC to perform Bayesian inference of phylogeny. Good MCMC runs should reach the target posterior probability distribution that is capable of generating a good sample. For example, if there are two runs, their tree samples should be very similar [56, 61, 62].

When MrBayes starts running, the “Progress” group box will show the “Average standard deviation of split frequencies” (ASDSF) value in real time. In general, when the ASDSF value falls below 0.01, it can be used as an indicator that the BI run has convergent [60, 63]. At this point, you can click the arrow to the right of the “Stop” button and select “stop the run and infer the tree” to stop the program and obtain relevant files, including the tree file (see ⑤ in Figure 27).

Additionally, there are other indicators that can be used to evaluate the convergence for MCMC runs. One is the potential scale reduction factor (PSRF) [64], which can be used to compare the differences within and between runs. As runs convergence, the PSRF of all parameters should approach 1.0. The other is the effective sample size (ESS) [63], whose value should be higher than 100; otherwise it is necessary to continue the run. Both indexes can be found in the log file.

#### What to do if the BI run did not converge?

**Figure 28:**
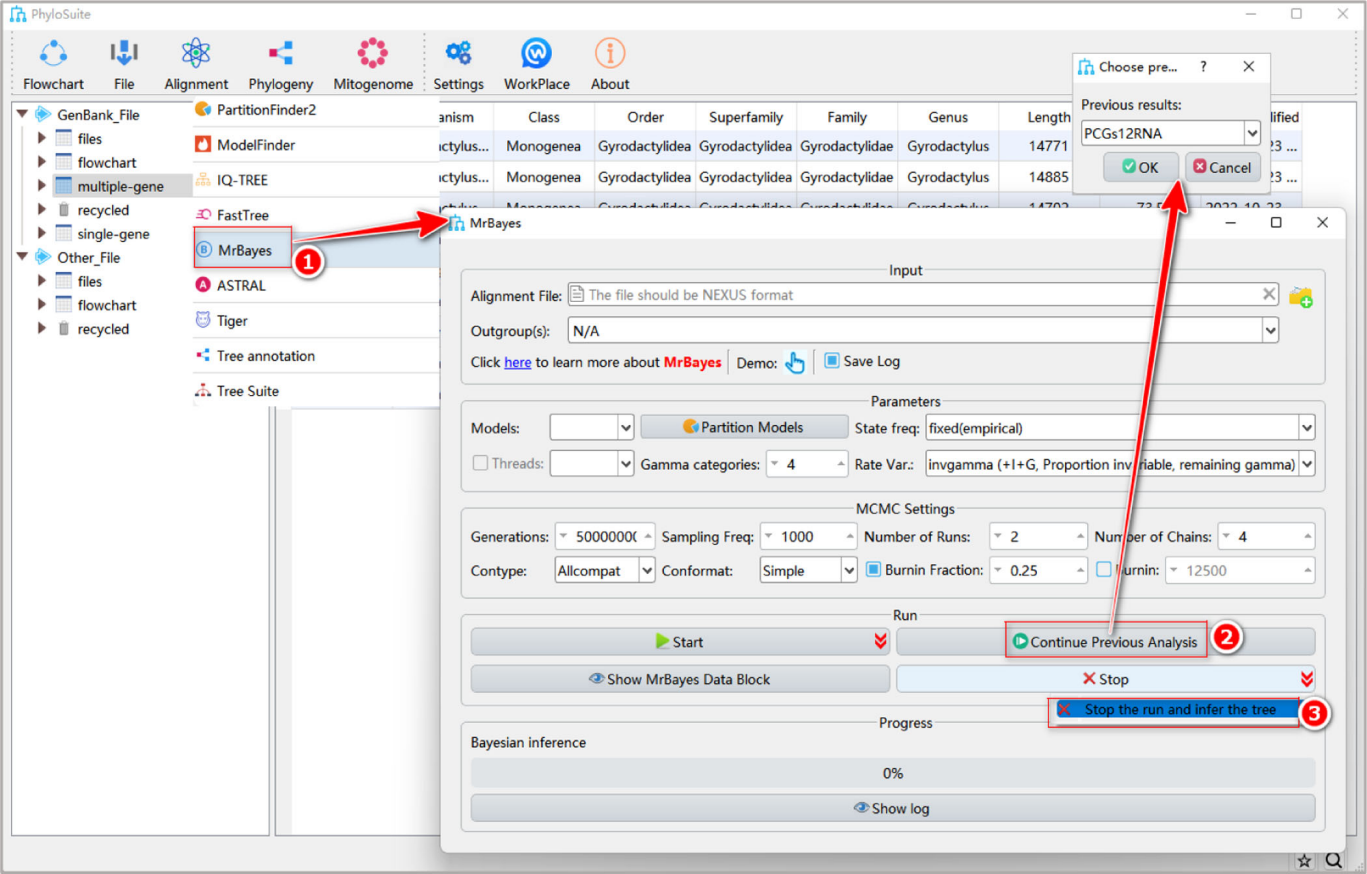
Continuing previous analyses in MrBayes.

If the run did not converge, you may try to continue the analysis. For this, click the “Continue Previous Analysis” button. All MrBayes results (like “PCGs12RNA” in the figure) in the current work folder (“multiple‐gene” in the figure) will pop up in a combo‐box. Select the BI run that did not converge, and click “Ok” to continue the analysis. When you estimate that the run has converged, select “Stop the run and infer the tree” (Figure 28).

#### What about using customized input files (i.e., files prepared by yourself)?

**Figure 29:**
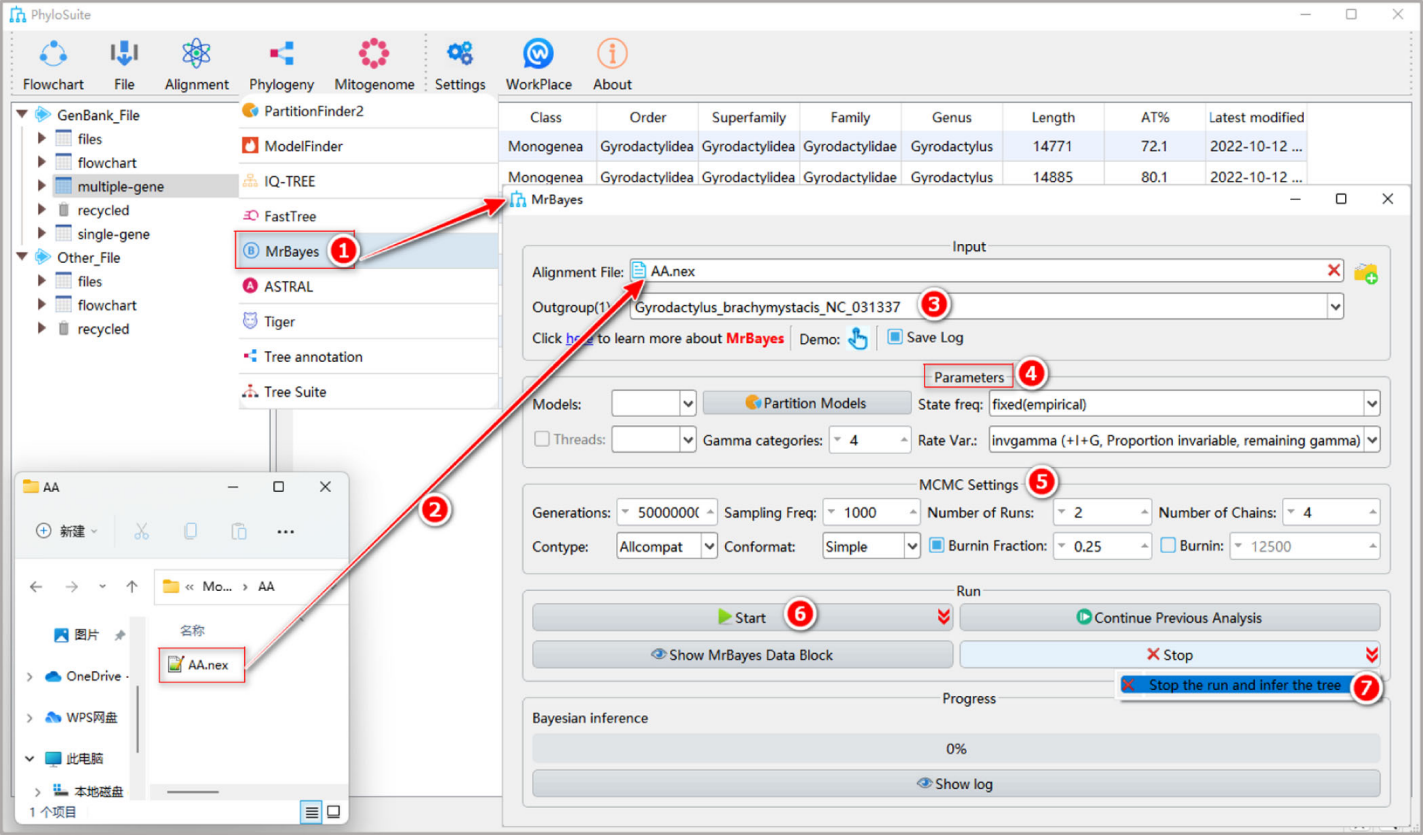
Phylogenetic tree reconstruction using MrBayes and customized input files.

Drag the prepared MSA file into the corresponding input box (the file should be in the “Nexus” format), then set parameters in the PhyloSuite interface, e.g. the best‐fit model (Figure 29). Other steps are similar to the standard analysis (described in 1.11.2–1.11.5).

Other parameters of MrBayes can be set according to your own needs; for a comprehensive manual of MrBayes, please visit https://mrbayes.sourceforge.net/manual.php. To learn how to design a professional Bayesian analysis, please refer to Nascimento et al. [57].

Tips: the tree file of MrBayes is “*.con.tre” in the results folder.

## TREE‐BASED ANALYSES

These analyses are available in the “TreeSuite” function of the “Phylogeny” menu in PhyloSuite.

### The operation procedure of TreeSuite

2.1.1 Right‐click the results folder of “IQ‐TREE” and select “Import to TreeSuite”. The corresponding tree file and the alignment file will be imported into the corresponding input boxes.

**Figure 30:**
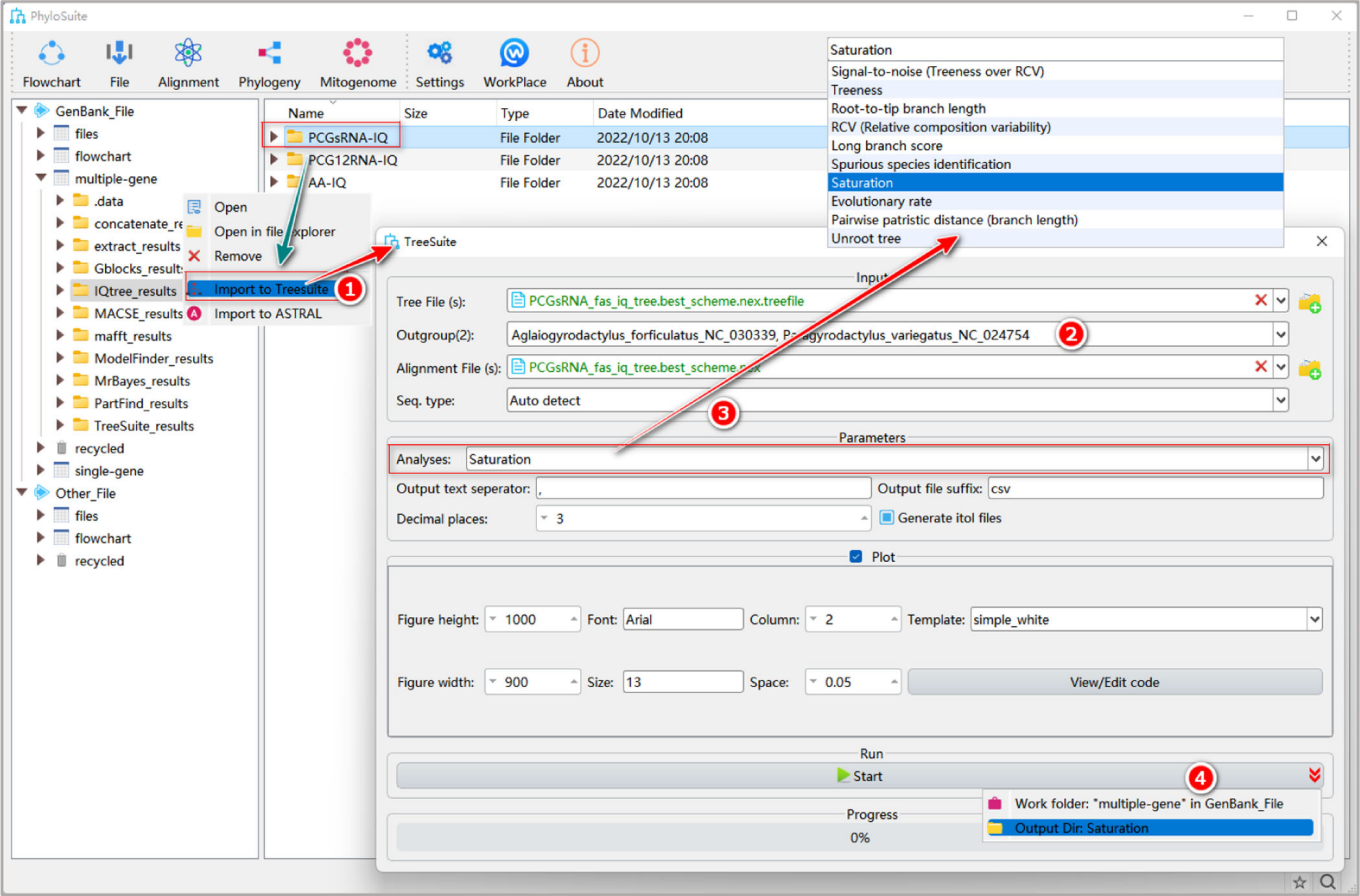
The operation of TreeSuite.

Tips: the input box of tree files allows the standard “Newick”, “Nexus”, “Nexml” and “Phyloxml” formats. The input box of the alignment file accepts the standard “Fasta”, “Nexus” and “Phylip” formats of aligned multiple sequences.

In addition, we can import multiple tree and alignment files at the same time. If both files have the same name, they will be combined to conduct analyses. Otherwise, PhyloSuite will permutate the tree and alignment files and produce an analysis result for each combination.

2.1.2 Select the outgroups. Note that when importing multiple trees, you can select outgroups only among the species in the first tree; so make sure that each tree has the same outgroups.

2.1.3 Select the desired analysis (more details below).

2.1.4 After the parameter configuration is complete, set the results folder name, and click the “Start” button to start the analysis (Figure 30).

Tips: after the run, the analysis results can be viewed in the results folder of “TreeSuite”. “Signal‐to‐noise (Treeness over RCV)” and “Saturation” analyses require both MSA and tree files. The “RCV (Relative composition variability)” analysis only requires the MSA file, so the input box of “Tree File (s)” as well as the combo‐box of “Outgroup(s)” will disappear when this analysis is selected. Other analyses (such as “Root‐to‐tip branch length”) only require tree files, so the input box of “Alignment File(s)” as well as the combo‐box of “Seq. type” will disappear when they are selected.

### Substitution saturation analysis

#### What is substitution saturation?

Saturated sites in a multiple sequence alignment (MSA) are those that underwent multiple substitutions, which may cause an underestimation of real genetic distances among taxa [65, 66]. To infer the saturation levels, we rely on the ratio of real and observed numbers of substitutions between two leaves. These can be expressed using the R‐squared (r^2^) between patristic and pairwise distances, which refers to the percentage of variation of the dependent variable (pairwise difference) that can be explained by the independent variable(s) (patristic distance) in a regression model [67, 68]. If multiple substitutions occur at a site, the pairwise difference will be smaller than patristic distances, thus causing low r^2^ and regression line slope values [66, 69, 70, 71].

**Box 9: Why analyze substitution saturation?**

Substitution saturation is often strongly pronounced in datasets comprising distantly‐related lineages or rapidly evolving sequences (sites). Identification and removal of loci that exhibit substitution saturation can improve the reliability of phylogenetic tree reconstruction [72, 73].

#### How to analyze substitution saturation in PhyloSuite?

2.2.1 Select the “Saturation” analysis.

**Figure 31:**
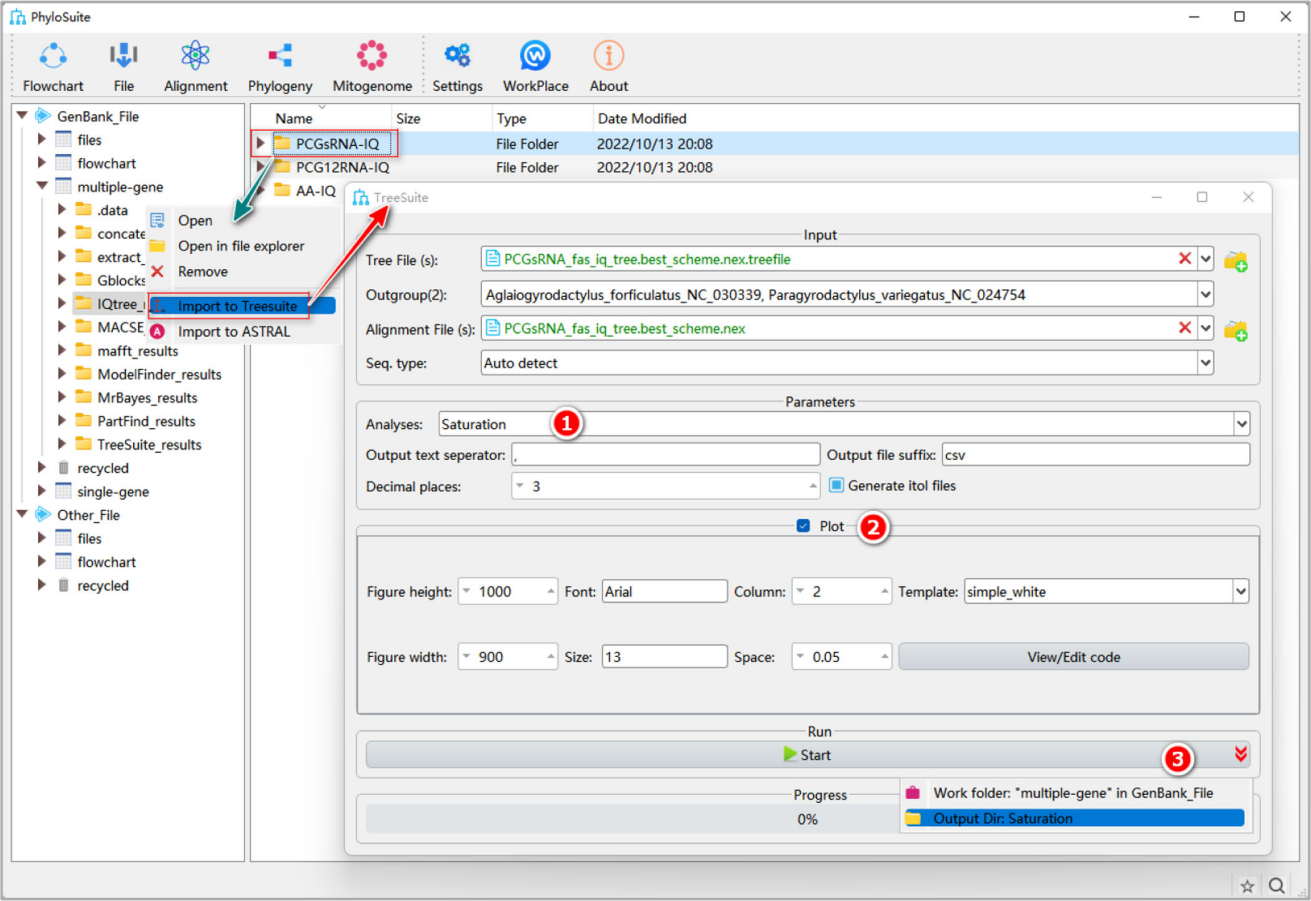
Saturation analysis in TreeSuite.

2.2.2 Check the “Plot” group box to draw a regression figure for patristic distance (x) versus pairwise difference (y), which can be used to evaluate the magnitude of saturation.

2.2.3 Set the results folder name (here we named it “Saturation”) and click the “Start” button to start the analysis (Figure 31).

**Figure 32:**
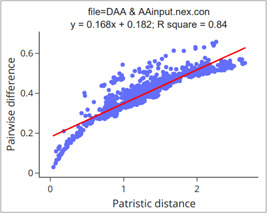
Regression analysis of saturation plots. Here we used MrBayes results based on mitogenomes of 55 flatworms for illustration.

Tips: in the result folder, the two files named “saturation.regression.pdf” and “saturation.regression.html” are the regression analysis figures (Figure 32); the file named “saturation.regression.csv” contains details of the regression analysis; the file named “saturation.csv” contains details of pairwise differences, patristic distances, and pairwise identities for pairs of species; the file named “plot_data.tsv” comprises the data used to draw the figure; the file name “plot_data.cmd.py” is the python script used to generate the above figures.

### Long‐branch score

#### What is the long‐branch score?

The long‐branch score measures the deviation (%) from the average patristic distance (PD) for each taxon, which can help users identify potential lineages that may cause long‐branch attraction artefacts (LBA) [74, 75]. It is calculated using the following formula [75]:

$${\mathrm{LB}}_{i}=\left(\frac{\bar{{\mathrm{PD}}_{i}}}{\bar{{\mathrm{PD}}_{\mathrm{all}}}}-1\right)\times 100$$

Here, *LB*_*i*_ refers to the long‐branch score of taxon *i*. $\bar{{\mathrm{PD}}_{i}}$ denotes the mean pairwise patristic distance of a taxon *i* to all other taxa in the tree. ${\mathrm{PD}}_{\mathrm{all}}$ represents the mean pairwise patristic distance of all pairwise combinations of taxa in the tree. The lower the score, the less susceptible the taxon is to long‐branch attraction [66, 75].

**Box 10: Why calculate the long‐branch score?**

The long‐branch score allows researchers to identify and exclude taxa that may produce long‐branch attraction artefacts (LBA) in phylogenetic analysis by measuring the propensity of individual taxa to produce LBA [66, 75, 76]. In comparison with the root‐to‐tip distance, which is also used as a taxon‐specific estimate of a propensity to cause LBA [77], the long‐branch score has the advantage of not being affected by the definition of the root of the tree [75].

#### How to calculate long branch score in PhyloSuite?

2.3.1 Select the “Long branch score” analysis.

**Figure 33:**
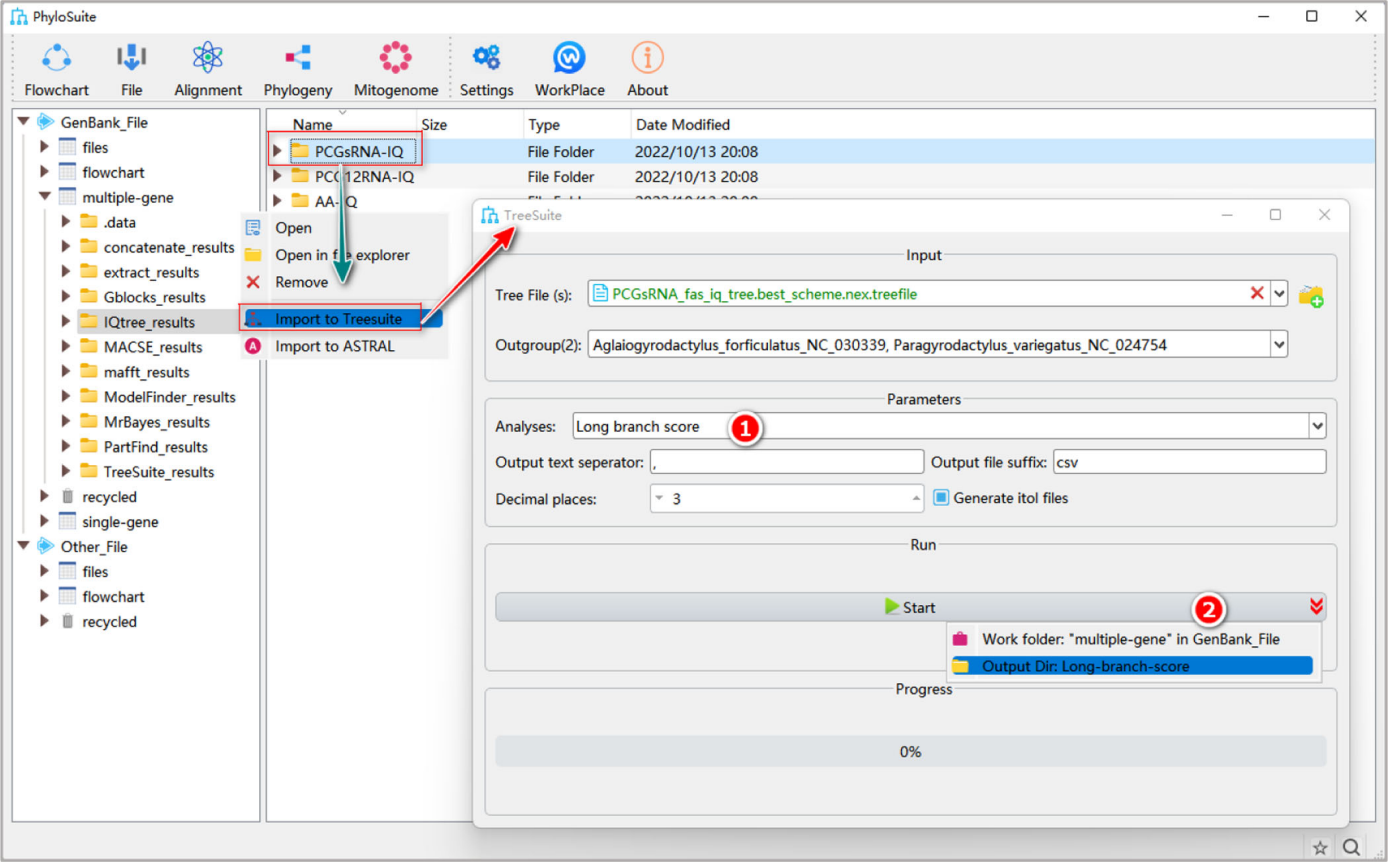
Calculation of the long‐branch score in TreeSuite.

2.3.2 Set the results folder name (here we named it “Long‐branch‐score”) and click the “Start” button (Figure 33).

Tips: in the results, the “Long branch scores.csv” file shows the long branch scores for each species; the “Long branch scores overall.csv” file shows the long branch score for the entire tree; the “*itol.txt” file can be used to draw bar plots on the tree in iTOL [78].

### Identification of “spurious species”

#### What is a spurious species?

Spurious (or “rogue”) species exhibit exceptional evolutionary patterns among the taxa included in the dataset [79]. The inclusion of spurious species may cause phylogenetic artifacts [79]. There is no default threshold for the identification of spurious species; for example, choosing the threshold of 20 defines it as a species whose terminal branch length is at least 20 times longer than the median of all branch lengths across the tree [66, 80].

**Box 11: Why identify spurious species?**

Removing spurious species can stabilize the topology of phylogenetic trees [80].

#### How to identify spurious species in PhyloSuite?

2.4.1 Select “Spurious species identification” in the combo‐box “Analyses”.

**Figure 34:**
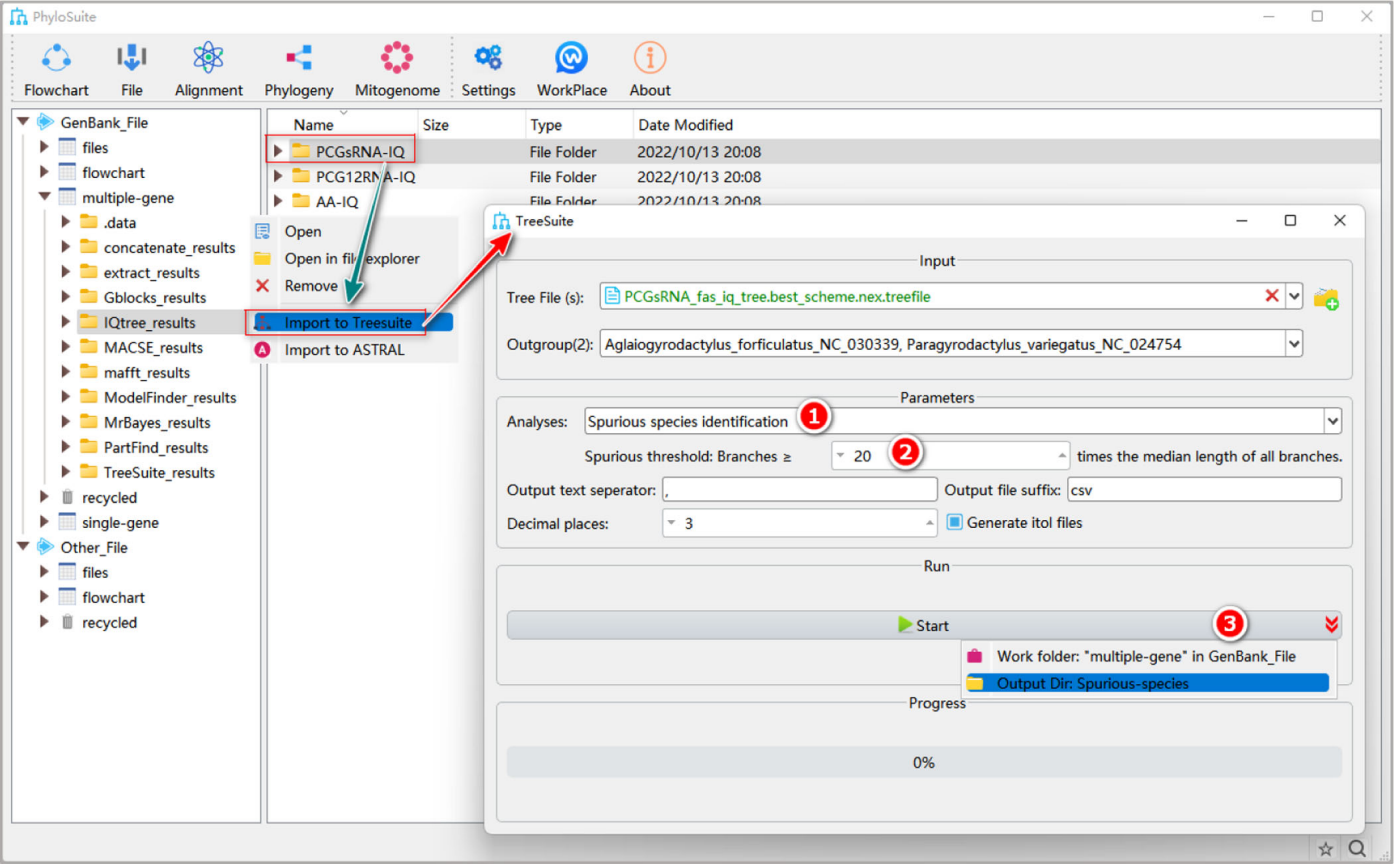
Identification of spurious species in TreeSuite.

2.4.2 Set the threshold for spurious species identification. Here we used the threshold of 20.

2.4.3 After parameter configuration is complete, set the results folder name (here we named it “Spurious‐species”) and click the “Start” button.

Tip: in the results folder, the “Spurious species.csv” file lists the identified spurious species, their branch lengths and the median length of all branches (Figure 34).

### Treeness, relative composition variability (RCV), and signal‐to‐noise ratio

#### What is treeness?

Treeness (also referred to as stemminess) is calculated by dividing the sum of internal branch lengths by the sum of all branch lengths of a tree [81, 82]. Branches of internal nodes represent synapomorphic and plesiomorphic states of characters (shared traits among organisms, presumed to derive from the common ancestor), whereas branches of terminal nodes represent accumulated autapomorphic (or unique) characters of specific organisms [81], treeness measures the proportion of evolutionary change that has taken place on internal branches of a phylogenetic tree [81]. Treeness can be used as an indicator of the signal‐to‐noise ratio in phylogeny [66, 82].

#### What is RCV?

RCV (relative composition variability) is the average compositional variability of taxa included in the MSA [82]. RCV is calculated using the following formula [66]:

$$\mathrm{RCV}=\sum_{i=1}^{c}\sum_{j=1}^{n}\frac{\left|\bar{c_{\mathrm{ij}}}-\bar{c_{i}}\right|}{n\times t}$$

where, *c* is the number of the character states (such as A, T, C, G), *n* is the number of taxa in MSA, *c*_*ij*_ is the frequency of *i* character for the *j* taxon, *c*_*i*_ is the frequency of *i* character across all *n* taxa, *t* is the length of the MSA. In addition, PhyloSuite also considers the occurrence of degenerate bases when counting the frequency; for example, Y will be regarded as 1/2 C and 1/2 T, and D will be regarded as 1/3A, 1/3G, and 1/3T.

#### What is the signal‐to‐noise ratio?

Signal‐to‐noise ratio: the comparison between the phylogenetic signal (signal used to infer the “true” tree) and the noisiness of the data (e.g., heterogeneity) [82]. The value is inferred by dividing treeness by RCV.

**Box 12: Why calculate Treeness, RCV, and signal‐to‐noise ratio?**

Treeness can be used to assess the magnitude of the phylogenetic signal. For example, if sister lineages share a very short internal branch, this indicates the existence of relatively few shared characters between the two, so they may not be resolved as sister lineages in phylogenetic reconstruction. In this way, low treeness values (which indicates short internal branch lengths) can easily cause errors in tree reconstruction based on parsimony, distance, and ML methods under simplistic models [2]. RCV can be used to evaluate the potential sequence composition biases in MSAs, also referred to as compositional homogeneity or ‘noisiness’ of data. Thus, treeness/RCV is also referred to as the signal‐to‐noise ratio. Datasets (partitions) with high treeness/RCV values are expected to exhibit a less pronounced composition bias and be more likely to reconstruct a stable tree [82].

#### How to calculate treeness, RCV and signal‐to‐noise in PhyloSuite?

2.5.1 Select “signal‐to‐noise (Treeness over RCV)” analysis, or “Treeness”, or “RCV (relative composition variability)” analyses.

**Figure 35:**
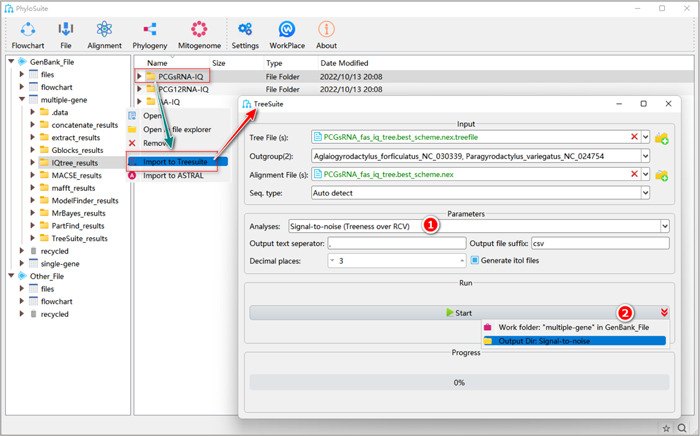
Treeness, RCV (relative composition variability) and signal‐to‐noise analyses in TreeSuite.

2.5.2 After the parameter configuration is complete, set the results folder name (here we named it “Signal‐to‐noise”) and click the “Start” button (Figure 35).

Tips: in the results folder of the “signal‐to‐noise” analysis, the “signal‐to‐ratio. csv” file displays the value of signal‐to‐noise, treeness and RCV of all species. In the results folder of “Treeness”, the “treeness. csv” file displays the treeness value of all species. In the results of “RCV (relative composition variability)”, the “Species. RCV. csv” displays the value of RCV of each species, and “RCV. csv” displays the RCV value of all species.

### Two types of branch length calculations: pairwise patristic distance and root‐to‐tip branch length

#### What are pairwise patristic distance and root‐to‐tip branch length?

Branches represent the transmission path of genetic information from the ancestor to the offspring [83]. As such, they represent the amount of genetic variation between the two.

Pairwise patristic distance: “patristic distance (also called tip‐to‐tip distance), is the sum of the branch lengths that connect the two terminal nodes in a phylogenetic tree (commonly, two species). The patristic distance matrix of all tip node pairs calculated from the phylogenetic tree summarizes the total amount of genetic or phylogenetic changes in the phylogenetic tree” [84].

Root‐to‐tip branch length: the branch length from a terminal node to the root node.

**Box 13: Why calculate pairwise patristic distance and root‐to‐tip branch length?**

Along with other measures of genetic distance, these parameters can be used to analyze the change rate in a phylogenetic tree [84, 85, 86, 87]. Specifically, the patristic distance can be used to estimate the genetic distance between any two taxa, and the root‐to‐tip branch length can be used to represent the substitution rate of a taxon or gene [86, 87]. As the time span between tip taxa and the common ancestor is the same for all species in a phylogenetic tree, all taxa in a tree should have identical root‐to‐tip branch lengths if the substitution rate does not vary across lineages [88]. Therefore, different root‐to‐tip branch lengths indicate different evolutionary rates among taxa in the dataset [1, 2, 17].

#### How to calculate the pairwise patristic distance and root‐to‐tip branch length in PhyloSuite?

2.6.1 Select the “Pairwise patristic distance (branch length)” or “root‐to‐tip branch length” analysis.

**Figure 36:**
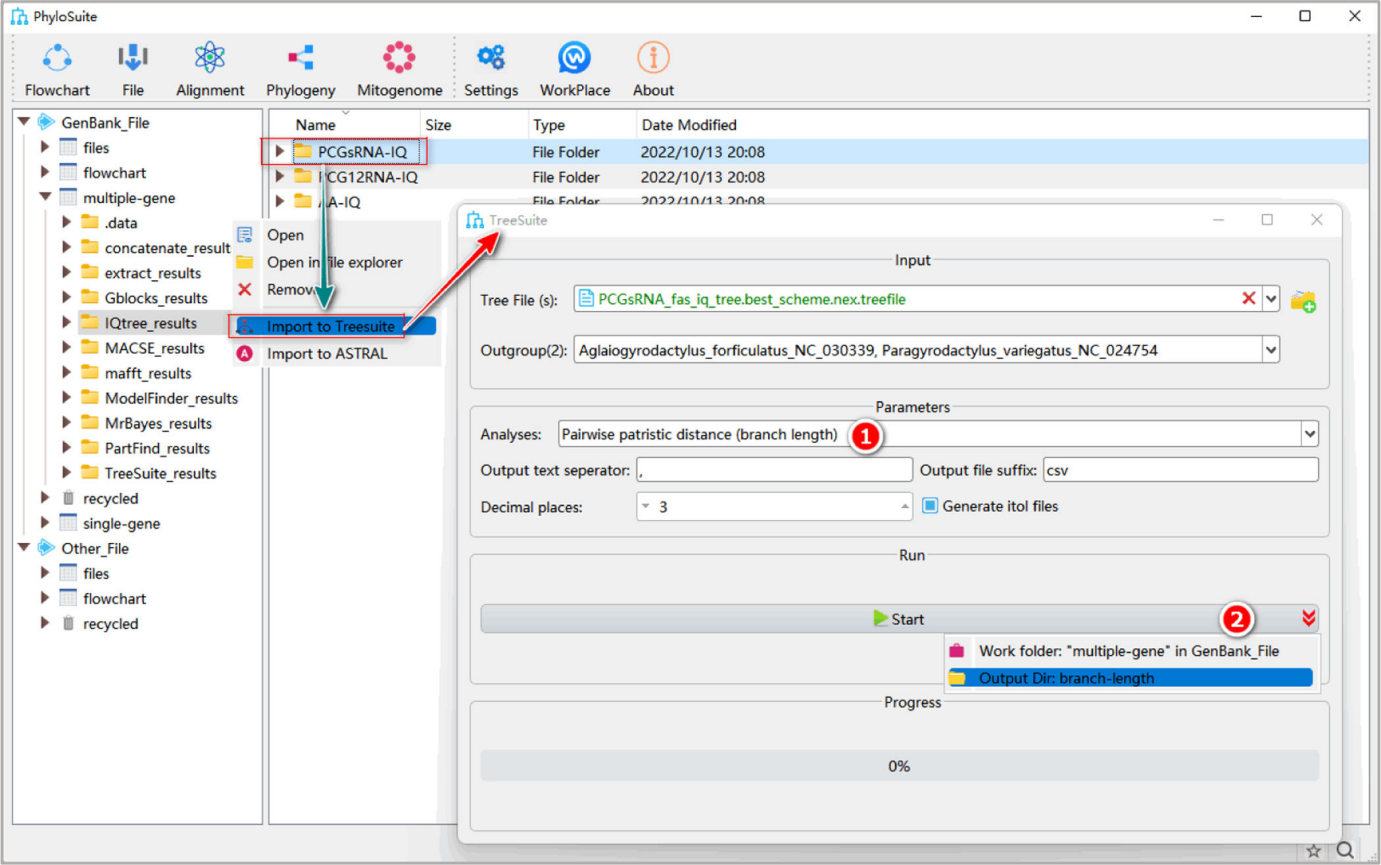
Pairwise patristic distance (branch length) and root‐to‐tip branch length calculation in TreeSuite.

2.6.2 After the parameter configuration is complete, set the results folder name (here we named it “branch‐length”) and click the “Start” button (Figure 36).

Tips: in the results folder of the pairwise patristic distance calculation, the “Patristic distance.csv” file comprises pairwise results in a table (or record) format, and the “*matrix.csv” file comprises a matrix of patristic distances. In the results folder of the root‐to‐tip branch length calculation, the “root‐to‐tip‐branch‐length.csv” file contains the value of the root‐to‐tip branch length of each species. The “*.itol.txt” file can be used to annotate root‐to‐tip branch length in a phylogenetic tree.

### Evolution rate

#### What is the evolution rate?

The evolution rate is the rate of genetic or morphological change in a lineage over a given period of time [89]. In PhyloSuite, the evolution rate can be computed by dividing the sum of the lengths of all branches (internal and terminal branches) by the total number of terminal nodes [90].

**Box 14: Why calculate the evolution rate?**

Evolutionary rates of molecular entities such as proteins, genes, etc. are of great importance in evolutionary biology [91]. For example, we may infer single‐gene trees, and then use the above method to calculate the evolutionary rate of each gene, which allows us to identify slow‐evolving and fast‐evolving genes [90].

#### How to calculate the evolution rate in PhyloSuite?

2.7.1 Select “Evolution rate.”

**Figure 37:**
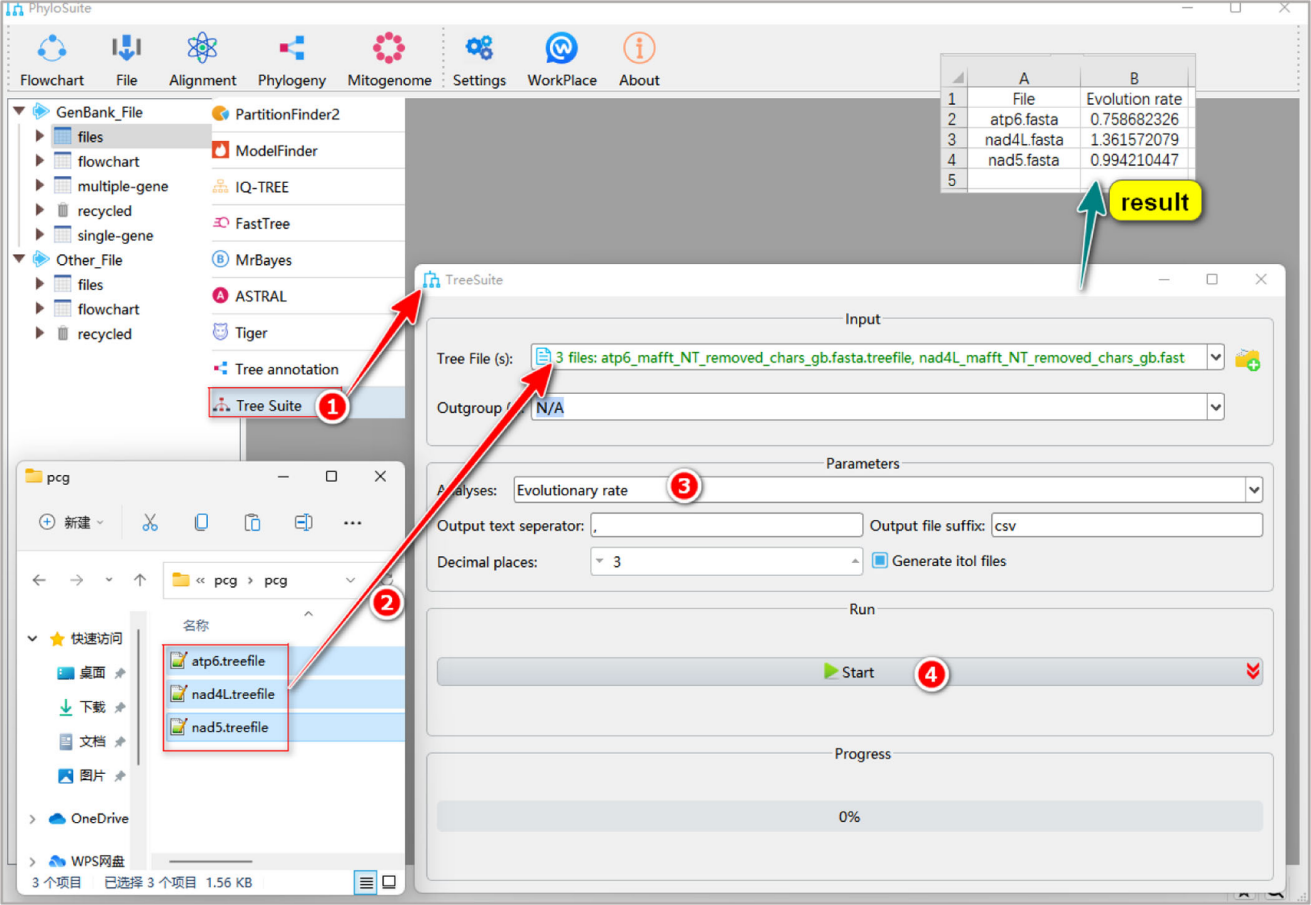
Calculation of the evolution rate of multiple tree files in TreeSuite.

2.7.2 Drag the prepared tree files (such as “*.treefile” of IQ‐TREE) into the “Tree File (s)” input box. After the parameter configuration is complete, set the results folder name (here named “Evolution‐rate”) and click the “Start” button to infer evolutionary rates (Figure 37).

Tips: in the results folder of “Evolution rate”, the “Evolution rate.csv” file contains the evolutionary rate of the entire alignment.

### The “Unroot tree” function

#### What is an unrooted tree?

A rooted tree refers to a phylogenetic tree that has a node, called the root, that predates all other nodes in the tree in evolutionary terms. Therefore, the root of a rooted tree represents the common ancestor of all extant taxa in the phylogenetic tree [17].

An unrooted tree is a phylogenetic tree that does not specify the lineage that represents the common ancestor; i.e., it is not oriented in time. Instead, it only specifies the relative branching relationships among extant taxa [17].

#### How to convert a rooted tree to an unrooted tree in PhyloSuite?

In PhyloSuite, the “Unroot tree” analysis is based on ETE3 [92] and can be used to convert a rooted tree into an unrooted tree.

**Figure 38:**
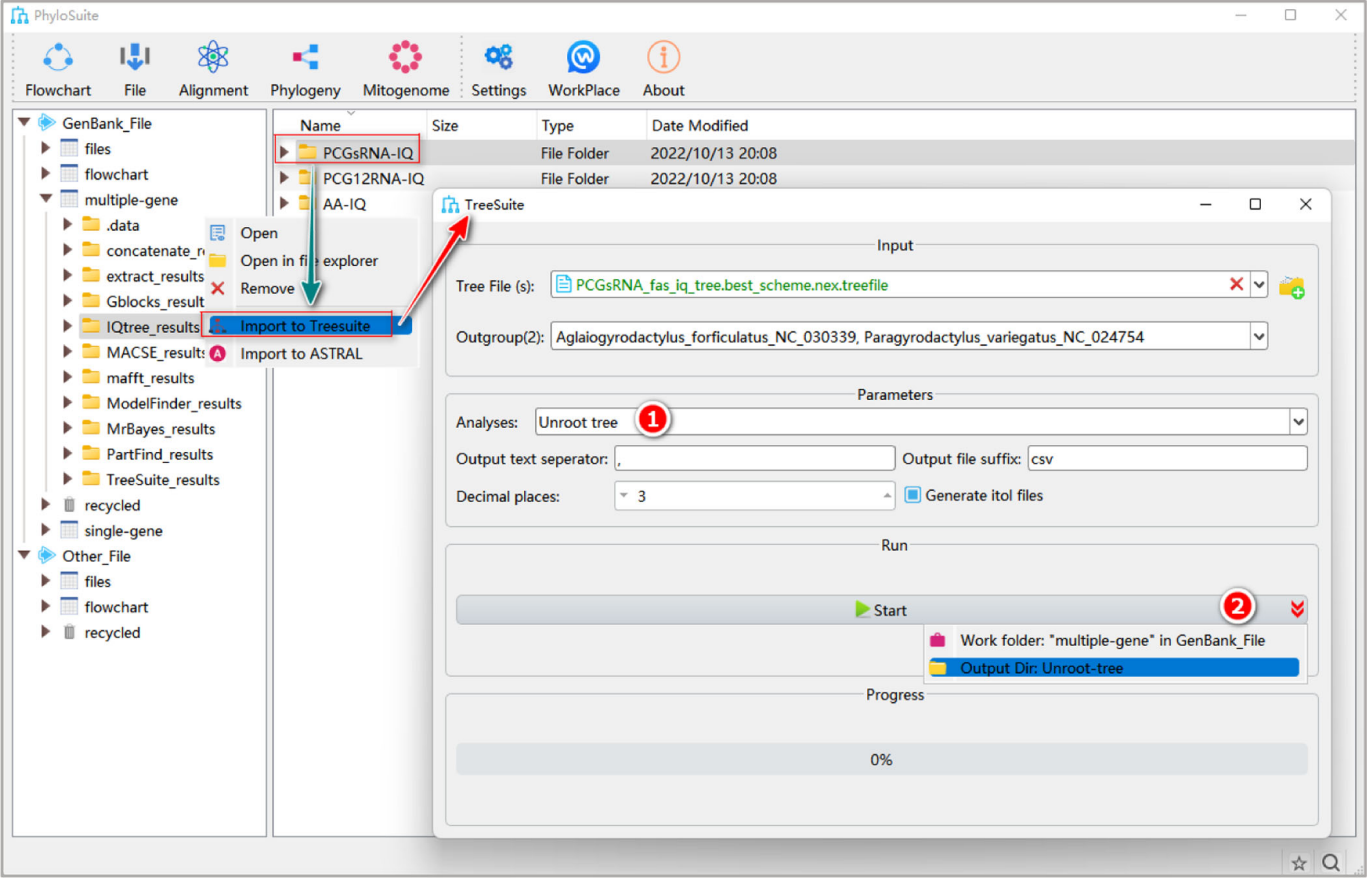
Unroot tree in TreeSuite.

2.8.1 Select “Unroot tree.”

2.8.2 After the parameter configuration is complete, set the results folder name (here we named it “Unroot‐tree”) and click the “Start” button to produce an unrooted tree (Figure 38).

Tips: the converted unrooted tree files can be found in the results folder (*.nwk).

### Resolve polytomy

#### What is polytomy?

A polytomy is a branch with three or more direct descendants of an internal node in a phylogenetic tree; a branch with only two direct descendants of an internal node is called a dichotomy [17].

**Box 15: Why resolve polytomy?**

A polytomy can occur when the evolutionary relationships among lineages are uncertain or when there is insufficient data to reconstruct the relationships with confidence [17]. As some software programs do not allow polytomy, we need to convert such clades to dichotomous clades.

#### How to resolve polytomy in PhyloSuite?

2.9.1 Select “Resolve polytomy.”

**Figure 39:**
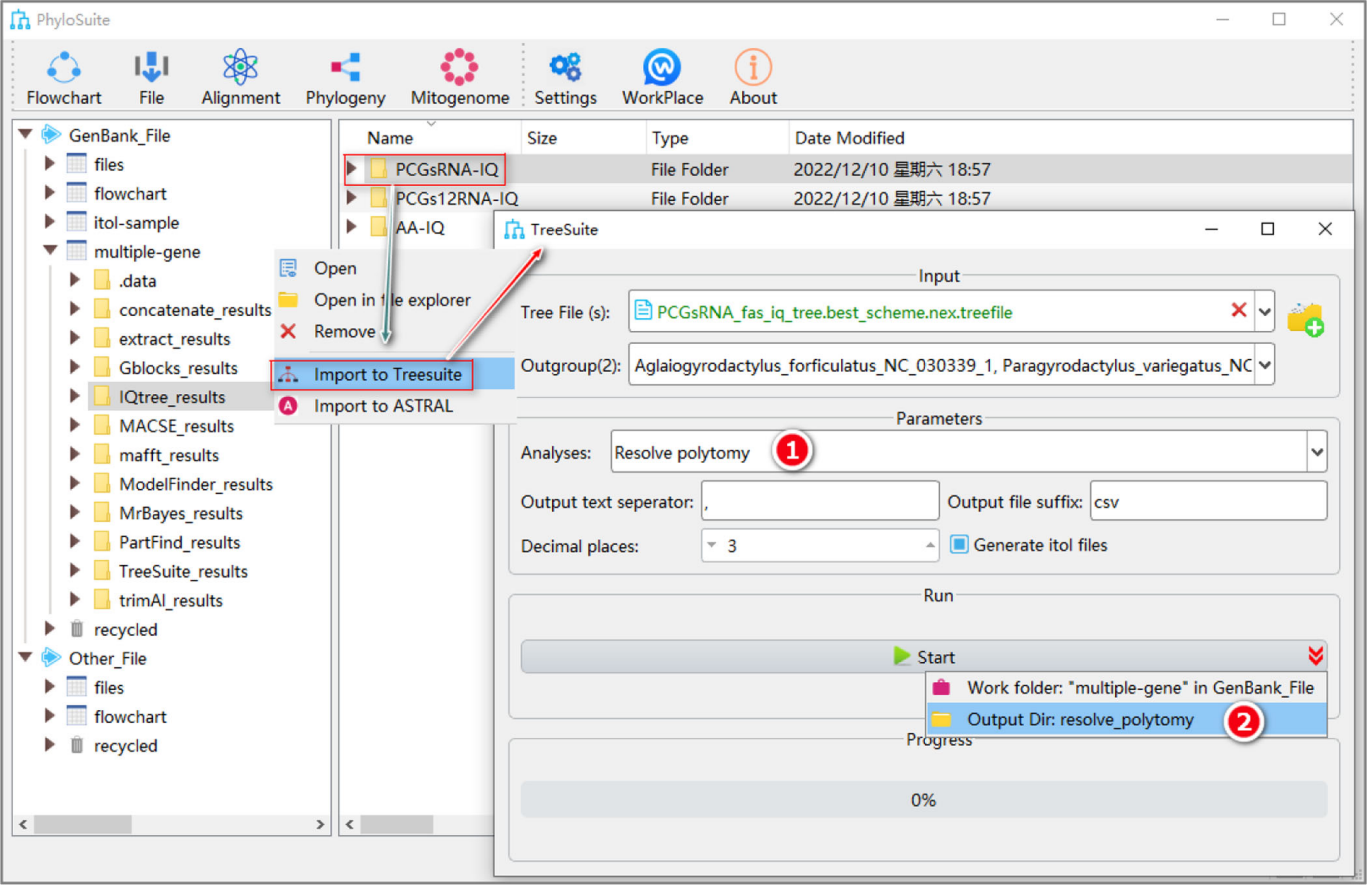
Resolve polytomy in TreeSuite.

2.9.2 After the parameter configuration is complete, set the results folder name (here we named it “resolve_polytomy”) and click the “Start” button to resolve polytomy (Figure 39).

Tips: the converted dichotomous tree files can be found in the results folder (*.nwk).

## NOTES

**Figure 40:**
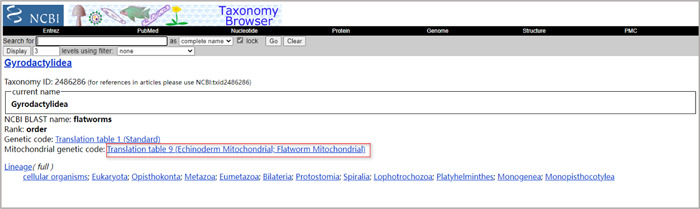
Mitochondrial genetic code for Gyrodactylidea.

Query the code table corresponding to your dataset (Sequence extraction). If you don't know which code table is appropriate for your dataset, you can find that information in the NCBI's taxonomy database (https://www.ncbi.nlm.nih.gov/taxonomy) (Figure 40).

## TROUBLESHOOTING

Troubleshooting advice for some of the most common problems is summarized in Table 1. For additional problems and explanations, please see supplementary file, section 4 (Troubleshooting).

**Table 1:** Troubleshooting: Some of the most common problems encountered by PhyloSuite users.

Step	Problem	Possible reason	Solutions
1.11.5 Stop the run and infer the tree function in MrBayes	Error message: could not rename file…	MrBayes is running and the file is occupied	Kill MrBayes process and try again
Multiple sequence alignment using MAFFT	The result file is empty	1. the path of the input file contains Chinese or other non‐English symbols; 2. the file contains only 1 sequence	1. replace the path of the input file with the path consisting of standard characters; 2. delete the input file with only one sequence
Could happen in different steps	Memory error	Running out of memory	Use the 64‐bit version of PhyloSuite, or run on a server with a larger memory
Saturation analysis and drawing the RSCU figure	Error message: plotlyjs argument is not a valid URL or file path	There are Chinese or other special symbols in the installation path of PhyloSuite	Install PhyloSuite to a path consisting of standard characters
In the supplementary file:get taxonomy (NCBI)	No apparent progress	The network is too slow to download the database	Try later, or manually download the “taxdump.tar.gz” file using the given url, then reopen this function and specify its path in the pop‐up window
Could happen in different steps	File input or results generation steps produce file errors	PhyloSuite does not have permission to operate the file	Assign folder permission for the installation path of PhyloSuite or the workplace folder

## SUPPLEMENTARY FILE

The supplementary file includes four major sections: single‐gene phylogeny, phylogenetic tree annotation using iTOL, input/output files introduction, and troubleshooting.

## AUTHOR CONTRIBUTIONS

**Dong Zhang**: Conceptualization, data curation, methodology, software, resources, supervision, writing—review and editing. **Chuan‐Yu Xiang**: Visualization, writing—original draft. **Fangluan Gao**: Conceptualization, methodology, validation. **Ivan Jakovlić**: Methodology, validation, writing—review and editing. **Hong‐Peng Lei**: Validation. **Ye Hu**: Validation. **Hong Zhang**: Validation, writing—review and editing. **Hong Zou**: Validation. **Gui‐Tang Wang**: Validation.

## CONFLICT OF INTEREST STATEMENT

The authors declare no conflicts of interest.

## Supporting information

Supporting information.

## Data Availability

PhyloSuite (v 1.2.3) is publicly accessible to all researchers and users, it is free to download and install according to the tutorial from (http://phylosuite.jushengwu.com/dongzhang0725.github.io/installation/ or https://dongzhang0725.github.io/installation/).
