# Supplementary material for: Using PhyloSuite for molecular phylogeny and tree‐based analyses
Source: Imeta. 2023 Feb 16;2(1):e87. doi: 10.1002/imt2.87 (PMC10989932; doi:10.1002/imt2.87)
Supplement: Supplementary file 1 — Supporting information. [file IMT2-2-e87-s001.docx]

**Using PhyloSuite for molecular phylogeny and tree-based analyses**

Chuan-Yu Xiang, Fangluan Gao, Ivan Jakovlić, Hong-Peng Lei, Ye Hu, Hong Zhang, Hong Zou, Gui-Tang Wang, Dong Zhang

1. **SINGLE-GENE PHYLOGENY**

To save space, the contents duplicated with the “Multi-gene phylogeny” section in the main document will not be detailed.

- 1. **Prepare GenBank IDs and download sequences**


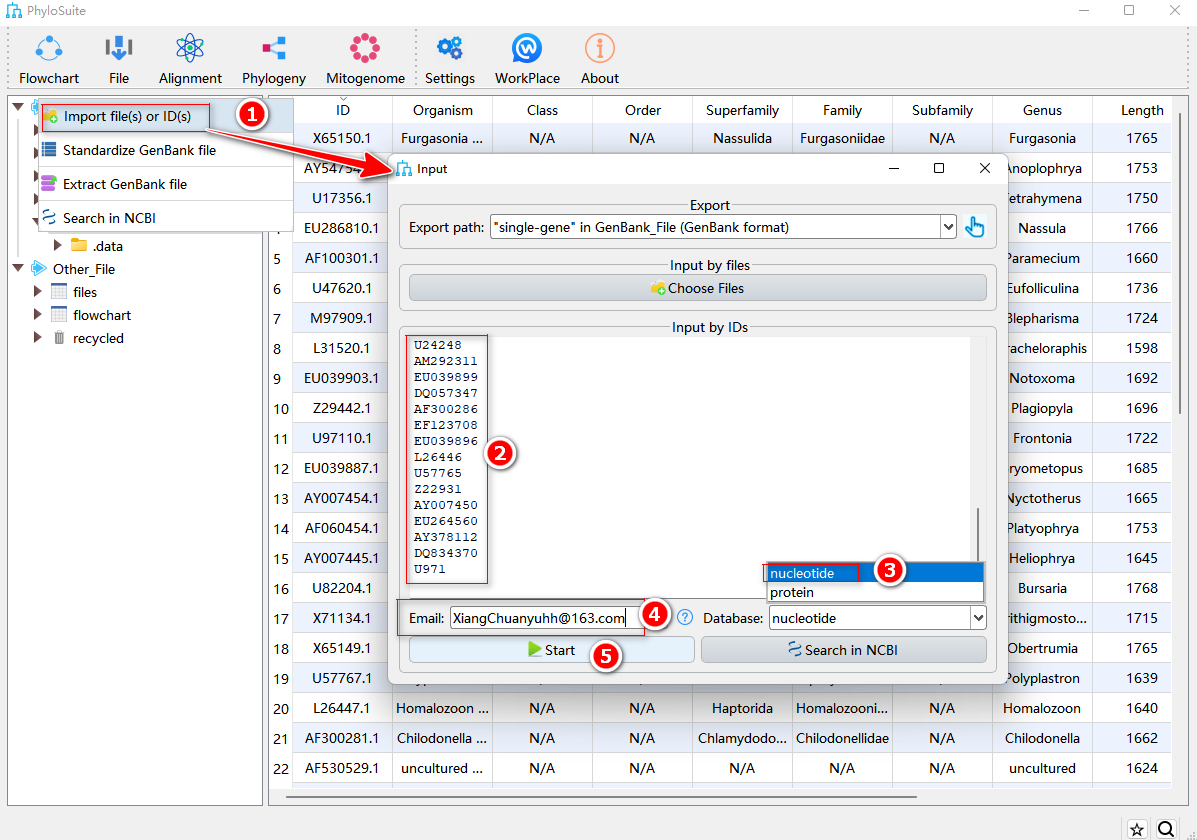


**Figure S1. Downloading sequences via IDs.**

1.1.1 Create and open a new “single-gene” work folder as shown in the multiple genes analysis. Then click the “File” menu bar and select “Import file(s) or ID(s)” to open the input window.

1.1.2 “Ctrl+A” to copy all prepared sequence IDs into the “Input by IDs” box (spaces, line breaks, tabs, etc. are all supported as separators).

1.1.3 Select a database that matches your sequences.

1.1.4 Enter your email (tell NCBI who is downloading the sequences).

1.1.5 Click “Start” to download (Figure S1).

- 1. **Extraction of single gene sequences**

The procedure for filtering sequences and finding classification information are the same as described in section “Remove redundant sequences” in the main document.


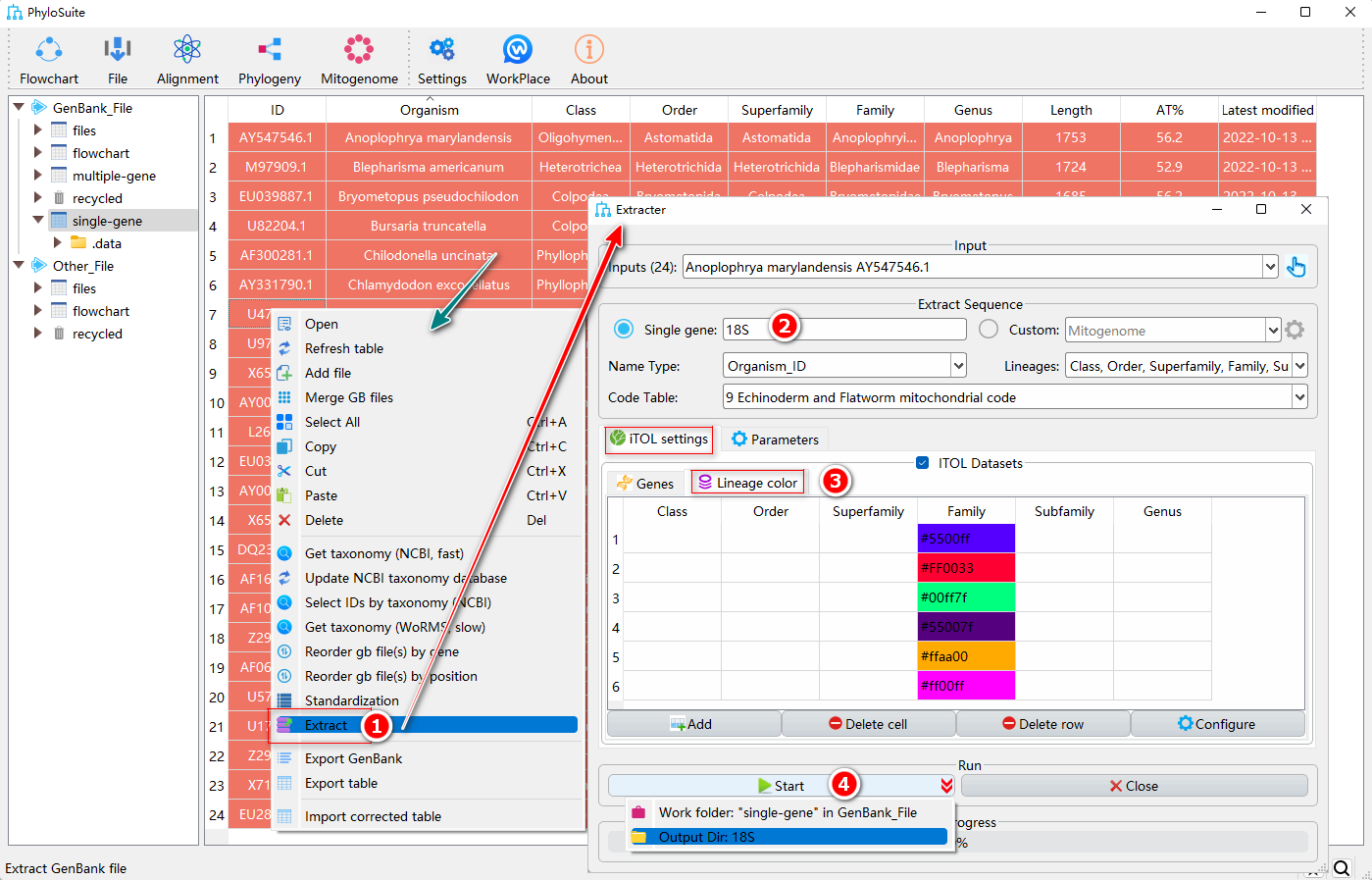


**Figure S2. Single-gene sequence extraction.**

1.2.1 Press Ctrl+A to select all imported sequences, right-click to open the context menu, and select “Extract”.

1.2.2 In the “Extracter” window, check “Single gene” and enter the gene name in the input box (such as “*18S*”).

1.2.3 Only “Lineage color” in “iTOL settings” is enabled for the “Single gene” mode.

1.2.4 Pull down the arrow of the “Start” menu to name the output file and then click the “Start” button to extract genes (Figure S2).

Other operations are the same as described in the main document.

Tip: code table will be ignored in the “Single gene” mode.

- 1. **Alignment of single-gene sequences**


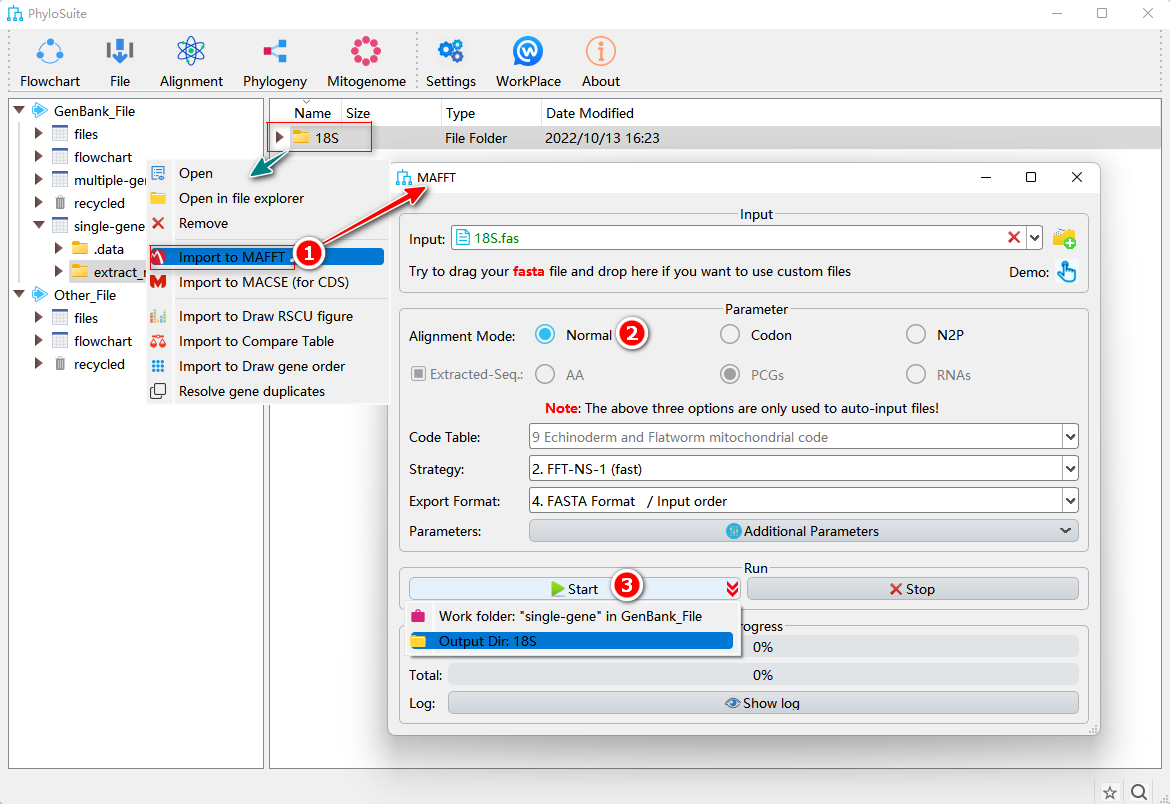


**Figure S3. Alignment of single-gene sequences.**

1.3.1 Right-click the results folder of the “Extract” function, and select “Import to MAFFT”.

1.3.2 Choose “Normal” for the “Alignment mode”. Note that the “Extracted-Seq” checkbox will be disabled in the “Single gene” mode of the “Extract” function.

1.3.3 Pull down the arrow of the “Start” menu to name the output file and then click the “Start” button to start the multiple sequences alignment (Figure S3).

Tip: since *18S* is not a protein-coding gene, MACSE optimization is not needed in this case.

- 1. **The optimal model selection**

**What is model selection?**

In molecular phylogeny, evolutionary substitution models are Markov chain models that describe substitution patterns and rates. The best-fit evolutionary model selection denotes the process of comparing a series of candidate models and selecting the one that most successfully mathematically models the observed sequence substitution process [1, 2].

**Why select the best-fit model?**

The best-fit evolutionary model can be used to calculate likelihood and evolutionary distances in phylogenetic algorithms based on likelihood and distance respectively. The selection of the appropriate substitution model is necessary to correct multiple substitutions occurring at a single site, as well as to correctly describe substitution patterns among sites and within different evolutionary lineages [1, 2].

Due to different loci and lineages often evolving at different rates, inappropriate models may reduce the accuracy of phylogenetic methods [3]. We should note here that there is some evidence that choosing the most parameter-rich model (like GTR) directly may substitute the optimal model selection step with no negative impact on the accuracy of phylogenetic reconstruction [4].

**Why use ModelFinder?**

Other model selection methods are often inferior to ModelFinder in terms of the fit between the model and data. ModelFinder is also 10 to 100 times faster than most popular competitors, jModelTest and ProtTest, which makes ModelFinder more suitable for large datasets [5].

**How to select the best-fit model in PhyloSuite?**

Alignment trimming, concatenation and partitioning are not required for single-gene sequences. Therefore, after the sequence alignment is completed, the optimal model selection is the next step.


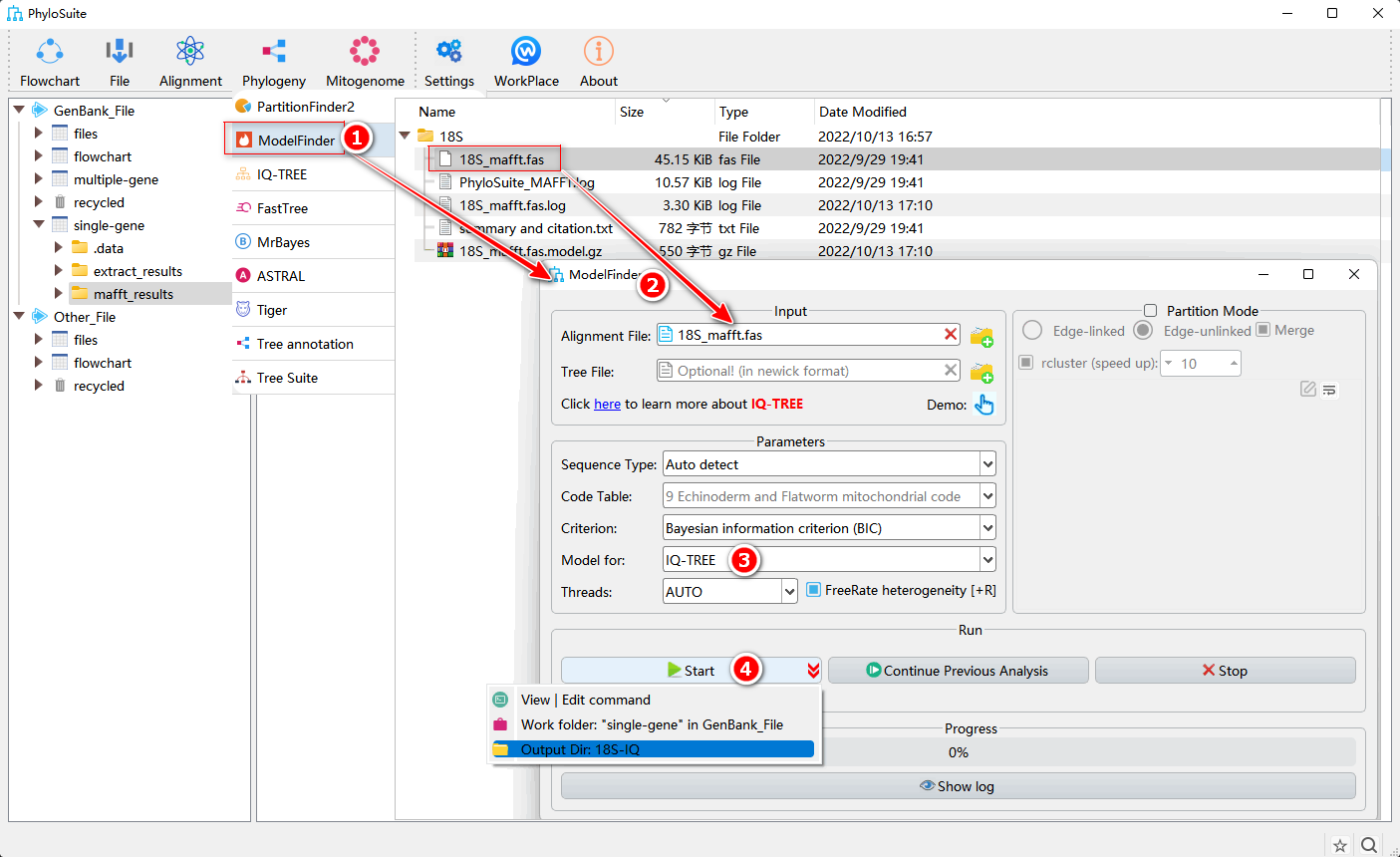


**Figure S4. Best-fit model selection using ModelFinder.**

1.4.1 Select “ModelFinder” in the “Phylogeny” menu.

1.4.2 Find the “*_mafft.fas” file in the results folder of MAFFT, then drag it into the “Alignment file” combo-box.

1.4.3 In the “Model for” combo-box, select the downstream software used for phylogenetic tree reconstruction. Here we selected the best-fit model for the “IQ-TREE” software.

1.4.4 After the parameter configuration is complete, set the results folder name (here we named it “18S-IQ”) and click the “Start” button (Figure S4).

Tip: the best-fit model selection results based on three criteria (AIC, BIC and AICc) can be found in the “*.iqtree” file.

**How to select the best-fit model for MrBayes?**

The operations are the same as in 1.4.1 - 1.4.4, but select “MrBayes” in the “Model for” combo-box. The best-fit models based on three criteria (AIC, BIC and AICc) can be found in the “*mrbayes.iqtree” file.

- 1. **Maximum likelihood tree reconstruction** **using IQ-TREE**


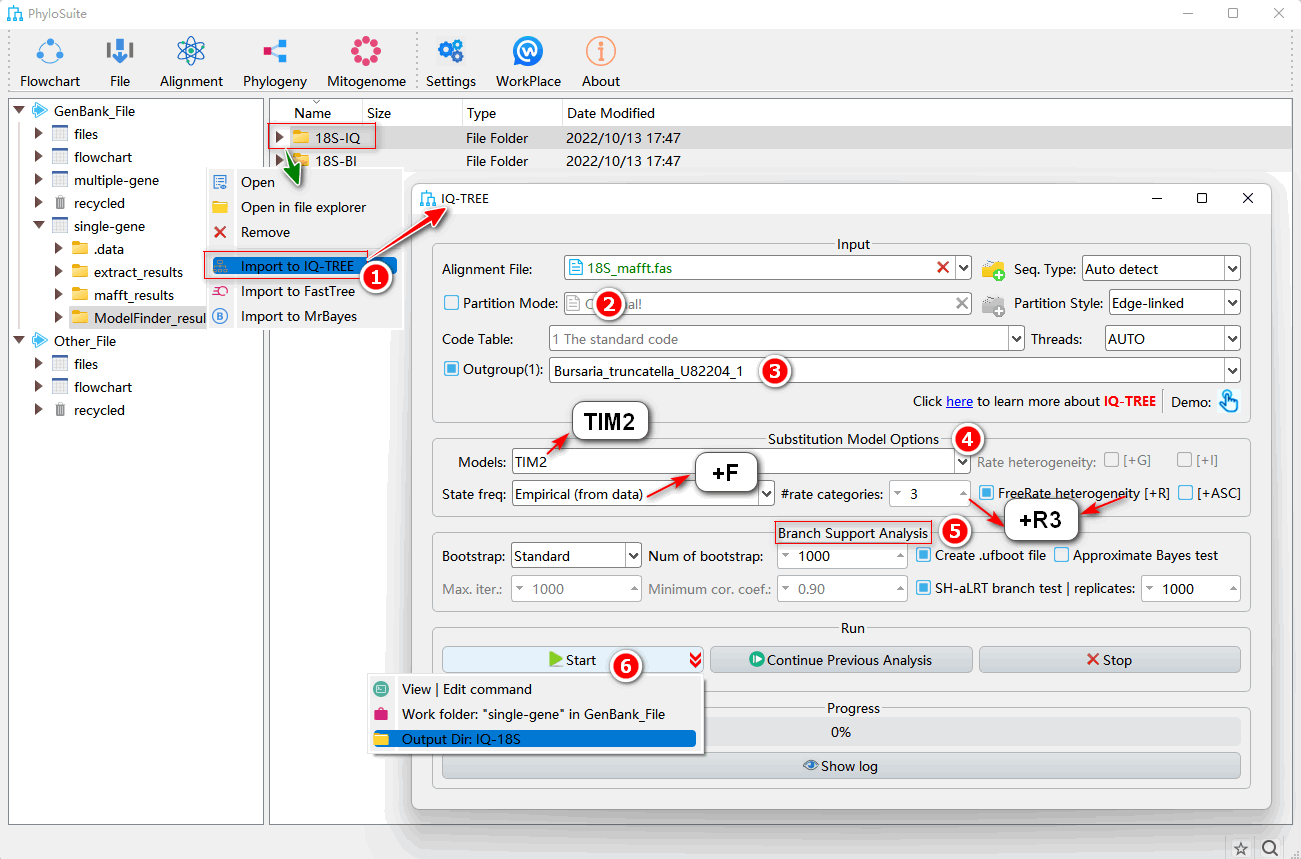


**Figure S5. Maximum likelihood tree reconstruction using IQ-TREE.**

1.5.1 Right-click the results folder of “ModelFinder” and select “Import to IQ-TREE”. The alignment file and the best-fit model (TIM2+F+R3) will be automatically imported to the “Alignment File” and “Substitution Model Options”, respectively.

1.5.2 Make sure to uncheck the “Partition Mode”. This allows the program to use the settings (i.e. the imported best-fit model) in the “Substitution Model Options” group box.

1.5.3 Select outgroups.

1.5.4 The best-fit model parameters have been automatically set during the input step.

1.5.5 According to the IQ-TREE manual, for a single gene (short alignment) you should select “Standard” in the “Bootstrap” combo-box, and set the “Num of bootstrap” to 1000.

1.5.6 After the parameter configuration is complete, set the results folder name (here we named it “IQ-18S”) and click the “Start” button to start building a phylogenetic tree (Figure S5).

Tip: the file named “*.treefile” is the phylogenetic tree.

- 1. **BI tree reconstruction**


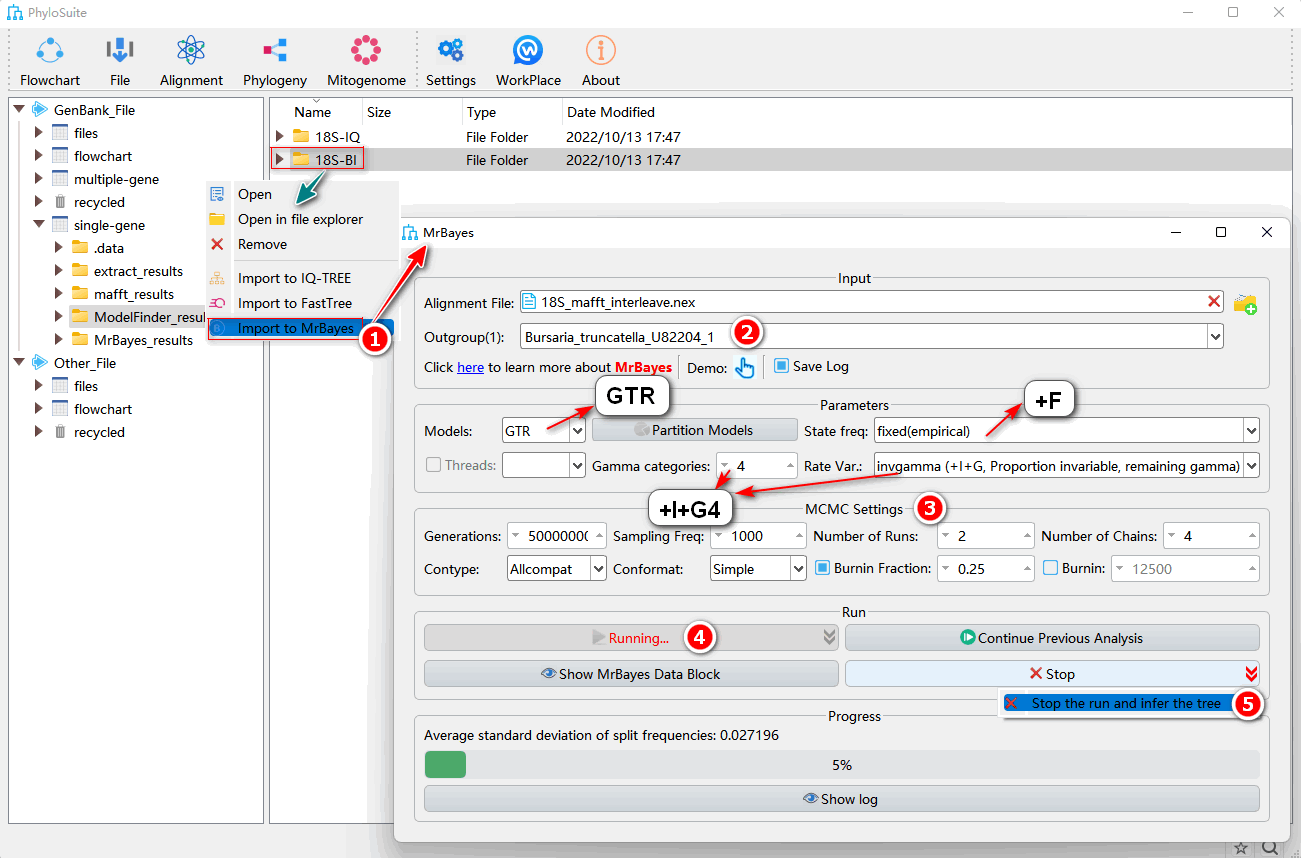


**Figure S6. Phylogenetic tree reconstruction based on Bayesian inference algorithm implemented in MrBayes.**

1.6.1 Right-click the results folder of “ModelFinder” and select “Import to MrBayes”. The alignment file and best-fit models (GTR+F+I+G4) will be automatically imported.

1.6.2 Select outgroups.

1.6.3 For “MCMC Settings” refer to the “Multi-gene phylogeny” section in the main document (1.11.3).

1.6.4 After the parameter configuration is complete, set the results folder name (here we named it “BI-18S”) and click the “Start” button to start building a phylogenetic tree.

1.6.5 When the value of “Average standard deviation of split frequencies” is below 0.01, click the down-arrow of the “Stop” button and select “stop the run and infer the tree” to stop the program and obtain relevant result files. Check the summary statistics, such as PSRF and ESS, as mentioned in the main document (“How to evaluate the convergence of BI run?”) to judge whether the BI run is convergent (Figure S6). You can also continue the BI run if it hasn’t converged (for operation, see the section “What to do if the BI run did not converge?” in the main document ).

Tips: the file named “*.con.tre” is the phylogenetic tree file.

1. **PHYLOGENETIC TREE ANNOTATION USING iTOL**

As opposed to other inbuilt functions (or plug-in programs) described herein, iTOL has not been integrated into PhyloSuite. Instead, PhyloSuite merely produces files that can be used to annotate your trees in iTOL. Therefore, before performing subsequent operations for tree annotation, it is necessary to register and log-in to the iTOL website: (https://itol.embl.de). Note: users may notice that this version of PhyloSuite comes with a beta version of an inbuilt tree annotation function; however, this function remains unfinished (Nov. 2022), so in this tutorial, we will only explain how to annotate trees using iTOL.

**What is tree annotation?**

Tree annotation refers to the process of visualization of phylogenetic trees, which may include the use of additional datasets, such as taxonomic information, genome structure, genetic traits, etc. [6].

**Why annotate phylogenetic trees?**

Phylogenetic tree annotation allows evolutionists to quickly and easily interpret phylogenetic data (such as morphological and chemical characters, life history traits, etc.) under a phylogenetic framework, which is essential for the accurate identification of evolutionary events.

**How to annotate a tree in iTOL?**

Here we will use MrBayes results based on mitogenomes of 55 flatworms (including 53 species of trematodes as the ingroup and two species of Monogenea as the outgroup) to demonstrate tree annotation in iTOL.

2.1 First open the iTOL’s interface at https://itol.embl.de , log in and select “My Trees”.

2.2 Find the tree file (*.con.tre) in the MrBayes result folder on your computer, and drag-and-drop the file into the “Tree upload” interface (Figure S7).


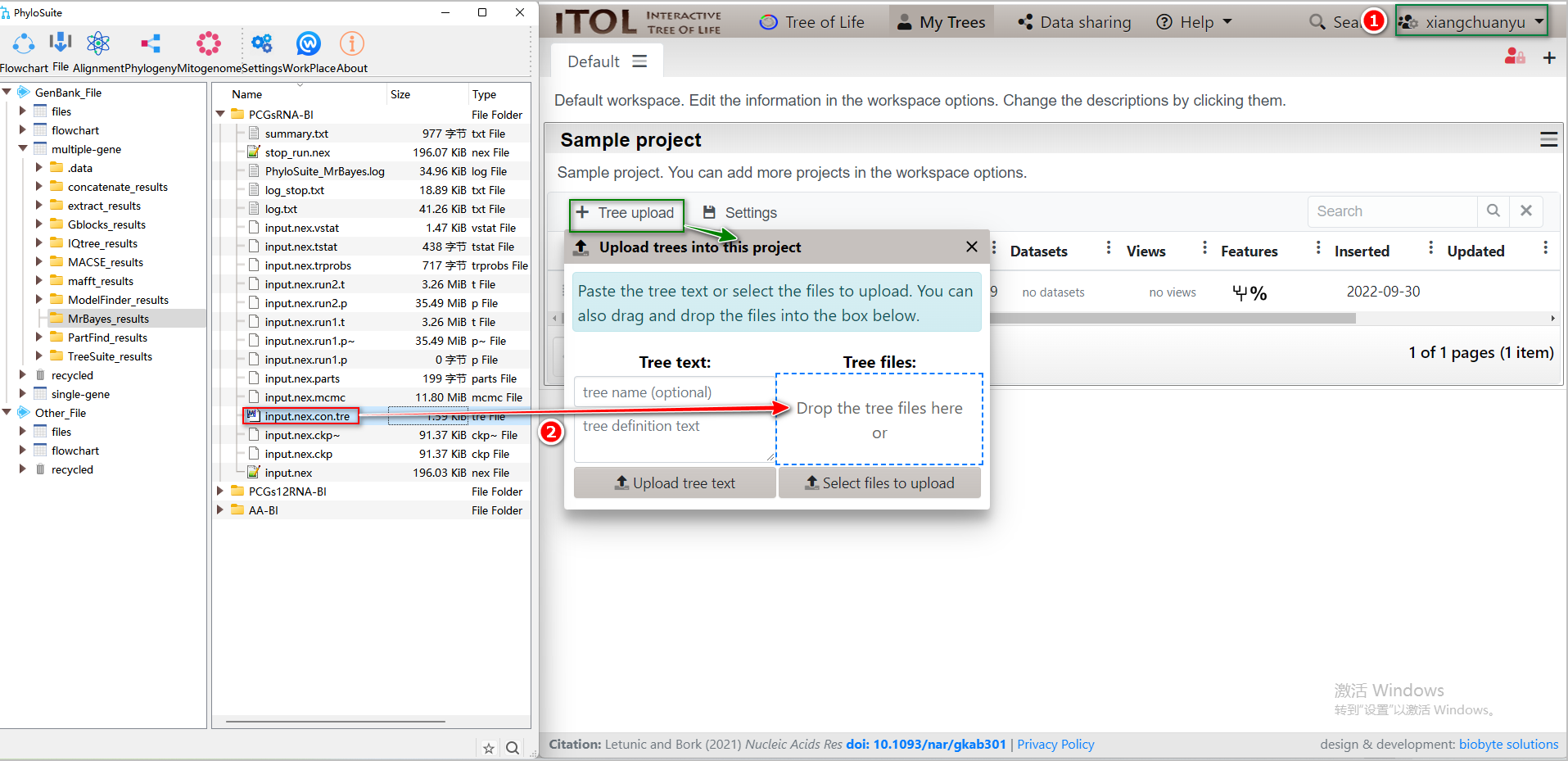


**Figure S7. Importing a tree file into iTOL.**


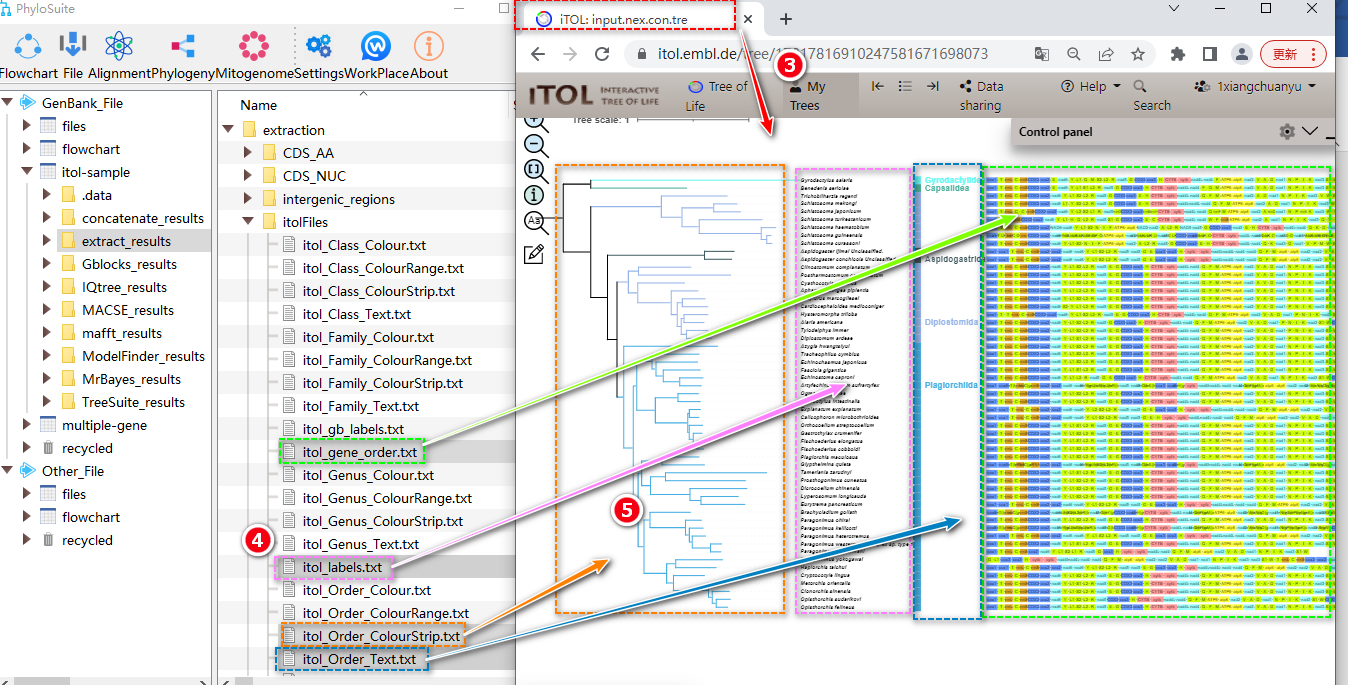


**Figure S8. Annotating a tree using iTOL dataset files produced by PhyloSuite.**


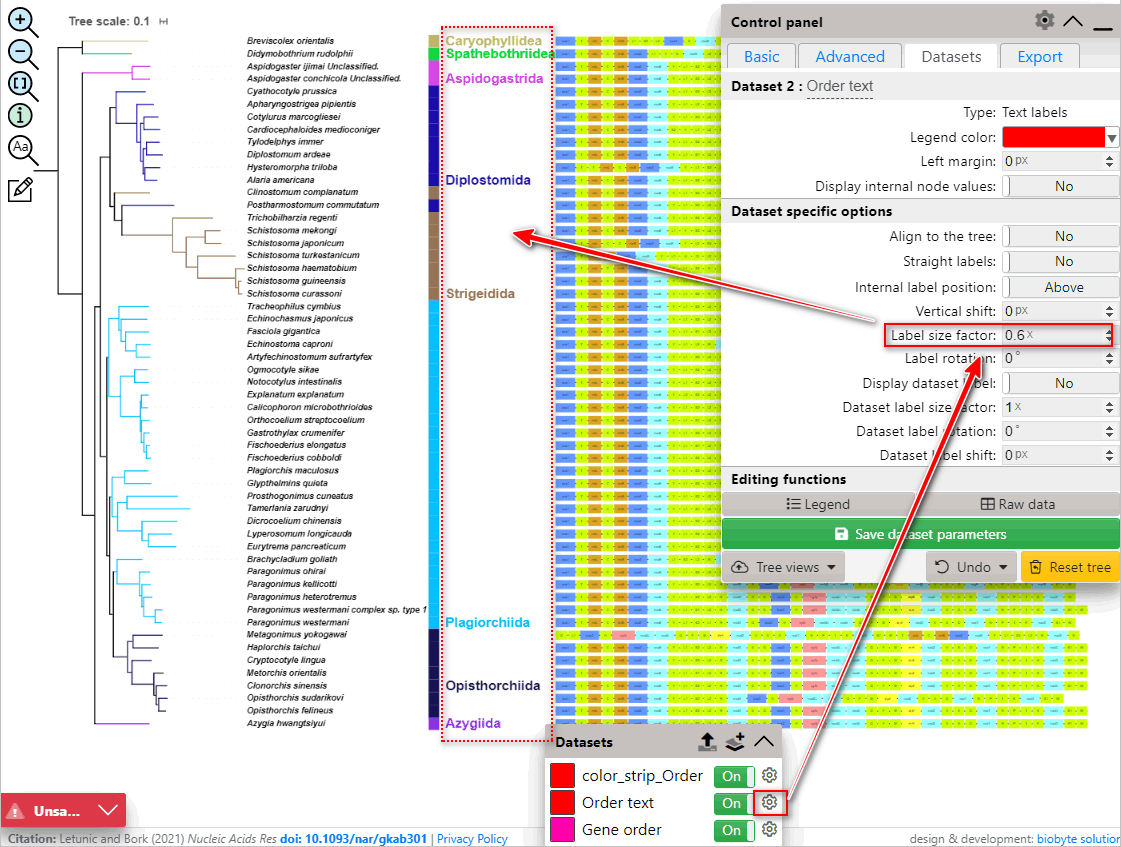


**Figure S9. Adjusting the annotation in iTOL.**


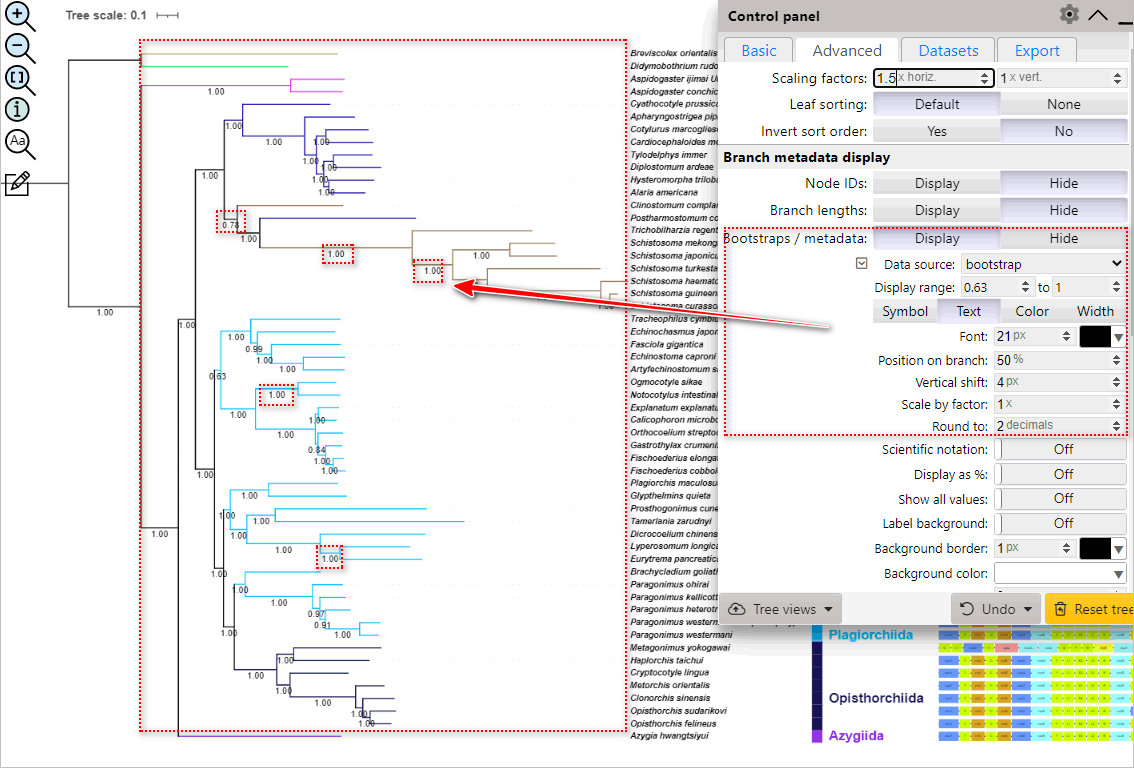


**Figure S10. Adjusting bootstrap info in iTOL interface.**


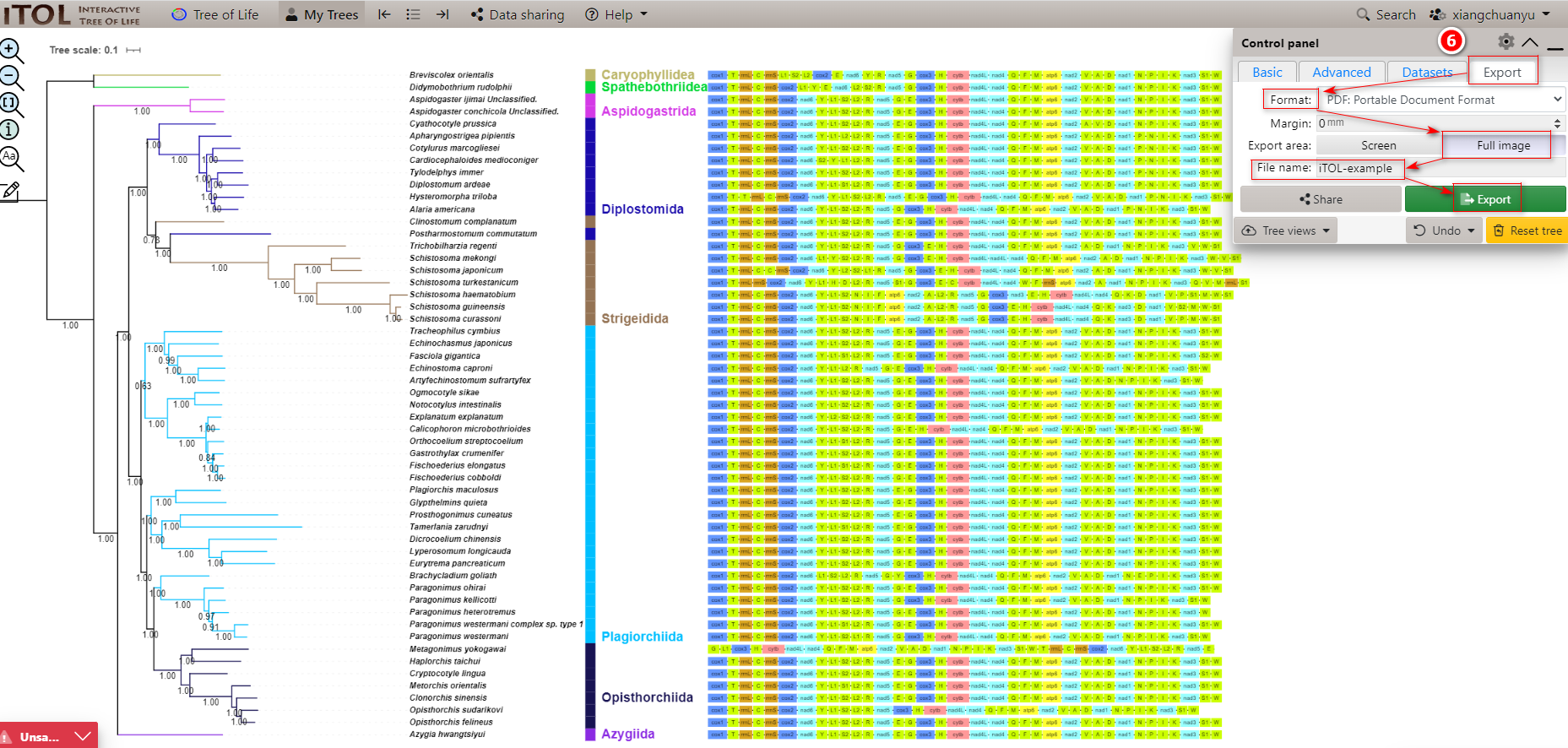


**Figure S11. Exporting the annotated tree.**

2.3 Double click the tree name to enter the tree-editing interface. Go to the results folder of the “Extract” function (under the “extract_results” folder, see the “Sequence extraction” section in the main document). All files within the “itolFiles” folder can be used for iTOL annotation. Just drag any file into the iTOL interface to make an annotation. For example, the “itol_labels.txt” file can be used to replace the default species name with the Latin name.

2.4 Check file names to see which taxonomic category information they carry. For example, “itol_Order_ColourStrip.txt” and “itol_Order_Text.txt” files can be used to annotate (colorize) different orders.

2.4.1 Drag these two files (itol_Order_ColourStrip.txt and itol_Order_Text.txt) together into the iTOL interface (Figure S8).

2.4.2 Each file is treated as a different dataset by the iTOL. You can set the parameters for each dataset by clicking the “settings” button.

2.4.3 Set the “Text size factor” of the Class text dataset to change the font size for the name of the class (Figure S9).

2.4.4 Set the “Strip width” of the color_strip dataset to change the width of the vertical block.

2.4.5 Bootstrap values and related options can be set in the “Bootstraps/metadata” option of the “Advanced” tab (Figure S10).

2.5 After you’ve finished annotating the phylogenetic tree, click “Export” and adjust parameters such as the output file name and format (Figure S11).

Tips: when annotating the phylogenetic tree produced by IQ-TREE, just drag the tree file (*.treefile) into iTOL, and the rest of the operations are the same as for the BI tree (*.con.tre). Note that the files used for iTOL annotation are all generated in the “Sequence extraction” step. Also note that iTOL charges a fee if users wish to save their annotated trees permanently in its database. If you decide to use their free service, trees will remain in the iTOL database after you exit the interface, but the next time you try to access them, they will revert back to their original appearance (i.e. unannotated tree). Accordingly, we recommend that you export the trees immediately after you annotate them.

1. **INPUT/OUTPUT FILES INTRODUCTION**

PhyloSuite and its plugin programs use standard file formats: GenBank, Fasta, Phylip, Nexus, and some others (Table S1). Examples of input files are available at http://phylosuite.jushengwu.com/dongzhang0725.github.io/example/.

Table S1. Input/output files for major plugin software programs in PhyloSuite.

| Steps | input file format | main output file(s) |
| --- | --- | --- |
| import files | Genbank; Fasta | Sequence record in PhyloSuite |
| extraction | Sequence record in PhyloSuite | gene sequence files: *.fas;  annotation files for iTOL: itol*.txt;  statistics files: *.csv |
| MAFFT | Fasta | aligment files: *_mafft.fas |
| MACSE | Fasta | *_NT.fas: nucleotide alignment files generated by MACSE;  *NT_removed_chars.fas: *_NT.fas files with special characters removed (can be used directly by downstream software programs);  *_AA.fas: amino acid alignment files generated by MACSE;  *AA_removed_chars.fas: *_AA.fas files that removed special characters (can be used directly by downstream software programs) |
| Gblocks | Fasta | *_gb.fasta: trimmed alignment files |
| trimAl | Fasta | *_trimAl.fas: trimmed alignment files |
| HmmCleaner | Fasta | *_hmm.fasta: trimmed alignment files; *_ hmm.score: the alignment between the original sequence, the score observed by and the output sequence |
| concatenation | Fasta, Phylip, Nexus | various formats of concatenated sequences: *.fas; *.nex; *.axt; *.phy; *.PML |
| ModelFinder | Alignment in Fasta, Phylip, Nexus formats and/or preset partition file in Nexus format | **non-partition**: *.iqtree: includes the optimal evolutionary model  **partition:** *.best_scheme (used for RAxML)/*.best_scheme.nex (used for IQ-TREE): contains the optimal partitioning strategy and best-fit models for each partition |
| PartitionFinder | Alignment in Phylip format and preset partition file in text format | best_scheme.txt (in the analysis folder) and best_scheme_and_models.csv: contains the optimal partitioning strategy and best-fit models for partitions |
| IQ-TREE | Alignment in Fasta, Phylip, Nexus formats and/or best partition scheme file in nexus format | *.treefile: tree file |
| MrBayes | Alignment in Nexus format and/or best partition scheme within it | *.con.tre: tree file |
| TreeSuite | **tree file:** Newick, Nexus, Nexml and Phyloxml  **alignment file:** Fasta, Phylip, Nexus | In general: *.csv: statistics files; *itol.txt: annotation files for iTOL; *.pdf: figure. |

Note that if you run the above functions using the results of other functions in PhyloSuite as input, all input requirements listed above will be automatically satisfied by PhyloSuite.

The details of output files for certain steps are listed below.

- 1. **The output files of extraction**


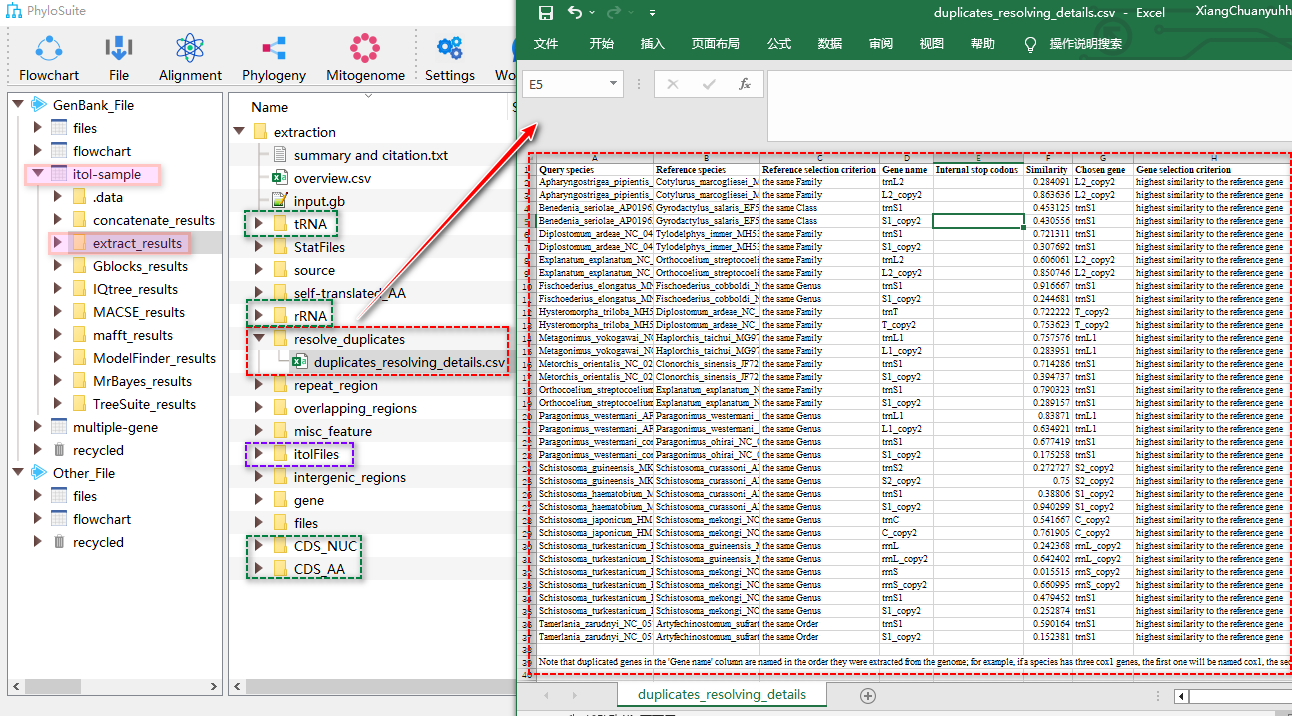


**Figure S12. Output files of the extraction step.** **Here we selected the “extract_results” results folder of the “itol-sample” work folder for illustration.**

Extracted tRNA, rRNA, PCGs, and AA sequences are available in Fasta format (*.fas) in folders “tRNA”, “rRNA”, “CDS_NUC”, and “CDS_AA” respectively. The files for annotation in iTOL are stored in the “itolFiles” folder. The information about duplicated genes, and how duplicates were resolved, is stored in the file “duplicates_resolving_details.csv” of the “resolve_duplicates” folder (Figure S12).

- 1. **The input/output files of ModelFinder**


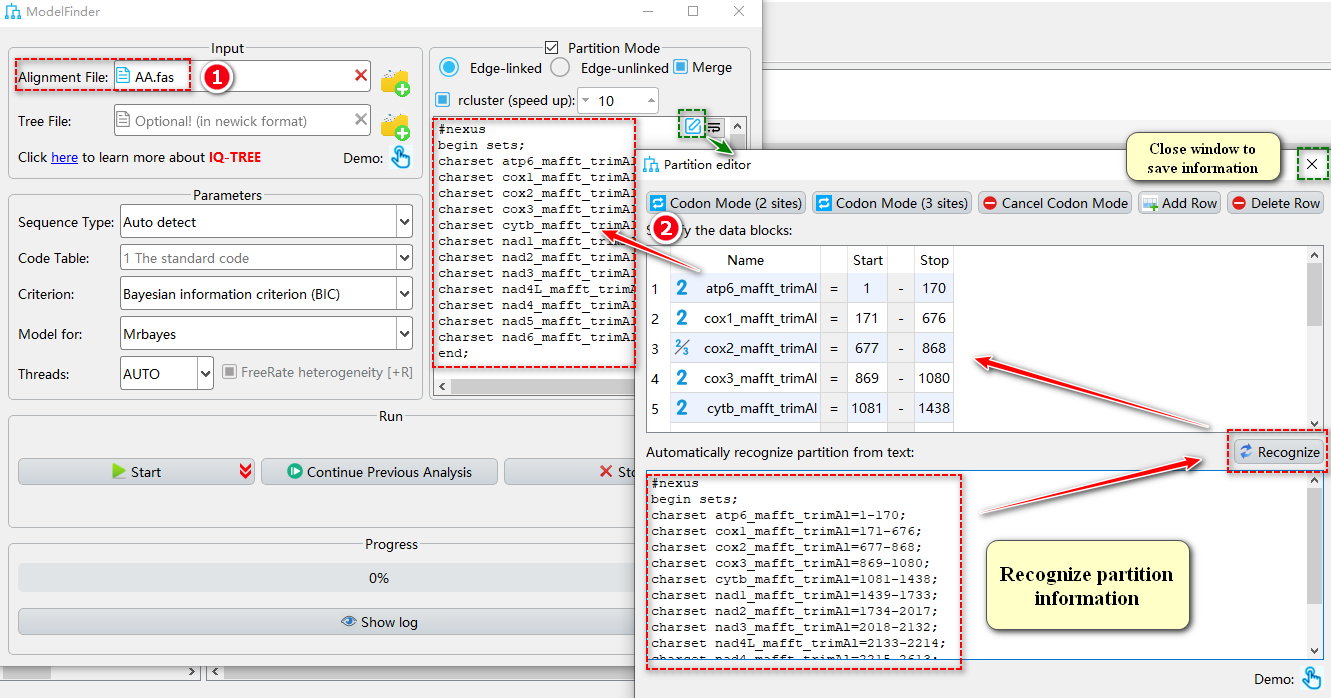


**Figure S13. The input files for ModelFinder (partition mode).**


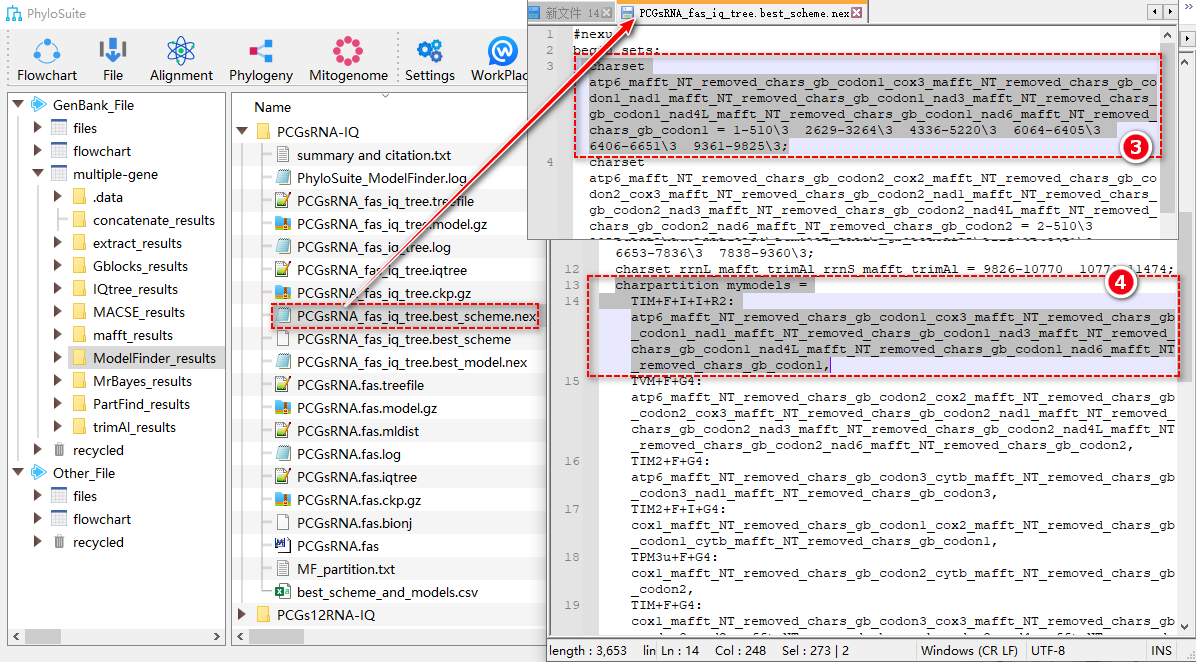


**Figure S14. The output files of ModelFinder (partition mode).**


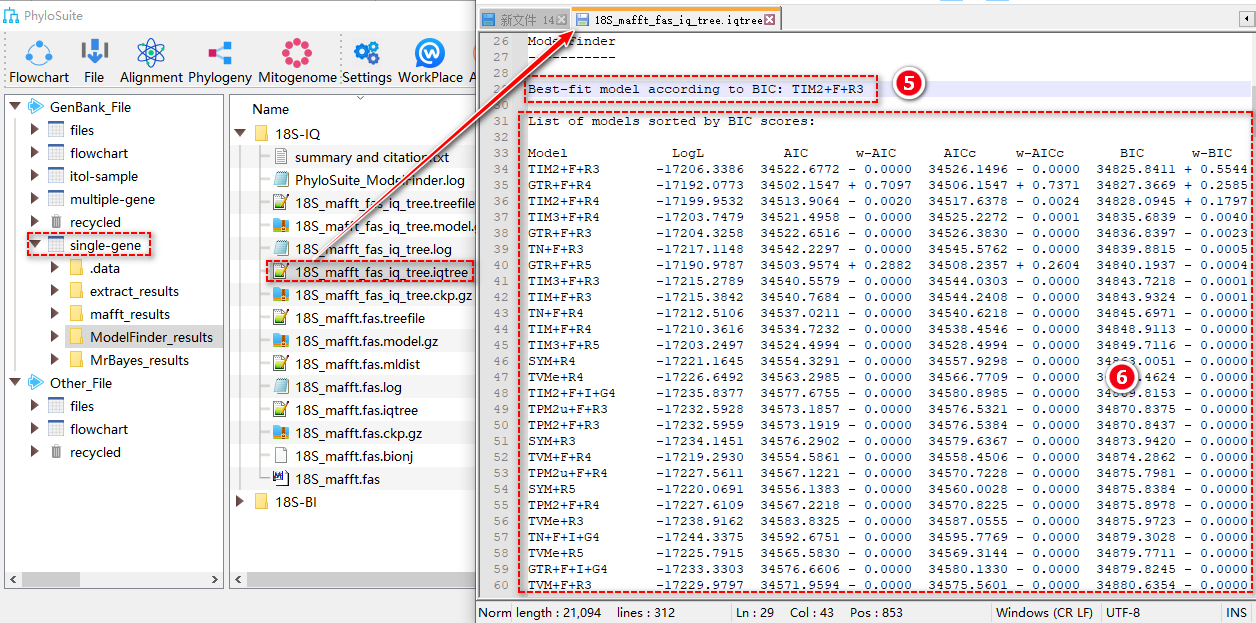


**Figure S15. The output files of ModelFinder (non-partition mode).**

3.2.1-3.2.2 Alignment file (*.fas) and preset partition file in text format (Figure S13).

3.2.3 A “charset” describing the optimal partitioning strategy, i.e. describing which preset partitions belong to the same subset, as well as their corresponding index (Figure S14).

3.2.4 “charpartition” describing best-fit models for each subset (Figure S14).

3.2.5 The optimal model selected based on the BIC criterion (Figure S15).

3.2.6 Models sorted by BIC scores, where lower scores indicate a better fit of the model (Figure S15).

- 1. **The input/output files of PartitionFinder**


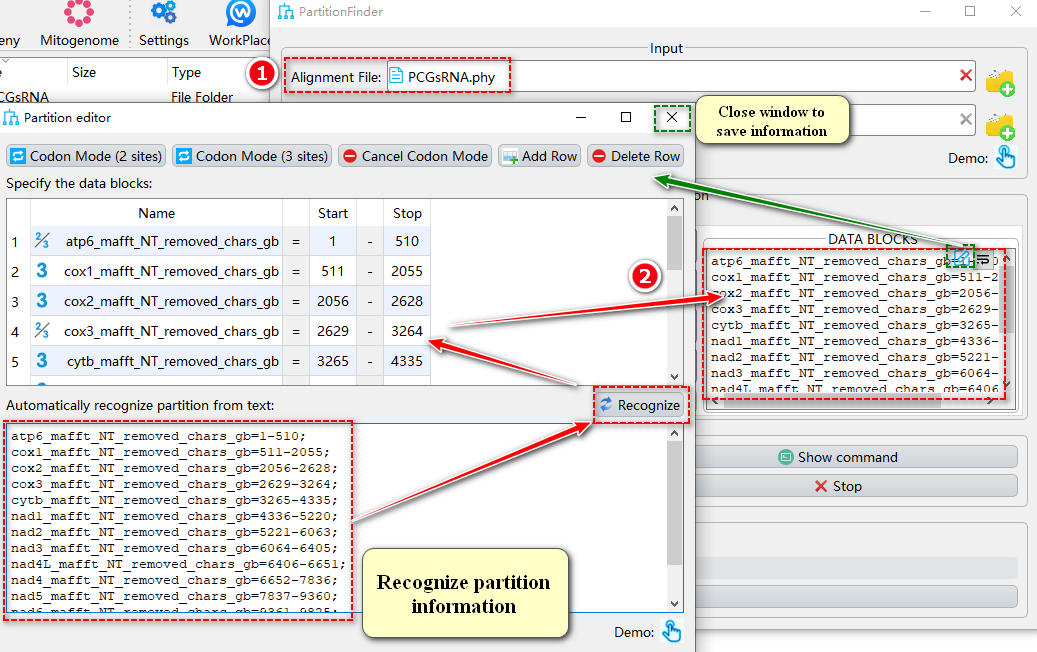


**Figure S16. The input files for PartitionFinder.**


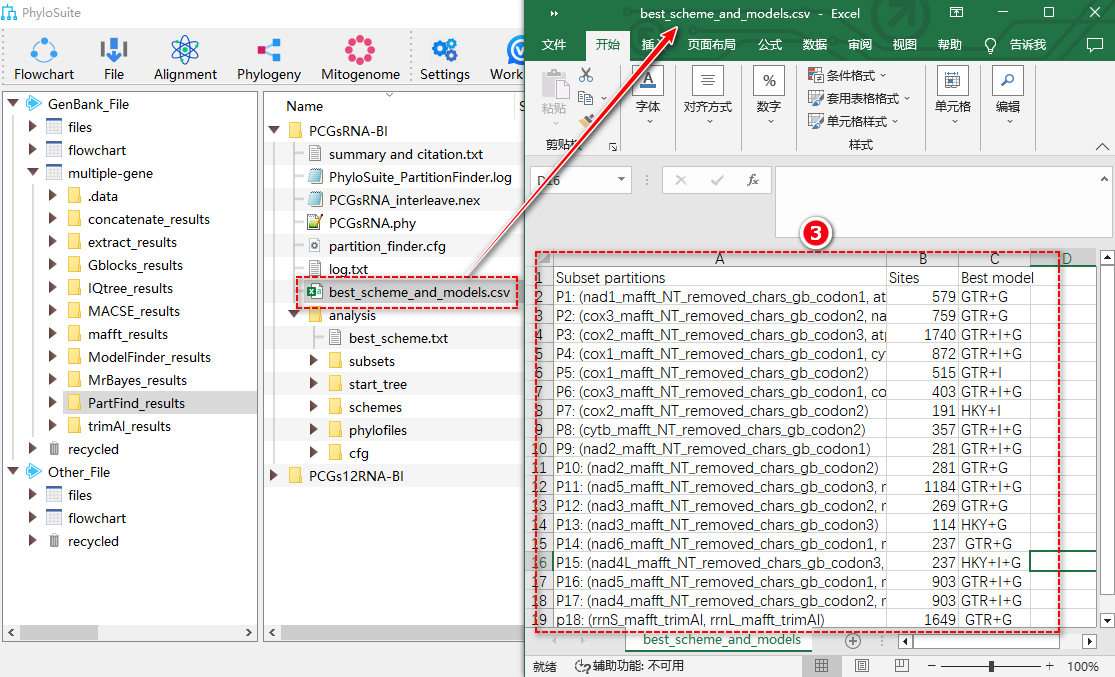


**Figure S17. The output files of** **PartitionFinder.**

3.3.1- 3.3.2 Alignment file (*.phy) and the preset partition file in text format (Figure S16).

3.3.3 “Subset partitions” describes the optimal partition strategy; “Sites” describes the total number of sites in each subset; “Best model” describes the best-fit models for each subset (Figure S17).

- 1. **The input file for MrBayes**


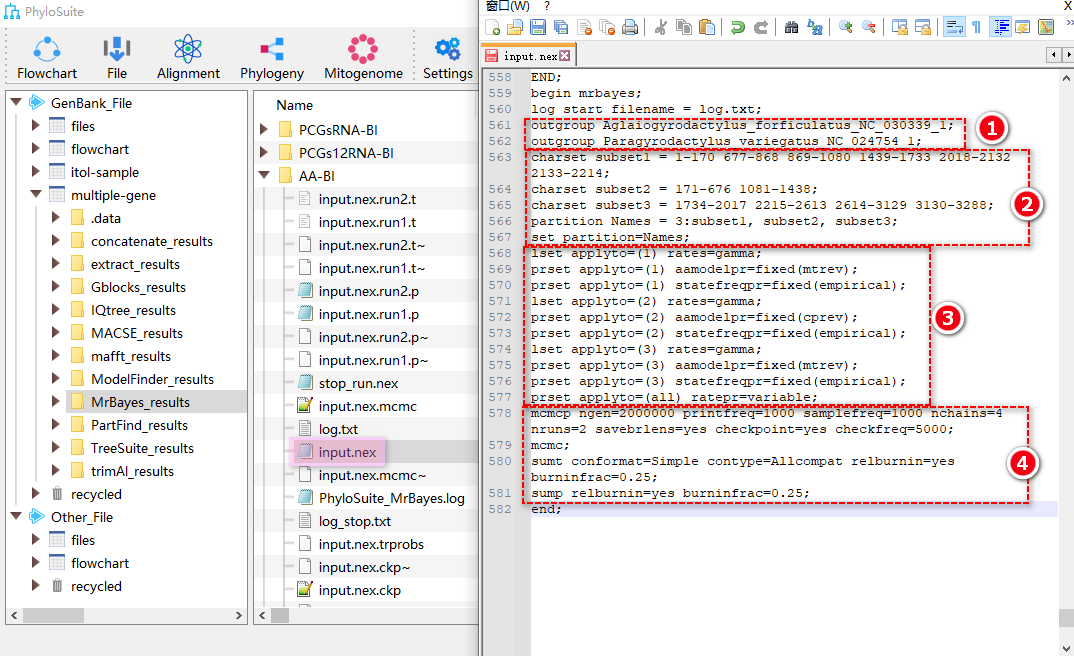


**Figure S18. The input file for MrBayes.**

3.4.1 Set outgroups.

3.4.2 Define subsets and provide the start-to-end sites for each partition.

3.4.3 Define the optional model for each partition.

3.4.4 Set parameters for MCMC, see section “Phylogenetic tree reconstruction based on the Bayesian inference (BI)” in the main document for details (Figure S18).

1. **Troubleshooting**

**4.1 Input files:** PhyloSuite uses standard file formats. A problem may occur if users put irrelevant files in the results folder generated by PhyloSuite. This may cause bugs if the newly added files contain the same suffix as the standard input file, as PhyloSuite automatically recognizes input files for downstream functions according to their names and suffixes.

**4.2 Bloated or corrupt Workplace folder:** As PhyloSuite permanently stores all of your results, the workplace folder may become very large with time and use. This may slow down PhyloSuite and even cause it to crash. If users experience such problems (slowness or crashing), please first try creating a new empty workplace folder, set it as the default workplace, and check whether PhyloSuite works fine now.

**4.3 trnL and trnS:** A common problem in studies of metazoan mitogenomes is the imprecise annotation of two tRNA genes: *trnL* and *trnS*. In more detail, commonly mitogenomes encode two trnL and two trnS genes, annotated as trnL1 and trnL2, and trnS1 and trnS2 respectively. If there is a *.fas file named “trnL” or “trnS” in the “tRNA” folder of the extraction result folder, users should try to annotate them anew.

**REFERENCES**

1. Xia, Xuhua. 2019. A Mathematical Primer of Molecular Phylogenetics. *Apple Academic Press*,

2. Yang, Ziheng and Bruce Rannala. 2012. Molecular phylogenetics: principles and practice. *Nature reviews genetics* <https://doi.org/10.1038/nrg3186>

3. Young, Andrew D. and Jessica P. Gillung. 2020. “Phylogenomics — principles, opportunities and pitfalls of big-data phylogenetics.” *Systematic Entomology* 45: 225-247. <https://doi.org/10.1111/syen.12406>

4. Abadi, Shiran, Dana Azouri, Tal Pupko and Itay Mayrose. 2019. “Model selection may not be a mandatory step for phylogeny reconstruction.” *Nat Commun* 10: 1-11. <https://doi.org/10.1038/s41467-019-08822-w>

5. Kalyaanamoorthy, S., B. Q. Minh, T. K. F. Wong, A. von Haeseler and L. S. Jermiin. 2017. “ModelFinder: fast model selection for accurate phylogenetic estimates.” *Nat Methods* 14: 587-589. <https://doi.org/10.1038/nmeth.4285>

6. Letunic, Ivica and Peer Bork. 2021. “Interactive Tree Of Life (iTOL) v5: an online tool for phylogenetic tree display and annotation.” *Nucleic Acids Res* 49: W293-W296. <https://doi.org/10.1093/nar/gkab301>
